# Design and Synthesis of a Compound Library Exploiting 5-Methoxyleoligin as Potential Cholesterol Efflux Promoter

**DOI:** 10.3390/molecules25030662

**Published:** 2020-02-04

**Authors:** Thomas Linder, Sophie Geyrhofer, Eleni Papaplioura, Limei Wang, Atanas G. Atanasov, Hermann Stuppner, Verena M. Dirsch, Michael Schnürch, Marko D. Mihovilovic

**Affiliations:** 1 Institute of Applied Synthetic Chemistry, TU Wien, Getreidemarkt 9/163, 1060 Vienna, Austria; thomas.linder@thermofisher.com (T.L.); sophie.geyrhofer@gmail.com (S.G.); eleni.papaplioura@tuwien.ac.at (E.P.); 2 Department of Pharmacology, School of Pharmacy, Qingdao University, 308 Ningxia Road, Qingdao 266071, China; limei.wang@qdu.edu.cn; 3 Department of Pharmacognosy, University of Vienna, Althanstraße 14, 1090 Vienna, Austria; atanas.atanasov@univie.ac.at (A.G.A.); verena.dirsch@univie.ac.at (V.M.D.); 4 Ludwig Boltzmann Institute for Digital Health and Patient Safety, Medical University of Vienna, Spitalgasse 23, 1090 Vienna, Austria; 5 Institute of Genetics and Animal Breeding of the Polish Academy of Sciences, Jastrzebiec, 05-552 Magdalenka, Poland; 6 Institute of Neurobiology, Bulgarian Academy of Sciences, 23 Acad. G. Bonchev str., 1113 Sofia, Bulgaria; 7 Institute of Pharmacy/Pharmacognosy, Center for Molecular Biosciences Innsbruck, University of Innsbruck, Innrain 80/82, 6020 Innsbruck, Austria; Hermann.Stuppner@uibk.ac.at

**Keywords:** natural product synthesis, cardiovascular diseases, lignans, macrophage cholesterol efflux

## Abstract

5-Methoxyleoligin and leoligin are natural occurring lignans derived from Edelweiss (*Leontopodium nivale* ssp. *alpinum*), displaying potent pro-angiogenic and pro-arteriogenic activity. Cholesterol efflux from macrophages is associated with reverse cholesterol transport which inhibits the development of cardiovascular disease. Within this study, we developed a modular and stereoselective total synthesis of 5-methoxyleoligin which can be readily used to prepare a novel compound library of related analogs. The target 5-methoxyleoligin was synthesized exploiting a recently disclosed modular route, which allows also rapid synthesis of analogous compounds. All obtained products were tested towards macrophage cholesterol efflux enhancement and the performance was compared to the parent compound leoligin. It was found that variation on the aryl moiety in 2-position of the furan ring allows optimization of the activity profile, whereas the ester-functionality does not tolerate significant alterations.

## 1. Introduction

According to the World Health Organization the number one cause of death results from ischemic heart disease, followed by stroke [1]. Both are examples of cardiovascular diseases (CVD), which is overall the leading cause of mortality and morbidity worldwide [2]. Thus, new strategies are required for the prevention and treatment of CVD. One key determinant within prevention is the lowering of cholesterol, especially low-density lipoprotein (LDL) cholesterol, in the body [3]. Uncontrolled uptake of modified lipoproteins, such as oxidized LDL (ox-LDL), and impaired cholesterol release contributes to foam cell formation in the arterial intima reflecting an early stage of atherosclerosis, which is the pathological basis of most CVD [4]. Especially macrophages contribute to foam cell formation via uptake of ox-LDL, cleavage of cholesterol esters, cholesterol re-esterification and accumulation. Cholesterol efflux from peripheral cells like macrophages is considered to be the first and rate-limiting step in reverse cholesterol out of the body [4]. Identification of compounds that promote cholesterol efflux from macrophages is therefore of high interest.

**Figure 1:**
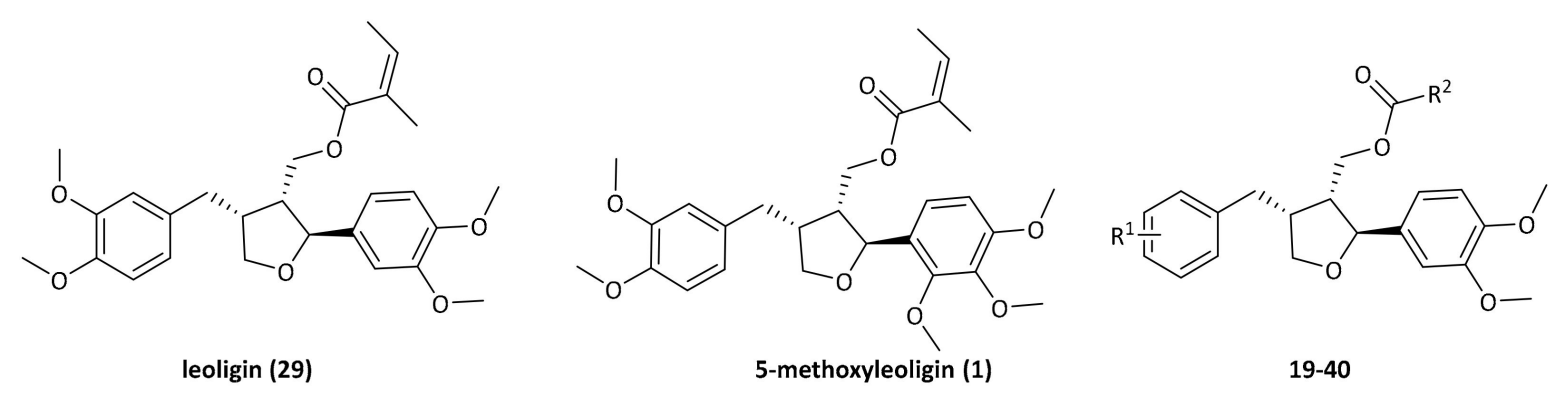
Structures of leoligin **18**, 5-methoxyleoligin **1** and general structure of derivatives **19**–**40**.

Natural products are still considered an attractive source of biologically and pharmacologically active compounds [5]. Within a recently conducted multi-disciplinary project on the identification of natural products with anti-inflammatory activity [6], in particular, naturally occurring lignans were quite early identified to exert various kinds of biological activity [7]. As an example, the Edelweiss-derived compound leoligin **29** (Figure 1) inhibits inflammation and selective cell proliferation in vitro and in vivo suggesting its use for the treatment of vein graft disease [8]. 5-Methoxyleoligin **1** (Figure 1), a furan-type lignan also isolated from Edelweiss stimulated angiogenesis in vitro and induced arteriogenesis in infracted rat hearts in vivo [9]. Thus, both Edelweiss lignans showed positive outcomes in models of CVD. In addition, leoligin promotes cholesterol efflux from human THP-1 macrophages in vitro [10]. Isolation of natural products is in most cases not feasible to provide required amounts for in vivo treatment. In particular, with Edelweiss being a strongly protected plant, a biosynthetic or purely synthetic pathway for its lignan constituents is required. A purely synthetic pathway would allow as well the preparation of 5-methoxyleoligin derivatives for creating a compound library of derivatives for medicinal chemistry investigations. Hence, within a multi-disciplinary project on the identification of natural products with anti-inflammatory activity [6], developing a fast, high yielding, and modular chemical synthesis of 5-methoxyleoligin and leoligin was tackled.

Although there are various lignan syntheses in the scientific literature [11,12,13,14,15] delivering scaffolds with the correct absolute configuration in enantiomerically pure form, 5-methoxyleoligin was never a target of one of these syntheses and was not obtained synthetically so far. One important reason for that is that those approaches were relatively long and not modular enough to quickly access another related compound apart from the targeted molecule.

The first modular synthesis to obtain leoligin and derivatives thereof was developed recently in our laboratory [15], however, the synthesis of 5-methoxyleoligin was not targeted in this contribution. Within this study, we address the synthesis of 5-methoxyleoligin, which required some adaptions as compared to our previously published synthetic route towards leoligin. Additionally, a number of leoligin derivatives has been synthesized and subjected to a macrophage cholesterol efflux assay to test whether they promote the efflux of cholesterol out of macrophages as previously shown for leoligin [10].

## 2. Results

**Scheme 1:**
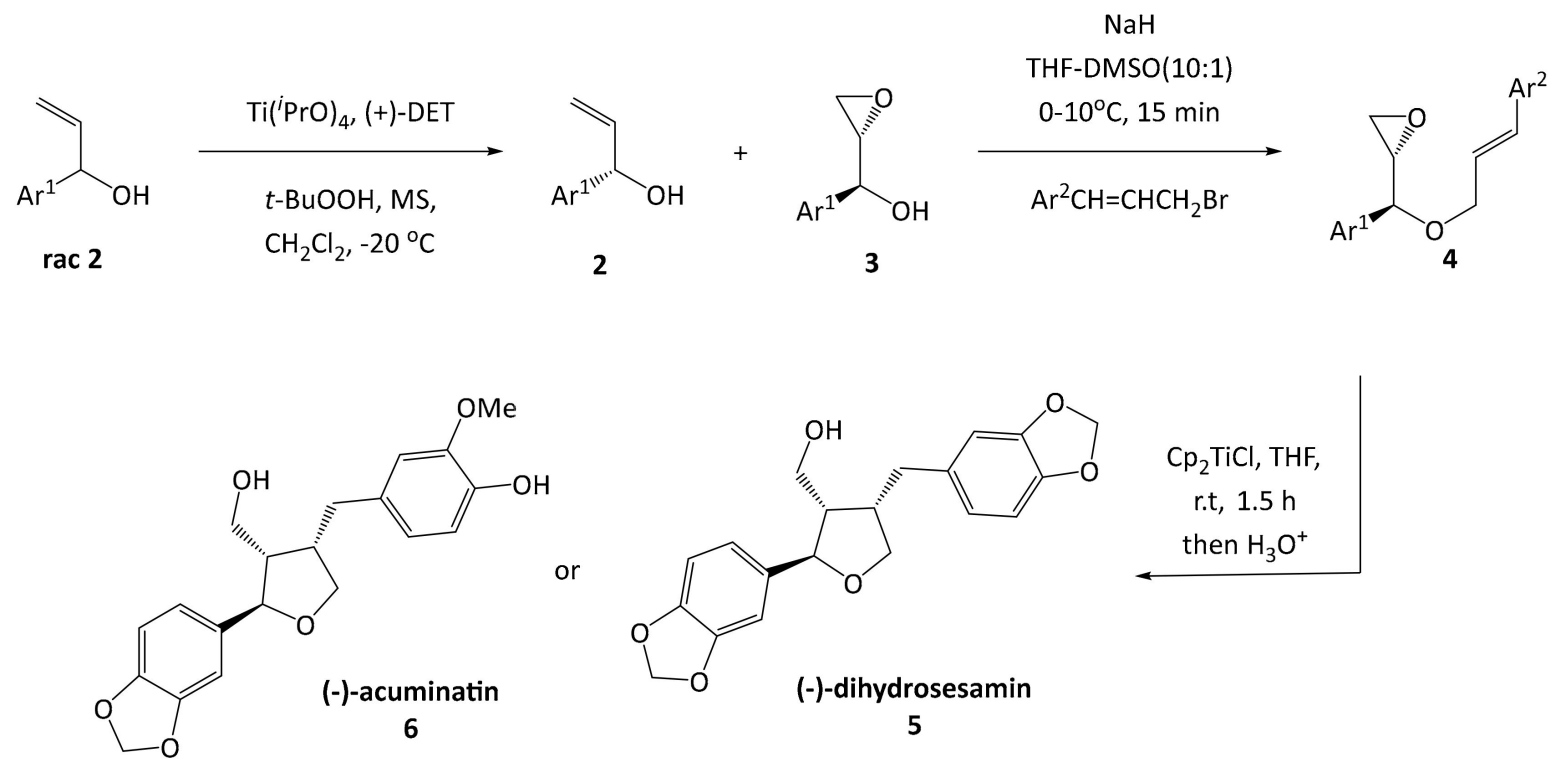
Synthesis of furan lignans (−)-dihydrosesamin **5** and (−)-acuminatin **6** by Roy, RajanBabu and Nugent.

Furan type lignans such as (−)-dihydrosesamin **5** and (−)-acuminatin **6** have been synthesized by the group of Roy [16], involving a kinetic resolution by a Sharpless asymmetric epoxidation, a Williamson ether synthesis and a radical cyclization reaction according to a protocol developed by RajanBabu and Nugent (Scheme 1) [17]. This methodology was previously used as entry point for developing the synthesis of leoligin in our group [15].

**Scheme 2:**
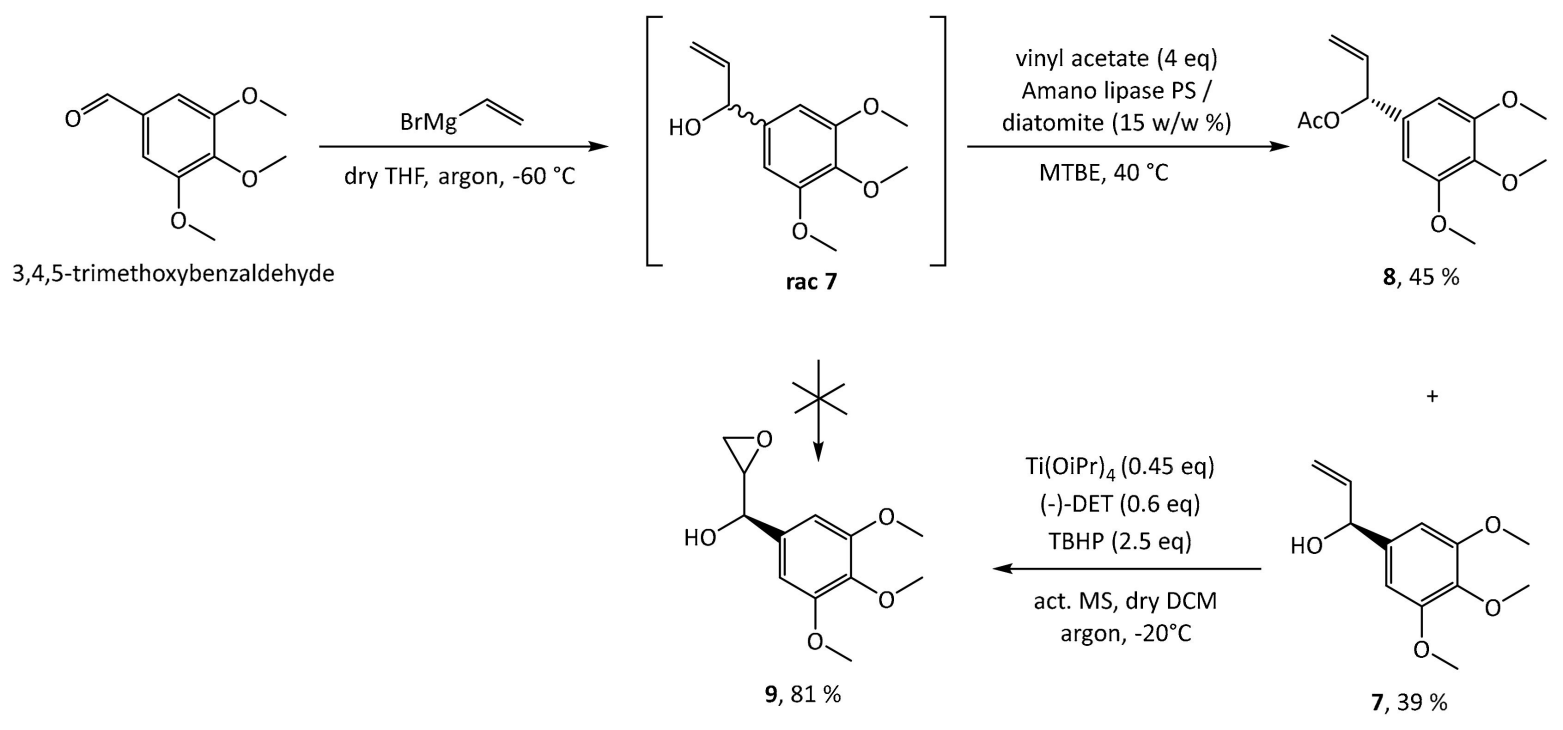
Synthesis of epoxy alcohol **9**.

In the route of Roy, the key intermediate for cyclization is an epoxy olefinic ether **4**, giving the furan scaffold in the desired configuration. Even though this protocol gave the desired compounds in good yield, each variation at the Ar^2^ substituent requires preparation of an individual epoxy olefinic ether **4** and subsequent cyclization. Since we are aiming for a modular synthesis, we envisioned a common cyclized intermediate, which can then be decorated with different Ar^2^ substituents in an efficient and simple procedure reducing the overall number of reaction steps for the synthesis of focused lignan libraries. This strategy was realized successfully in our total synthesis of leoligin (and the modularity was demonstrated in the preparation of several derivatives thereof) [15]. In the total synthesis of 5-methoxyleoligin **1**, the first step is a Grignard addition of vinylmagnesium bromide to commercially and cheaply available 3,4,5-trimethoxybenzaldehyde, producing a racemic mixture of the allylic alcohol **rac 7** as depicted in Scheme 2. In order to synthesize 5-methoxyleoligin **1** in its enantiomerically pure form, a chiral resolution had to be performed. Hence, the first approach was the subsequent Sharpless asymmetric epoxidation of the double bond. As this reaction is completely reagent controlled, it can be tuned to only convert the desired antipode, leaving the undesired one behind. Unfortunately, the Sharpless asymmetric epoxidation used as a method of chiral resolution, did not work as well as expected as it suffered from very low yields (<20%), when compared to the synthesis of the two natural products (−)-dihydrosesamin **5** and (−)-acuminatin **6** reported by Roy and Banerjee. Hence another method based on the enzyme Amano lipase PS was chosen, and the chiral resolution was carried out on the allylic alcohol **rac-7** before the epoxidation step. Additionally, this enzymatic step required optimization (see Table S1 in the Supplementary Materials) and finally the selective acetylation proceeded with good yield (39%) and high ee (>99%) for alcohol **7**. With respect to the synthesis of (−)-dihydrosesamin **5** and (−)-acuminatin **6**, where (*R*,*R*)-diethyltartrate (+)-DET was used, we obtained the desired epoxy alcohol **9** with the opposite stereochemistry by using (*S*,*S*)-diethyltartrate (−)-DET.

**Scheme 3:**
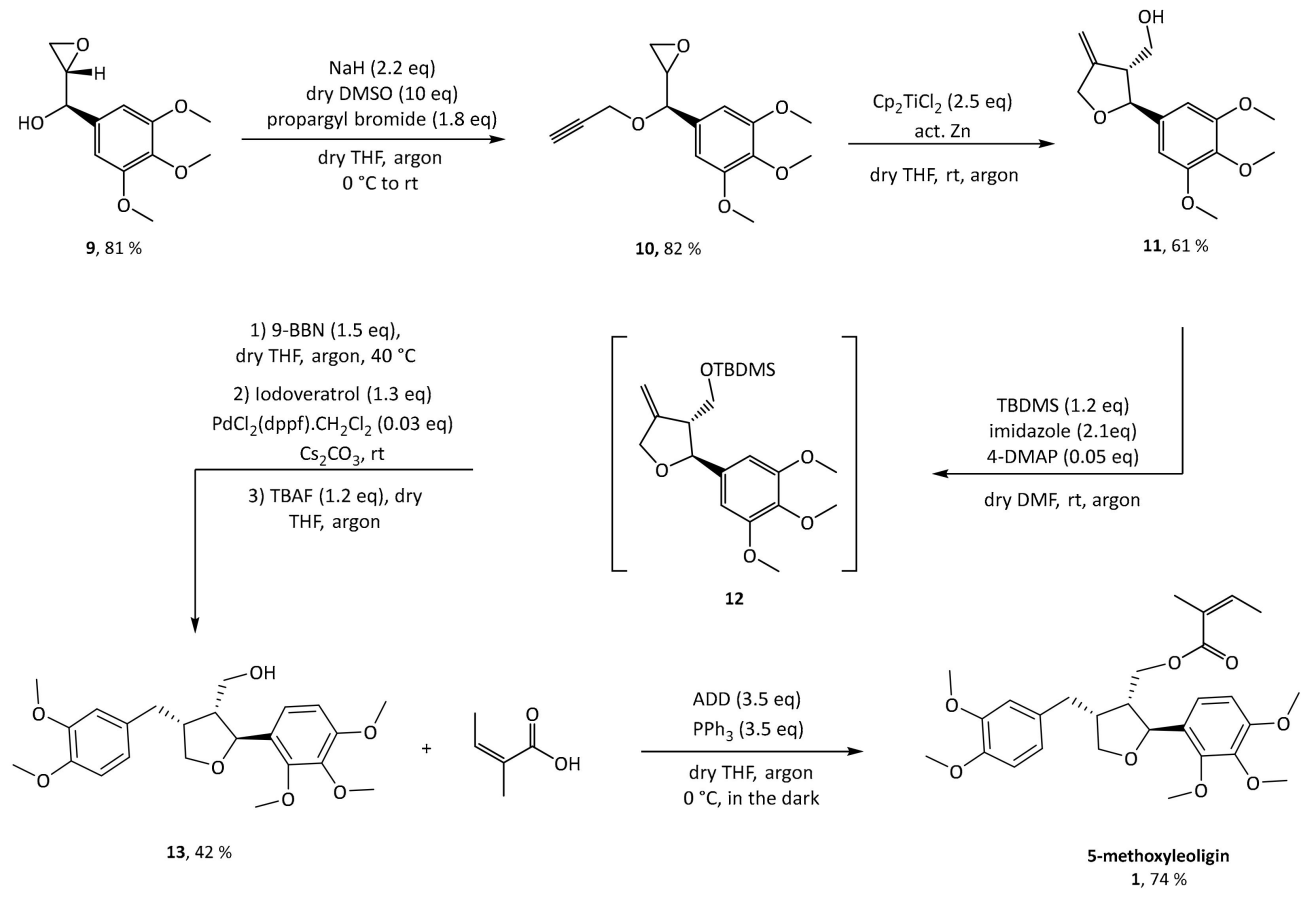
Synthesis of 5-methoxyleoligin **1**.

With the desired enantiomer in hand, we tried to synthesize 5-methoxyleoligin according to the previously published route for leoligin as shown in Scheme 3. In the synthesis of leoligin, the asymmetric epoxidation was carried out after an initial etherification by propargyl bromide. However, as the critical step, the radical cyclization reaction, did not work with our substrate in acceptable yield and all attempts to improve the situation failed, this approach was dismissed, and a variation to the initial route was investigated. In the present case of a starting material with an additional methoxy group, reversing the order of events was beneficial. As oxidation with various protocols (e.g., *m*CPBA-oxidation), fail on substrate **7** [18], Sharpless epoxidation was reconsidered. However, since it again suffered from low conversion and therefore low yields (around 20% according to GC), reaction conditions were screened regarding reaction temperature, time and catalyst loading. With respect to temperature, −20 °C is optimal, because decomposition of the starting material led to lower yield at higher temperatures. The second screen showed that the reaction is completed after 22 h, while longer reaction times again only lead to decomposition. Regarding catalyst loading, 45 mol% of catalyst gave satisfactory conversion, while further increase showed no improvement. The desired product **9** was obtained in ~80% yield, in reactions run at scale providing between 50 mg and 5 g of product.

Next, propargylation via a classical Williamson ether synthesis was performed leading to the corresponding substrate **10** with a yield of 82%. Switching to the terminal triple bond is important to ultimately enable access to the exocyclic olefin **11** after radical cyclization. This can then be used to introduce diverse aryl substituents in position 4 via a hydroboration and subsequent Suzuki–Miyaura cross-coupling with different aryl halides, making this the crucial step for enabling a modular synthesis. This reaction was performed according to conditions reported by Saha and Roy on a different synthetic target and the desired diastereomer was achieved in a yield of 61% of the desired diastereomer [19]. Protection of the hydroxyl group as silyl ether is mandatory both to prevent side reactions in the subsequent steps, and in particular to control the selectivity of the Suzuki–Miyaura cross-coupling reaction. Preliminary experiments showed that all tested silyl groups (*tert*-butyldimethylsilyl, *tert*-butyldiphenylsilyl, triisopropylsilyl) are large enough to provide sufficient sterical shielding to yield mainly the desired 3,4-*cis*-diastereomer, whereas smaller moieties such as an acetyl group are not sufficient [15]. Additionally, a silyl group only allows the distal carbon of the olefin to be attacked by the hydroboration reagent. For protection of the hydroxy functionality of **11**, *tert*-butyldimethylsilyl was used, the reaction was worked up and the protected crude product **12** was used without further purification in the subsequent hydroboration step. Hydroboration was carried out with 9-BBN, and without any work up or isolation all reagents for Suzuki coupling (base, catalyst and halide) were added directly to the reaction mixture. Initially, aqueous NaOH solution was used as base, which made work up necessary in order to get rid of the water before deprotection with TBAF. This was circumvented by switching to dry Cs_2_CO_3_, which gave slightly better yields and, more importantly, allowed to conduct the deprotection without a work-up, extending the one-pot reaction sequence.

Finally, with 5-methoxyleoligin alcohol **13** in hand, the only functionality left to be introduced to obtain 5-methoxyleoligin **1** was the angelic acid moiety. To preserve the integrity of the Z-configuration of the α,β-unsaturated system during the esterification, a Mitsunobu protocol with diethyl azodicarboxylate (DEAD) as reagent was used. Additionally, the reaction was carried out in the dark to minimize the possibility of isomerization.

**Figure 2:**
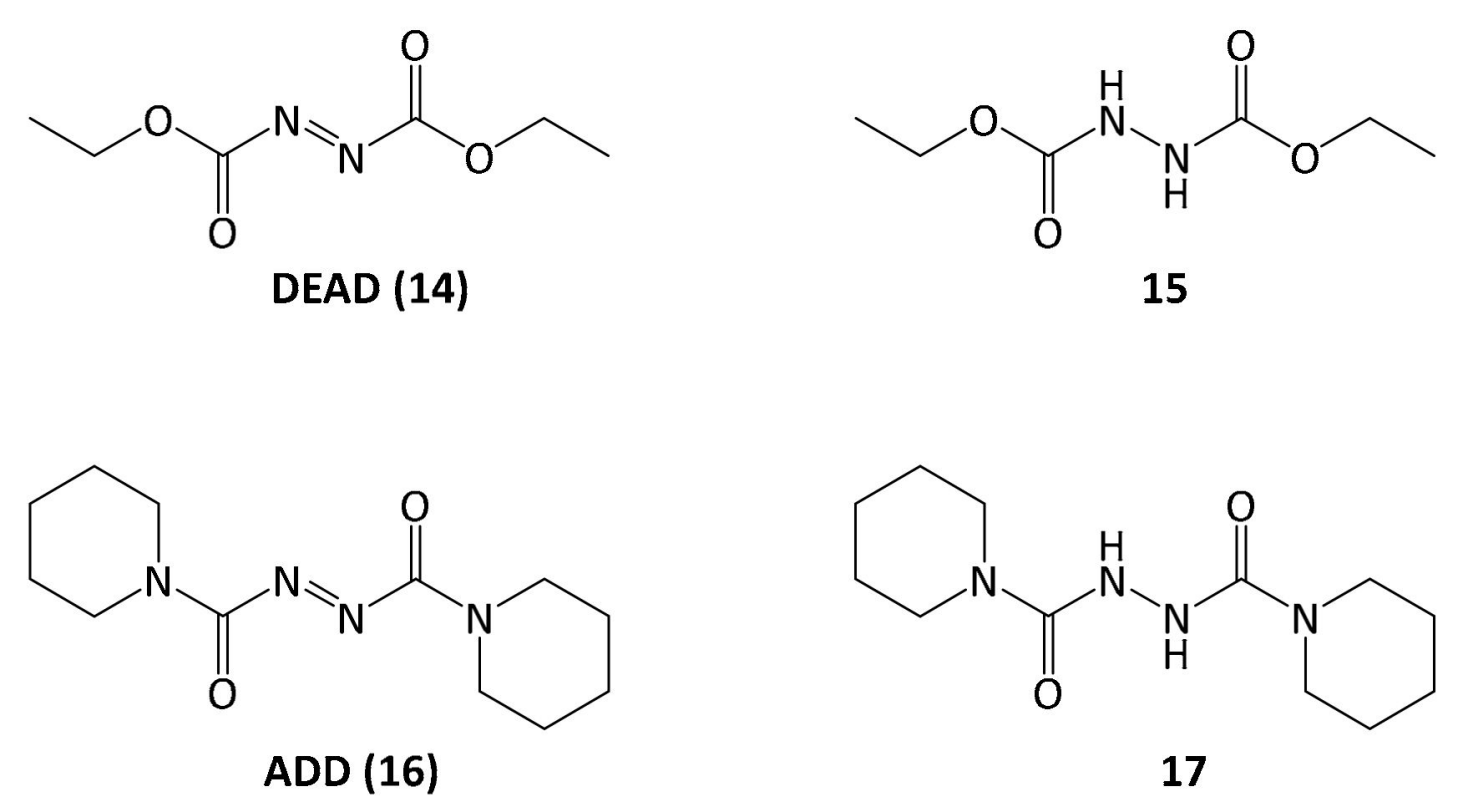
From left to right structures of diethyl azodicarboxylate (DEAD) and its reaction product **15** as well as 1,1′-(azodicarbonyl)dipiperidine **16** (ADD) and its reaction product **17**.

However, the reaction byproduct **15** of the diethyl diazenedicarboxylate reagent **14** (DEAD) had nearly the same retention time as the product, and after a separation on silica a second purification step was necessary, using reversed-phase preparative HPLC. In order to simplify purification, the Mitsunobu reaction was repeated with another reagent, 1,1′-(azodicarbonyl)dipiperidine **16** (ADD), which gave rise to a different reaction byproduct **17**, possessing significantly different polarity as compared to 5-methoxyleoligin simplifying the product isolation significantly (Figure 2).

Now, purification could easily be conducted via flash column chromatography in a single step and the product was obtained in 74% yield and an overall yield of 45% (starting from 25 g 3,4,5trimethoxybenzldehyde) over eight reaction steps.

**Figure 3:**
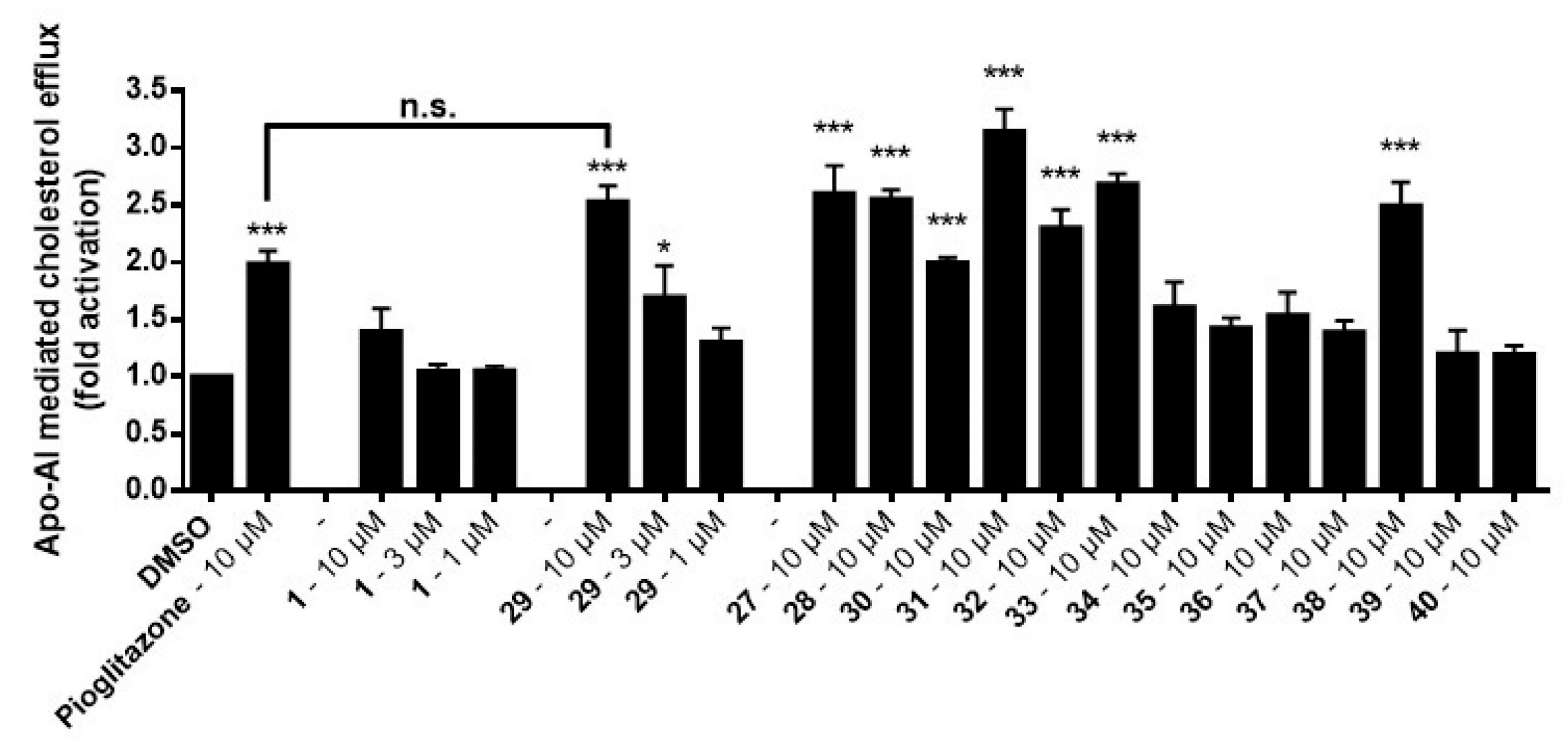
Results of leoligin **29** and 5-methoxyleologin **1** tested in a macrophage cholesterol efflux activation assay in THP-1 cells. The bars represent means ± SD of three independent experiments. *** *p* < 0.001, * *p* < 0.05 vs. the solvent vehicle control (*n* = 3, ANOVA/Bonferroni).

Having established the synthesis of 5-methoxyleoligin **1** within this work, and the synthesis of leoligin previously [15], both compounds and analogs thereof were tested for their potential to increase macrophage cholesterol efflux in an assay described in literature [10]. Differentiated (200 nM, PMA, 72 h) THP-1 macrophages were loaded for 24 h with (^3^*H*)-cholesterol and unesterified cholesterol in the presence of positive (10 μM, pioglitazone) or negative (0.1% DMSO) control or the indicted treatments (Figure 3). Cells were then incubated again for 6 h either in the presence or absence of apoA-I, an apolipoprotein which is the major protein component of high-density lipoprotein (HDL) acting as acceptor for cholesterol. Cells and medium were separated and the radioactivity in both fractions was determined. The percentage of cholesterol efflux was calculated by dividing the media-derived radioactivity by the sum of the radioactivity in the media and the cells. The specific apoA-I-mediated efflux was calculated as the difference between the efflux in the presence or absence of apoA-I (Figure 3).

**Scheme 4:**
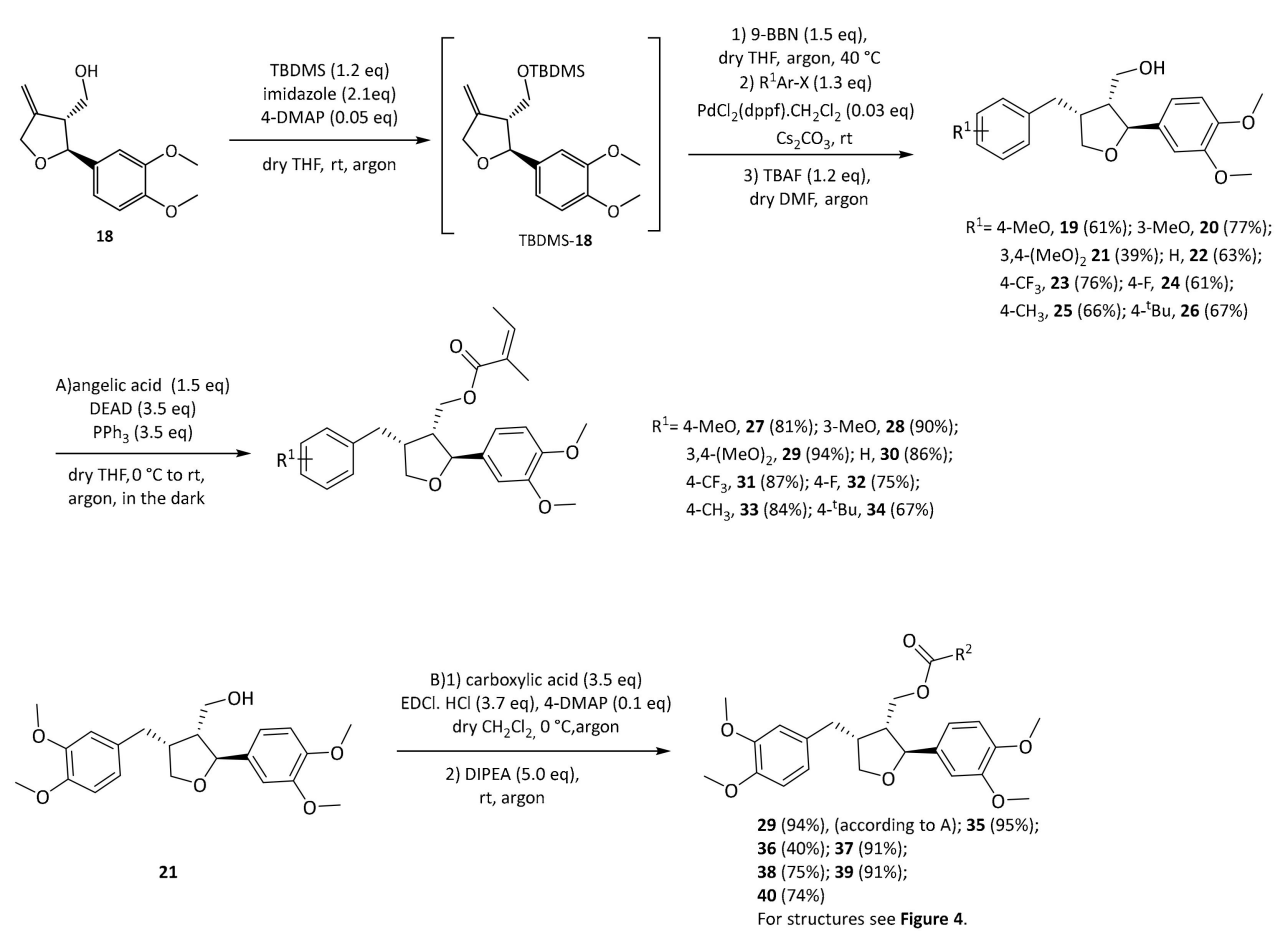
Synthesis of leoligin derivatives **19**–**40**.

As can be seen, leoligin **29** at 10 μM enhanced cholesterol efflux about 2.5-fold, whereas 5-methoxyleoligin **1** was inactive. In fact, leoligin **29** even outperformed the positive control pioglitazone in this experiment. In order to determine which structural features are important for this activity, a series of leoligin derivatives was prepared. Starting from alcohol **32** different aryl-substituents were introduced in position 4 (Scheme 4). As can be seen, the transformation is very robust giving good yields of alcohols **19**–**26** (39–77%) in all cases. Next, these alcohols were esterified with angelic acid giving the target compounds **27**–**33** in (67–90%) yield.

**Figure 4:**
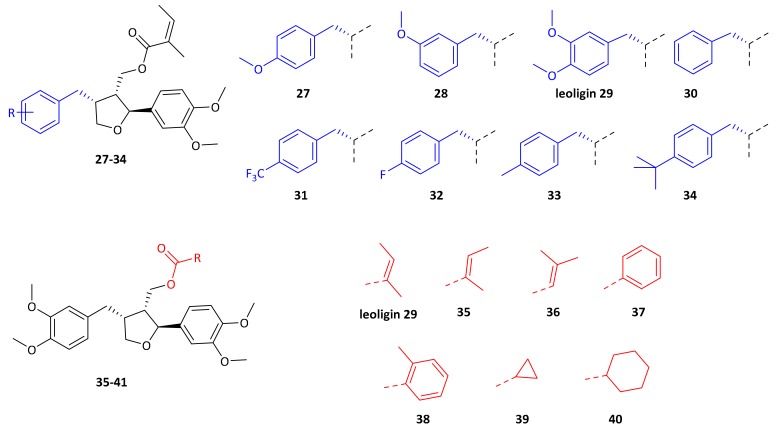
Structures of leoligin **29** and analog compounds tested in a macrophage cholesterol efflux activation assay.

Alcohol **21** (dimethyllariciresinol), the precursor to leoligin was then esterified with different acids towards target compounds **35**–**40**. For details on the synthesis see the supporting information. All obtained derivatives (displayed in Figure 4) were subjected to the same tests as leoligin (**29**) and 5-methoxyleoligin (**1**).

By simply moving one CH_3_-group in the ester functionality in a different position (see compounds **29** and **35**), cholesterol efflux enhancement is significantly less pronounced as compared to the original angelic acid in leoligin **29**. This already shows, that the biological activity is sensitive to even minor modifications in the ester functionality. Switching to a benzoic acid ester as in compound **37** has a similar effect. However, in case of the 2-methyl-benzoic acid ester, a similar activity compared to leoligin **29** was observed upon concomitant improved constitutional stability. This might be due to a similar geometry (see bold bonds in Figure 4) as compared to the angelic acid ester itself. Steric bulk might not be an issue, but the orientation in a kind of zig-zag mode might be required. Aliphatic ester as in **39** and **40** gave the least active compounds, indicating that a π-system seems to be important.

Next, modifications of the aryl group in position 4 were investigated. Compounds **27**–**34** were prepared according to our previously published protocol for the synthesis of leoligin **29** (see supporting information for details [15]).

Initially, either one of the two methoxy groups on that ring was removed, giving the para-MeO and meta-MeO compounds **27** and **28**. Both compounds still gave basically identical 2.5-fold cholesterol efflux enhancement as compared to leoligin **29**. Hence, it was tested whether both methoxy groups can be removed and the corresponding derivative **30** still showed significant enhancement of about 2.0-fold, however notably lower as compared to the other three compounds. Interestingly, having an electron-withdrawing substituent in para position of that ring, as in compound **31** with a CF_3_-group, event higher enhancement of about 3.1-fold was measured, the highest of all tested compounds so far. Also, a para-F group as in **32** showed significant enhancement,2.3-fold, however not as high as **31**. A relatively electron-neutral CH_3_-group as in **33**, with only a small +I effect, also gave 2.6-fold enhancement. Eventually, there is rather a specific steric-bulk required in this position, rather than a specific electronic effect. To test this hypothesis, we synthesized the *t*-butyl derivative **34**, which has an extremely large residue in that position. In this case, only 1.5-fold cholesterol efflux enhancement was observed, indicating that the limits of steric bulk tolerated were exceeded with this example.

## 3. Materials and Methods

### 3.1. General Information

Unless noted otherwise, reactants and reagents were purchased from commercial sources and used without further purification. Dry toluene, CH_2_Cl_2_, Et_2_O, THF and MeOH were obtained from a dispensing system by passing commercial material through a cartridge containing activated alumina (PURESOLV, Innovative Technology, Oldham, UK), stored under dry nitrogen and then used as such without further drying unless specified, while dry EtOH and DMF were purchased from a commercial source and used without further drying. DMSO was dried by treating commercial material with CaH_2_ mesh at 150 °C under argon, followed by distillation under reduced pressure. Deoxygenated and dry THF was obtained by refluxing and distilling pre-dried material (as described above) from sodium and benzophenone under argon. Zinc dust was activated by treating commercially available zinc dust with aqueous HCl (2 M), followed by thorough washing with water, subsequently with MeOH and dry Et_2_O. After drying in vacuo at 60 °C the material was stored under argon. Molecular sieves were activated by heating them to 200 °C for approximately 6 h in high vacuum and were then stored under argon. Melting ranges were determined using a Kofler-type Leica Galen III micro hot stage microscope or an SRS OptiMelt Automated Melting Point System (Stanford Research System, Sunnyvale, CA, USA) and are uncorrected. Temperatures are reported in intervals of 0.5 °C. Specific rotation was measured using an Anton Parr MCP500 polarimeter (Anton Paar GmbH, Graz, Austria) and HPLC grade solvents under conditions as specified individually. Values are reported in the form + or − specific rotation (concentration in terms of g/100 mL, solvent). Gas Chromatography-Mass Spectroscopy (GC-MS) were measured on a Thermo Scientific Finnigan Focus GC/Quadrupole DSQ II device (Fisher Scientific GmbH, Schwerte, Germany) using a helium flow of 2.0 mL/min, analyzing an m/z range from 50 to 650. HPLC was used to determine enantiomeric excess of reaction products, using a Dionex UltiMate 3000 device (RS Diode Array Detector, Fisher Scientific GmbH, Schwerte, Germany). Chiral separation columns and analysis conditions are specified individually. Liquid Chromatography-High Resolution Mass Spectroscopy (LC-HRMS) was measured either on a Shimadzu Prominence HPLC device (DGU-20 A3 degassing unit, 2 × LC-20AD binary gradient pump, SIL-20 A auto injector, CTO-20AC column oven, CBM-20A control module, and SPD-M20A diode array detector, Shimadzu Österreich, Korneuburg, Austria) or on an Agilent 1100/1200 HPLC device (degassing unit, 1200SL binary gradient pump, column thermostat, and CTC Analytics HTC PAL autosampler, (Agilent, CA, USA)). Flash column chromatography was carried out on Merck silica gel 60 (40–63 μm), and separations were performed using a Büchi Sepacore system (dual Pump Module C-605, Pump Manager C-615, Fraction Collector C-660, and UV Monitor C-630 or UV Photometer C-635, (BÜCHI Labortechnik AG, Flawil, Switzerland). Preparative HPLC was carried out on a Phenomenex Luna reversed-phase column (10 µm C18(2) phase, 100 A; 250 mm × 21.20 mm ID, (Phenomenex, Aschaffenburg, Germany), and separations were performed using a Shimadzu LC-8A device (SIL-10AP autosampler, SPD-20 detector, and FRC-10A fraction collector (Shimadzu Österreich, Korneuburg, Austria). NMR spectra were recorded from CDCl_3_ or DMSO-*d*_6_ solutions on a Bruker AC 200 (200 MHz proton resonance frequency, (Bruker Daltonik GmbH, Bremen, Germany) or a Bruker Advanced UltraShield (400 MHz) spectrometer (as indicated individually), and chemical shifts are reported in ascending order in ppm relative to the nominal residual solvent signals, i.e., ^1^H: δ = 2.50 ppm (DMSO-*d*_6_); ^13^C: δ = 77.16 ppm (CDCl_3_), δ = 39.52 ppm (DMSO-*d*_6_).

### 3.2. (S)-1-(3,4,5-Trimethoxyphenyl)prop-2-en-1-ol *(**7**)*

A round bottomed flask was charged with 3,4,5-trimethoxybenzaldehyde (25 g, 127.4 mmol, 1 equiv.) under argon atmosphere. Dry THF (175 mL, 0.73 M) was added and the solution was cooled to −60 °C. Vinylmagnesium bromide (146.5 mL, 146.5 mmol, 1.15 equiv.) was added drop wise via a dropping funnel over 2 h while the temperature was kept nearly constant (±5 °C). Reaction progress was monitored by TLC. When the reaction was finished, the mixture was then allowed to warm to −10 °C. Satd. aq. NH_4_Cl solution (30 mL) was added drop wise over 15 min and the temperature was maintained below -10 °C. Subsequently water (130 mL) was added to dissolve the magnesium salts and the product was extracted with Et_2_O (1 × 150 mL, 2 × 75 mL). The combined organic layers were washed with satd. aq. NaHCO_3_ solution (45 mL) and brine (30 mL), dried over Na_2_SO_4_ and filtered through a plug of silica (5 g, preconditioned with Et_2_O). The solvent was removed in vacuo and the product was dried in vacuo without further purification. The purity of the product was determined by H-NMR (>95%).^1^H-NMR (200 MHz, CDCl_3_): δ 1.95 (s, 1H, OH), 3.82 (s, 3H, OCH_3_), 3.86 (s, 6H, 2 × OCH_3_), 5.13 (d, *J* = 5.87 Hz, 1H, H2), 5.16–5.43 (m, 2H, H4), 5.93–6.13 (m, 1H, H3), 6.59 (s, 2H, H2′ and H6′). **Rac 7** (28.3 g, 126.2 mmol, 1 equiv.) was dissolved in MTBE (690 mL, 0.18 M) and vinyl acetate (40.5 mL, 504.8 mmol, 4 equiv.). The solution was kept at 40.5 °C and Amano lipase PS on diatomite (3.68 g, 13 *w/w*%) was added. Reaction progress was monitored by chiral HPLC. After 76 h at this temperature the enantiomeric separation was complete, and the mixture was filtered through Celite 545. The solvent was removed in vacuo and the compounds were separated by column chromatography (MPLC, 2 × 90 g silica in sequence, 50 mL/min flow rate, 12% EtOAc for 50 min, then 12–100% in 60 min). Compound **7** was obtained as a slightly yellow oil with an overall yield of 39% and >99% ee. ^1^H-NMR (200 MHz, CDCl_3_): δ 1.95 (bs, 1H, OH), 3.82 (s, 3H, OCH_3_), 3.86 (s, 6H, 2 × OCH_3_), 5.13 (d, *J* = 5.87 Hz, 1H, CH-OH), 5.16–5.43 (m, 2H, CH=CH_2_), 5.93–6.13 (m, 1H, CH-CH_2_), 6.59 (s, 2H, CH-C-CH). ^13^C-NMR (50 MHz, CDCl_3_): δ 56.01 (q 3′ OCH_3_ & 5′ OCH_3_), 60.76 (q, 4′ OCH_3_), 75.34 (d, CH-OH), 103.08 (d, CH-C-CH), 115.19 (t, CH=CH_2_), 138.31 (s, C_q_), 139.97 (d, CH=CH_2_), 153.24 (s, *m*-C_aryl_). One C_q_ not visible.

### 3.3. (1R)-Oxiran-2-yl(3,4,5-trimethoxyphenyl)methanol *(**9**)*

Dry DCM (150 mL), (−)-DET (2.789 g, 12.536 mmol, 0.6 equiv.) and **7** (5.085 g, 22.676 mmol, 1 equiv.) were (additionally) dried over activated MS overnight under argon atmosphere. (−)-DET was dissolved in dried DCM (1 mL) and cooled to −20 °C via a cryostat. Ti(OiPr)_4_ (3.00 mL, 10.145 mmol, 0.45 equiv.) in dry DCM (70 mL, 0.14 M) was added and the reaction mixture was stirred for 15 min. Then TBHP (5.5 M in decane, 10.31 mL, 56.689 mmol, 2.5 equiv.) was added slowly. After 30 min the solution of **7** was added and the resulting mixture was stirred for 70 h at −20 °C. Reaction progress was monitored by TLC. When the reaction was finished, a solution of sodium sulfite (20 g in 100 mL water) was added as well as 1000 mL DCM and 500 mL water. The aqueous layer was extracted with DCM (4 × 500 mL) and the combined organic layers were dried over Na_2_SO_4_ and filtered. The solvent was removed in vacuo and the compound was purified via column chromatography (MPLC, product rotated on BULK Isolute Sorbent, 90 g silica, 50 mL/min flow rate, 8% EtOAc for 15 min, then 8–50% EtOAc in 25 min, then 50–100% EtOAc in 10 min, then 100% for 10 min) and the product **8** was obtained with 81% yield as an orange oil. ^1^H-NMR (200 MHz, CDCl_3_): δ 2.36 (d, *J* = 2.16 Hz, 1H, OH), 2.77 (dd, *J* = 4.89 & 4.10 Hz, 1H, CH_2_O), 2.93 (dd, *J* = 5.09 & 2.74 Hz, 1H, CH_2_O), 3.16–3.24 (m, 1H, CH_2_-CH-O), 3.82 (s, 3H, OCH_3_), 3.85 (s, 6H, 2 × OCH_3_), 4.77-4.83 (m, 1H, CH-CH-O), 6.60 (s, 2H, CH-C-CH). ^13^C-NMR (50 MHz, CDCl_3_): δ 43.7 (t, CH_2_), 55.0 (d, CH-O-CH_2_), 56.1 (q, 2 × OCH_3_), 60.8 (q, OCH_3_), 71.1 (d, CH-OH), 103.2 (d, CH-C-CH), 135.2 (s, C_q_), 153.4 (s, 2C, *m*-C_aryl_). One C_q_ not visible.

### 3.4. 2-((R)-(Prop-2-yn-1-yloxy)(3,4,5-trimethoxyphenyl)methyl)oxiran *(**10**)*

NaH (60% dispersion in mineral oil, 2.2 equiv.) was dissolved in dry THF (1M with respect to NaH) under argon atmosphere and cooled with an ice bath. Dry DMSO (10 equiv.) was added. To this suspension substrate **9** (4.431 g, 18.443 mmol) added slowly as a solution in dry THF (0.4 M with respect to **9**). After stirring for 15 min a solution of 3-bromopropyne in THF was added slowly, usually followed by additional THF to dissolve the formed slurry. The ice bath was then removed, and the reaction mixture was stirred for 48 h. Progress of the reaction was monitored by TLC until complete conversion was indicated. The solution was cooled to 0 °C again and HCl (1M, 1 equiv.) was added drop wise. Most of the THF was then removed in vacuo and water was added. The aqueous layer was extracted with 4 × Et_2_O, the combined organic layers were washed with brine, dried over Na_2_SO_4_, filtered, and the solvent was removed. The crude product was purified via column chromatography (MPLC, 90 g silica, 50 mL/min flow rate, 3% EtOAc for 10 min, then 3–30% EtOAc in 20 min, then 30–100% EtOAc in 20 min, then 100% EtOAc for 10 min). Product 10 was obtained as slightly yellow oil with an overall yield of 82%. ^1^H-NMR (200 MHz, CDCl_3_): δ 2.46 (t, *J* = 2.35 Hz, 1H, CH≡C-CH_2_), 2.76 (dd, *J* = 5.19 & 2.54, 1H, CH-CH_2_-O), 2.84 (dd, *J* = 5.19 & 3.91, 1H, CH-CH_2_-O), 3.12–3.20 (m, 1H, CH-CH_2_-O), 3.85 (s, 3H, OCH_3_), 3.88 (s, 6H, 2 × OCH_3_), 4.01 (dd, *J* = 15.66 & 2.35 Hz, 1H, C-CH_2_-O), 4.24 (dd, *J* = 15.66 & 2.34 Hz, 1H, C-CH_2_-O), 4.43 (d, *J* = 4.70 Hz, 1H, CH-CH-O), 6.60 (s, 2H, CH-C-CH).

^13^C-NMR (50 MHz, CDCl_3_): δ 45.5 (t, CH-CH_2_-O), 54.0 (d, CH-CH_2_-O), 56.1 (t, C-CH_2_-O), 56.1 (q, 2 × OCH_3_), 60.8 (q, OCH_3_), 74.9 (d, C≡CH), 79.2 (s, C≡CH), 79.7 (d, CH-CH-O), 104.2 (d, CH-C-CH), 132.7 (s, *p*-C_aryl_), 138.0 (s, CH-C-CH), 153.4 (s, *m*-C_aryl_).

### 3.5. ((2S,3R)-4-Methylene-2-(3,4,5-trimethoxyphenyl)tetrahydrofuran-3-yl) methanol *(**11**)*

For the synthesis of **11** a round bottom flask was charged with act. Zn (2.306 g, 35.264 mmol, 7 equiv.) and Cp_2_TiCl_2_ (3.135 g, 12.594 mmol, 2.5 equiv.) under argon; deoxygenated THF (60 mL, distilled from Na/benzophenone) was added. After 1 h of stirring at rt, the Zn was allowed to settle for 5 min and the solution (without the Zn) was transferred to a solution of **10** (1.402 g, 5.038 mmol, 1 equiv.) in deoxygenated THF (40 mL) over a period of 25 min. Stirring was continued for 70 min at rt and reaction progress was monitored by TLC. When the reaction was completed, diluted sulfuric acid (10% in water, 30 mL) was added and the major amount of THF was evaporated. Water was added to the crude product and the aqueous layer was extracted with Et_2_O (4 × 200 mL). The combined organic layers were washed with satd. NaHCO_3_ solution, brine, dried over Na_2_SO_4_, filtered and the solvent was removed in vacuo. The crude product was purified via column chromatography (MPLC, 90 g silica, 50 mL/min flow rate, 10–50% EtOAc for 20 min, then 50–100% in 15 min, then 100% for 15 min) to yield **11** as a yellow oil with 61% yield. ^1^H-NMR (200 MHz, CDCl_3_): δ 1.62 (bs, 1H, OH), 2.69–85 (m, 1H, H3), 3.82 (s, 3H, OCH_3_), 3.85 (s, 8H, 2 × OCH_3_ & CH_2_O), 4.35–4.69 (m, 2H, H5), 4.78 (d, *J* = 7.44 Hz, 1H, H2), 5.02-5.16 (m, 2H, CH_2_), 6.63 (s, 2H, H2′ & H6′). ^13^C-NMR (50 MHz, CDCl_3_): δ 54.0 (d, C4), 56.0 (q, 2 × OCH_3_), 60.7 (q, OCH_3_), 61.9 (t, CH_2_O), 71.4 (d, C3), 83.4 (d, C2), 103.5 (d, C2′ & C6′), 105.0 (t, CH_2_), 136.8 (s, C_q_), 148.5 (d, C3), 153.5 (s, C3′ & C5′). One C_q_ not visible. HRMS (ESI^+^): for C_15_H_20_O_5_ [M + Na]^+^: 303.1203. Found: 303.1203.

### 3.6. ((2S,3R,4S)-4-(3,4-Dimethoxyphenyl)-2-(3,4,5-trimethoxyphenyl)tetrahydrofuran-3-yl)methanol *(**13**)*

Substrate **11** (1.014 g, 3.617 mmol, 1 equiv.), imidazole (0.518 g, 7.596 mmol, 2.1 equiv.) and 4-DMAP (23.1 mg, 0.181 mmol, 0.05 equiv.) were dissolved in DMF (21 mL, 0.17 M) under argon. TBDMSCl (3 M in THF, 1.45 mL, 4.341 mmol, 1.2 equiv.) was added to the solution and the mixture was stirred for 16 h at rt. Reaction progress was monitored by TLC. When the reaction was finished, Et_2_O (50 mL) and satd. NH_4_Cl solution (20 mL) were added and the aqueous phase was extracted with Et_2_O (4 × 30 mL). The combined organic layers were washed with satd. NaHCO_3_ solution (10 mL) and brine (10 mL), dried over Na_2_SO_4_ and filtered. The solvent was evaporated, and the crude product used without purification in the next step. A flask was charged with 11 (3.617 mmol, 1 equiv.) under argon and 9-BBN (0.5 M in THF, 10.85 mL, 5.426 mmol, 1.5 equiv.) was added. The resulting mixture was stirred for 22.5 h at 40 °C. On the next day, it was allowed to warm to rt and aqueous NaOH solution (1 M, 10 mL) was added. Stirring was continued for another 15 min and 4-iodoveratrole (1.248 g, 4.792 mmol, 1.3 equiv.) and PdCl_2_(dppf).CH_2_Cl_2_ (88.5 mg, 0.109 mmol, 0.03 equiv.) were added. The mixture became biphasic and was stirred for another 24 h at rt, then Et_2_O (100 mL) and brine (25 mL) were added. Reaction progress was monitored by TLC. The layers were separated, and the aqueous phase was extracted with Et_2_O (4 × 30 mL). The combined organic layers were dried over Na_2_SO_4_, filtered and the solvent was evaporated. The crude product **12** was used without further purification in the next step. A flask was charged with I (3.617 mmol, 1 equiv.) under argon and TBAF (1 M in THF, 4.34 mL, 4.341 mmol) was added. The reaction was stirred for 20 h at rt. Reaction progress was monitored by TLC. When the reaction was finished, Et_2_O (100 mL) and brine (25 mL) were added and the aqueous phase was extracted with Et_2_O (4 × 30 mL) and EtOAc (2 × 35 mL). The combined organic layers were dried over Na_2_SO_4_, filtered and the solvent was evaporated. The compound was purified via column chromatography (MPLC, 90 g silica, 40 mL/min flow rate, 30 EtOAc for 5 min, then 30–100% EtOAc in 45 min). Product **13** was obtained as a yellow oil with a total yield of 42% over 3 steps. ^1^H-NMR (200 MHz, CDCl_3_): δ 1.68 (bs, 1H, OH), 2.32–2.82 (m, 3H, H3 & H4 & CH_2_), 2.92 (dd, *J* = 12.82 & 4.60 Hz, 1H, CH_2_), 3.83 (s, 3H, OCH_3_), 3.85 (s, 15H, 4 × OCH_3_, CH_2_O, H5), 4.07 (dd, *J* = 8.41 & 6.45 Hz, 1H, H5), 4.84 (d, *J* = 6.06 Hz, 1H, H2), 6.56 (s, 2H, H2′ & H6′), 6.67–6.83 (m, 3H, H2′′ & H5′′ & H6′′). ^13^C-NMR (50 MHz, CDCl_3_): δ 33.1 (t, CH_2_), 42.2 (d, C4), 52.4 (d, C3), 55.8 (q, 2 × OCH_3_), 56.1 (q, 2 × OCH_3_), 60.8 (q, OCH_3_), 60.9 (t, CH_2_OH), 73.0 (t, C5), 82.9 (d, C2), 102.5 (d, C2′ & C6′), 111.2 (d, CH), 111.8 (d, CH), 120.4 (d, C6′′), 132.9 (s, C1′′), 137.05 (s, C_q_), 138.7 (s, C_q_), 147.4 (s, C4′′), 148.9 (s, C3′′), 153.2 (s, C3′ & C5′). HRMS (ESI^+^): for C_23_H_3_O_7_ [M + Na]^+^: 441.1884. Found: 441.1903.

### 3.7. (Z)-((2S,3R,4S)-4-(3,4-Dimethoxyphenyl)-2-(3,4,5-trimethoxyphenyl)tetrahydrofuran-3-yl)methyl 2-methylbut-2-enoate (***1***, i.e., 5-Methoxyleoligin)

Substrate **13** (152.6 mg, 0.365 mmol), angelic acid (1.5 equiv.) and PPh_3_ (3.5 equiv.) were charged under argon, cooled with an ice bath and dissolved in THF (0.13 M). ADD (3.5 equiv.) was added slowly and the reaction was stirred for 18.5 h in the dark while being allowed to warm to rt. Reaction progress was monitored by TLC. When the reaction was finished, brine was added, the layers separated, and the aqueous phase was extracted with 3 × Et_2_O. The combined organic layers were dried over Na_2_SO_4_, filtered and the solvent was removed via evaporation. The crude product was purified via column chromatography (MPLC, 40 g silica, 50 mL/min flow rate, 10–22% EtOAc in 10 min, then 22% EtOAc for 10 min, then 22–65% EtOAc in 40 min. %-Methoxyleoligin **1** was yielded as a slightly yellow viscous oil with 74% yield. ^1^H-NMR (200 MHz, CDCl_3_): δ 1.83–1.90 (m, 3H, α-CH_3_), 1.95–2.04 (m, 3H, β-CH_3_), 2.47–2.81 (m, 3H, H3 & H4 & CH_2_), 2.88 (dd, 1H, CH_2_), 3.77 (dd, *J* = 8.70 & 6.26 Hz, 1H, H5), 3.81 (s, 3H, OCH_3_), 3.84 (s, 6H, 2 × OCH_3_), 3.85 (s, 6H, 2 × OCH_3_), 4.07 (dd, *J* =8.70 & 6.07 Hz, 1H, H5), 4.29 (dd, *J* = 11.35 & 7.14, 1H, CH_2_O), 4.43 (dd, *J* = 11.35 & 6.56 Hz, 1H, CH_2_O), 4.83 (d, *J* = 5.87, 1H, H2), 6.02–6.17 (m, 1H, β-CH), 6.54 (s, 2H, H2′ & H6′), 6.64–6.83 (m, 3H, H2′′ & H5′′ & H6′′). ^13^C-NMR (50 MHz, CDCl_3_): δ 15.8 (q, β-CH_3_), 20.5 (q, α-CH_3_), 33.12 (t, CH_2_), 42.56 (d, C4), 49.2 (d, C3), 55.8 (q, OCH_3_), 55.9 (q, OCH_3_), 56.1 (q, C3′ OCH_3_ & C5′ OCH_3_), 60.8 (q, C4′ OCH_3_), 62.2 (t, CH_2_O), 72.8 (t, C5), 83.1 (d, C2), 102.5 (d, C2′ & C6′), 111.3 (d, C2′′), 111.8 (d, C5′′), 120.4 (d, C6′′), 127.2 (s, α-C), 132.7 (s, C1′′), 138.2 (s, C_q_), 139.0 (d, β-CH), 147.5 (s, C4′′), 148.9 (s, C3′′), 153.3 (s, C3′ & C5′), 167.6 (s, C=O). One C_q_ not visible [20].

## 4. Conclusions

In conclusion, the first enantiopure total synthesis of 5-methoxyleoligin was established, which can in addition readily be used readily for synthesizing a library of related lignans. Although there are several approaches regarding the synthesis of the cyclization intermediate **6** reported in the literature, the kinetic resolution using Amano lipase PS was newly introduced, improving the ee of the product and facilitating the transformation into a dynamic kinetic resolution in the future. The diastereoselective hydroboration and subsequent Suzuki-coupling enables the rapid generation of a compound library which is not literature known on systems like 5-methoxyleoligin so far, therefore representing a new methodology.

The target compound 5-methoxyleoligin **1** was tested towards macrophage cholesterol efflux, however no significant effect was observed in contrast to leoligin **29**, which showed 2.5-fold enhancement at 10 μM. Several ester derivatives were tested, showing limited flexibility in this position. However, the 2-methylbenzoic acid ester showed identical activity, which is very interesting for further investigations, since this ester is much more stable as compared to the angelic acid ester. This may in particular become important within future in vivo-studies. Additionally, variations at the aryl ring in position 4 were investigated. Interestingly, the electronic effects of substituents on that ring seem to play a negligible role and the obtained results suggest a more pronounced steric effect. In the future, the effect of varying the substituents on the aryl moiety in position 2 of the furan ring will be investigated to eventually further improve cholesterol efflux.

## Supplementary Materials

The following are available online, Experimental details for all prepared compounds including analytical data.
