# Supplementary material for: Design and Synthesis of a Compound Library Exploiting 5-Methoxyleoligin as Potential Cholesterol Efflux Promoter
Source: Molecules. 2020 Feb 4;25(3):662. doi: 10.3390/molecules25030662 (PMC7038131; doi:10.3390/molecules25030662)
Supplement: Supplementary file 1 [file molecules-25-00662-s001.pdf]

molecules MDPI

Supporting Information

Design and Synthesis of a Compound Library Exploiting 5-Methoxyleoligin as Potential Cholesterol Efflux Promoter

Thomas Linder[1], Sophie Geyrhofer[1], Eleni Papaplioura[1], Limei Wang[2], Atanas G. Atanasov[3], Verena Dirsch[1], Michael Schnürch[1], Marko D. Mihovilovic*

1 Institute of Applied Synthetic Chemistry, TU Wien, Getreidemarkt 9/163, 1060 Vienna, Austria; thomas.linder@thermofisher.com (T.L.), sophie.geyrhofer@gmail.com (S.G.), eleni.papaplioura@tuwien.ac.at (E.P.)

2 Department of Pharmacology, School of Pharmacy, Qingdao University, Qingdao, China; limei.wang@qdu.edu.cn

3 Department of Pharmacognosy, University of Vienna, Althanstraße 14, 1090 Vienna, Austria; atanas.atanasov@univie.ac.at (A.G.A.), verena.dirsch@univie.ac.at (V.D.)

4 Ludwig Boltzmann Institute for Digital Health and Patient Safety, Medical University of Vienna, Spitalgasse 23, 1090 Vienna, Austria.

5 Institute of Genetics and Animal Breeding of the Polish Academy of Sciences, Jastrzebiec, 05-552 Magdalenka, Poland.

6 Institute of Neurobiology, Bulgarian Academy of Sciences, 23 Acad. G. Bonchev str., 1113 Sofia, Bulgaria.

7 Institute of Pharmacy/Pharmacognosy, Center for Molecular Biosciences Innsbruck, University of Innsbruck, Innrain 80/82, 6020 Innsbruck, Austria; Hermann.Stuppner@uibk.ac.at (H.S.)

* Correspondence: michael.schnuerch@tuwien.ac.at (M.S.), marko.mihovilovic@tuwien.ac.at (M.D.M.); Tel.: +43 (1) 58801-163616 (M.S.)

General Notes ... 3

Table 1. Results of reaction condition optimization of the Sharpless epoxidation. ... 6

General Procedure A (GPA) ... 7

General Procedure B (GPB) ... 7

A. Synthesis of Intermediate Compounds ... 8

A.1 (±)-1-(3,4,5-Trimethoxyphenyl)prop-2-en-1-ol (rac 7) ... 8

A.2 Enantiomeric separation of (R)- and (S)-1-(3,4,5-trimethoxyphenyl)prop-2-en-1-ol (7&8)[8]... 8

A.3 (1R)-Oxiran-2-yl(3,4,5-trimethoxyphenyl)methanol (9) ... 10

A.4 2-((R)-(Prop-2-yn-1-yloxy)(3,4,5-trimethoxyphenyl)methyl)oxiran (10) ... 11

A.5 ((2S,3R)-4-Methylene-2-(3,4,5-trimethoxyphenyl)tetrahydrofuran-3-yl)methanol (11) ... 12

A.6 *tert*-Butyldimethyl(((2S,3R)-4-methylene-2-(3,4,5-trimethoxyphenyl)tetrahydrofu- ran-3-yl)methoxy)silane (12) ... 12

A.7 *tert*-Butyl(((2S,3R,4R)-4-(3,4-dimethoxybenzyl)-2-(3,4,5-trimethoxyphenyl) tetrahydrofuran-3-yl)methoxy)dimethylsilane (I) ... 13

A.8 ((2S,3R,4S)-4-(3,4-Dimethoxyphenyl)- 2-(3,4,5-trimethoxyphenyl)tetrahydro-3-yl) methanol (13) ... 14

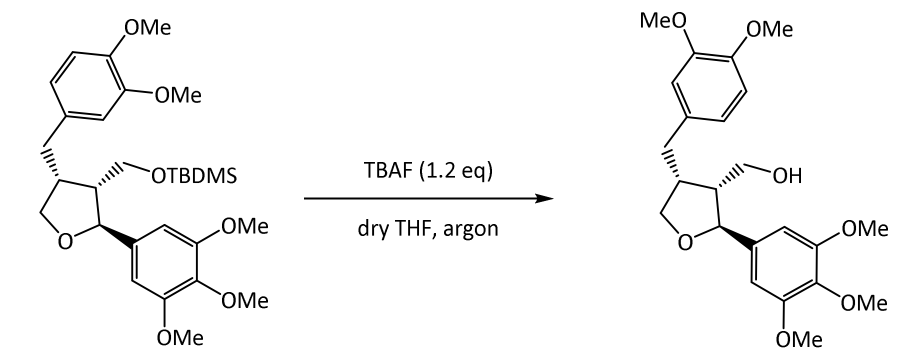

I
Chemical Formula: $C_{29}H_{44}O_7Si$
Molecular Weight: 532.74

13
Chemical Formula: $C_{23}H_{30}O_7$
Molecular Weight: 418.48

.... 14

A.9 (Z)-((2S,3R,4S)-4-(3,4-Dimethoxyphenyl)- 2-(3,4,5-trimethoxyphenyl)tetrahydro- 15

furan -tetrahydrofuran-3-yl)methyl 2-methylbut-2-enoate (1) [9] 15

B. Synthesis of leoligin analogs 16

B.1 (E)-((2S,3R,4R)-4 -(3,4-Dimethoxybenzyl)-2-(3,4-dimethoxyphenyl)tetrahydrofuran -3-yl)methyl 2-methylbut-2-enoate (35) 16

B.2 ((2*S*,3*R*,4*R*)- 4-(3,4-Dimethoxybenzyl)-2-(3,4-dimethoxyphenyl)tetrahydrofuran-3- 17

yl) methyl 3-methylbut-2-enoate (36) 17

B.3 ((2*S*,3*R*,4*R*)- 4-(3,4-Dimethoxybenzyl)-2-(3,4-dimethoxyphenyl)tetrahydrofuran-3- 19

yl)methyl benzoate (37) 19

B.4 ((2*S*,3*R*,4*R*)- 4-(3,4-Dimethoxybenzyl)-2-(3,4-dimethoxyphenyl)tetrahydrofuran-3- 20

yl)methyl 2-mehylbenzoate (38) 20

B.5 ((2*S*,3*R*,4*R*)- 4-(3,4-Dimethoxybenzyl)-2-(3,4-dimethoxyphenyl)tetrahydrofuran-3- 21

yl)methyl cuyclopropanecarboxylate (39)... 21

B.6 ((2*S*,3*R*,4*R*)- 4-(3,4-Dimethoxybenzyl)-2-(3,4-dimethoxyphenyl)tetrahydrofuran-3................. 22

-yl)methyl cyclohexanecarboxylate (40).. 22

B.7 ((2*S*,3*R*,4*R*)- 2-(3,4-Dimethoxybenzyl)-4-(4-methoxybenzyl)tetrahydrofuran-3-yl) 23

methanol (19) 23

B.8 ((2*S*,3*R*,4*R*)- 2-(3,4-Dimethoxybenzyl)-4-(3-methoxybenzyl)tetrahydrofuran-3-...................... 24

yl)methanol (20)... 24

B.9 ((2S,3R,4R)- 4--(3,4-Dimethoxyphenyl)-2- (3,4-dimethoxyphenyl)tetrahydrofuran-3-yl)methanol (21)... 25

B.10 ((2S,3R,4R)- 4-4-Benzyl-2-(3,4-dimethoxyphenyl)tetrahydrofuran-3-yl)methanol (22)......... 26

B.11 (2S,3R,4R)-2-(3,4-Dimethoxyphenyl)-4-(4-(trifluoromethyl)benzyl)tetrahydro 27

furan-3- yl)methanol (23)... 27

B.12 ((2S,3R,4R)-2-(3,4-Dimethoxyphenyl)-4-(4-fluorobenzyl)tetrahydrofuran-3-yl) 28
methanol (24) 28
B.13 ((2S,3R,4R)-2-(3,4-Dimethoxyphenyl)-4-(4-methylbenzyl)tetrahydrofuran-3-yl) 29
methanol (25) 29
B.14 ((2S,3R,4R)-4-(4-(tert-Butyl)benzyl)-2-(3,4-dimethoxyphenyl)tetrahydrofuran-.................... 30
3-yl) methanol (26).. 30
B.15 Z)-((2S,3R,4R)-2-(3,4-Dimethoxyphenyl)-4-(4-methoxybenzyl)tetrahydrofuran-3 31
-yl)methyl 2-methylbut-2-enoate (27)... 31
B.16 (*Z*)-((2*S*,3*R*,4*R*)-2-(3,4-Dimethoxyphenyl)-4-(3-methoxybenzyl)tetrahydrofuran-3 32
-yl)methyl 2-methylbut-2-enoate (28)... 32
B.17 (*Z*)-((2*S*,3*R*,4*R*)-4-(3,4-Dimethoxybenzyl)-2-(3,4- dimethoxyphenyl) tetrahy-drofuran -3-yl)methyl 2-methylbut-2-enoate (leoligin, 29) 33
B.18 (*Z*)-((2*S*,3*R*,4*R*)-4-Benzyl-2-(3,4-dimethoxyphenyl)tetrahydrofuran-3-yl)methyl 2-methylbut-2-enoate (30) 34
B.19(*Z*)-((2*S*,3*R*,4*R*)-2-(3,4-Dimethoxyphenyl)-4-(4-(trifluoromethyl)benzyl) 35
tetrahydrofuran-3-yl)methyl 2-methylbut-2-enoate (31).. 35
B.20 (*Z*)-((2*S*,3*R*,4*R*)-2-(3,4-Dimethoxyphenyl)-4-(4-fluorobenzyl)tetrahydrofuran-3.................... 36
-yl)methyl 2-methylbut-2-enoate (32)... 36
B.21 (*Z*)-((2*S*,3*R*,4*R*)-2-(3,4-Dimethoxyphenyl)-4-(4-methylbenzyl)tetrahydrofuran-3-................. 37
yl)methyl 2- methylbut-2-enoate (33) 37
B.22 (*Z*)-((2*S*,3*R*,4*R*)-4-(4-(*tert*-Butyl)benzyl)-2-(3,4-dimethoxyphenyl)tetrahydro....................... 38
furan-3-yl)methyl 2-methylbut-2-enoate (34) 38
References 40

General Notes

Chemicals

Unless noted otherwise, reactants and reagents were purchased from commercial sources and used without further purification.

Dry toluene, CH_2Cl_2, Et_2O, THF and MeOH were obtained from a dispensing system by passing commercial material through a cartridge containing activated alumina (PURESOLV, Innovative Technology), stored under dry nitrogen and then used as such without further drying unless specified.

Dry EtOH and **DMF** were purchased from a commercial source and used without further drying.

DMSO was **dried** by treating commercial material with CaH_2 mesh at 150 °C under argon, followed by distillation under reduced pressure.[1]

Deoxygenated and dry THF was obtained by refluxing and distilling pre-dried material (as described above) from sodium and benzophenone under argon.[2]

Zinc dust was **activated** by treating commercially available zinc dust with aqueous HCl (2 M), followed by thorough washing with water, subsequently with MeOH and dry Et_2O. After drying *in vacuo* at 60 °C the material was stored under argon.[3]

Molecular sieves were activated by heating them to 200 °C for approximately 6 h in high vacuum and were then stored under argon.[4]

Melting ranges were determined using a Kofler-type Leica Galen III micro hot stage microscope or an SRS OptiMelt Automated Melting Point System and are uncorrected. Temperatures are reported in intervals of 0.5 °C.

Aluminum-backed Merck silica gel 60 with fluorescence indicator F_{254} was used for **Thin Layer Chromatography** (TLC). Spots were visualized under UV light (254 nm) and by staining with cerium ammonium molybdate (CAM) solution (20 g of ammonium pentamolybdate, 0.8 g of cerium(IV) ammonium sulfate, 400 mL of 10 v/v % sulfuric acid) as a general purpose reagent. Alcohols were also visualized with *p*-anisaldehyde solution (3.5 g *p*-anisaldehyde, 1.5 mL acetic acid, 5 mL sulfuric acid, 120 mL ethanol), and compounds pertaining double bonds were visualized with potassium permangante solution (1.5 g potassium permanganate, 10 g potassium carbonate, 1 mL 10 w/w % NaOH, 200 mL water).

Specific rotation was measured using an Anton Parr MCP500 polarimeter and HPLC grade solvents under conditions as specified individually. Values are reported in the form + *or - specific rotation* (*concentration in terms of g / 100 mL*, *solvent*).

Analytical Chromatography-Spectroscopy

Gas Chromatography-Mass Spectroscopy (GC-MS) was used to analyze samples of reaction products with sufficient volatility. The following instruments and columns were used:

Instrument: Thermo Scientific Finnigan Focus GC / Quadrupole DSQ II device using a helium flow of 2.0 mL / min, analyzing an *m/z* range from 50 to 650.

Column: BGB 5 (0.25 µm film; 30 m x 0.25 mm ID)

Temperature gradients are as follows:

Method A: 100 °C (2 min), to 280 °C in 10 min, 11 min hold-time at 280 °C (23 min)

Method B: 80 °C (2 min), to 280 °C in 10 min (20 °C / min), 12 min hold-time at 280 °C (24 min)

Method C: 100 °C (2 min), to 280 °C in 4.5 min (40 °C / min), 16.5 min hold-time at 280 °C (23 min)

Method D: 100 °C (2 min), to 280 °C in 4.5 min (40 °C / min), 31.5 min hold-time at 280 °C (38 min)

Method E: 100 °C (2 min), to 280 °C in 4.5 min (40 °C / min), 41.5 min hold-time at 280 °C (48 min)

Data is reported in the form *retention time*; m/z_1 (*relative intensity in %*), m/z_2 (*relative intensity in %*), … Only signals with $m/z \geq 90$ and relative intensity ≥ 15 % are given, except for the signal at 100 % relative intensity which is always given. Also, the molecular ion signal M^+ is given regardless of its intensity or *m/z*; in cases where M^+ was not visible due to excessive fragmentation, a characteristic fragment signal is identified instead.

High Pressure Liquid Chromatography (HPLC) was used to determine enantiomeric excess of reaction products, using a Dionex UltiMate 3000 device (RS Diode Array Detector). Chiral separation columns and analysis conditions are specified individually. In all cases, retention times include appropriate guard cartridges containing the same stationary phase as the separation column.

Liquid Chromatography-High Resolution Mass Spectroscopy (LC-HRMS) was used to confirm exact molecular mass of reaction products by their quasi-molecular ions ($M+H^+$ or $M+Na^+$). The following two instruments were used:

Instrument 1: Shimadzu Prominence HPLC device (DGU-20 A3 degassing unit, 2 x LC-20AD binary gradient pump, SIL-20 A auto injector, CTO-20AC column oven, CBM-20A control module, and SPD-M20A diode array detector). Samples were eluted through a Phenomenex Kinetex precolumn (5 µm core shell ODS(3) phase; 4 mm x 2 mm ID) at 40 °C under conditions comprising gradients of H_2O / MeOH containing formic acid (0.1 v/v %), and then detected using a Shimadzu IT-TOF-MS by Electrospray Ionization (ESI) or Atmospheric Pressure Chemical Ionization (APCI), as indicated individually. Analyses were performed by E. Rosenberg (CTA, VUT) and L. Czollner (IAS, VUT).

Instrument 2: Agilent 1100/1200 HPLC device (degassing unit, 1200SL binary gradient pump, column thermostat, and CTC Analytics HTC PAL autosampler). Samples were eluted through a silica-based Phenomenex C-18 Security guard cartridge (1.7 µm PD; 2.1 mm ID) at 40 °C under isocratic conditions comprising H_2O containing formic acid (0.1 v/v %) / MeOH containing formic acid (0.1 v/v %) in a ratio of 30 : 70 at a flow rate of 0.5 mL / min, and then detected using an Agilent 6230 LC-TOF-MS equipped with an Agilent Dual AJS ESI source by Electrospray Ionization (ESI). Analyses were performed by L. Czollner (IAS, VUT).

Preparative chromatography

Flash column chromatography was carried out on Merck silica gel 60 (40-63 µm), and separations were performed using a Büchi Sepacore system (dual Pump Module C-605, Pump Manager C-615, Fraction Collector C-660, and UV Monitor C-630 or UV Photometer C-635).

Preparative High Peformance Liquid Chromatography (preparative HPLC) was carried out on a Phenomenex Luna reversed-phase column (10 µm C18(2) phase, 100 A; 250 mm x 21.20 mm ID), and separations were performed using a Shimadzu LC-8A device (SIL-10AP autosampler, SPD-20 detector, and FRC-10A fraction collector).

Reaction temperatures were measured externally (electronic thermometer connected to heater-stirrer or low temperature thermometer in case of cryogenic reactions) unless otherwise noted.

Partition coefficients (**log *P*** values) were calculated using ACD/Labs 12 with LogP Accuracy Extender.

Nuclear Magnetic Resonance (NMR) spectroscopy

NMR spectra were recorded from $CDCl_3$ or DMSO-d_6 solutions on a Bruker AC 200 (200 MHz proton resonance frequency) or a Bruker Advanced UltraShield (400 MHz) spectrometer (as indicated individually), and chemical shifts are reported in ascending order in ppm relative to the nominal residual solvent signals, i.e. ^{1}H: δ = 2.50 ppm (DMSO-d_6); ^{13}C: δ = 77.16 ppm ($CDCl_3$), δ = 39.52 ppm (DMSO-d_6).[5, 6] For all ^{1}H spectra in $CDCl_3$, however, shifts are reported relative to TMS as internal standard (δ = 0 ppm) due to the interference of aromatic signals of many samples with the residual solvent signal of $CDCl_3$. For ^{13}C spectra, *J*-modulated (APT) or DEPT-135 pulse sequences were used to aid in the assignment.

Table 1. Results of reaction condition optimization of the Sharpless epoxidation.

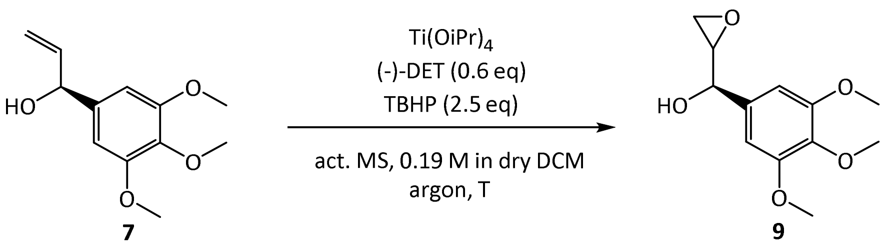

Entry #	Temperature [°C]	Time [h]	Catalyst [mol%]	Batch size [g]	Yield [%]
1	0	22	45	0.1	18
2	-20	7	45	0.05	66
3	-20	22	45	0.05	82
4	-20	30	45	0.05	80
5	-20	70	45	0.05	80

6	-20	22	15	0.05	26
7	-20	22	45	0.05	54
8	-20	22	90	0.05	53
9	-20	22	45	0.1	22
10	-20	22	45	1	67
11	-20	22	45	2	75
12	-20	22	45	5	81

General Procedure A (GPA)

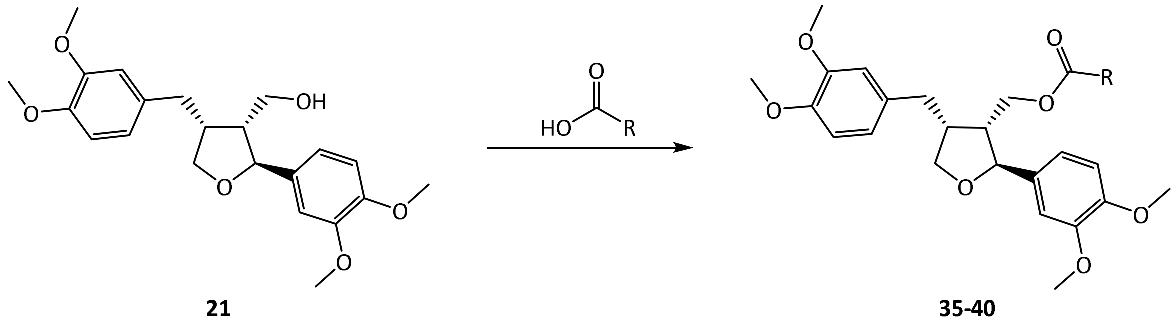

All compounds **35-40** in this section were prepared according to the General Outline above. Thus, for the preparation of any particular compounds, the experimental details are given in full in the analogous procedure below.

Generally, reaction progress was monitored by TLC or GC-MS, and the reaction was terminated when complete. Details for work-up and purification are given for each case individually to afford compounds of structure **35-40**.

General Procedure B (GPB)

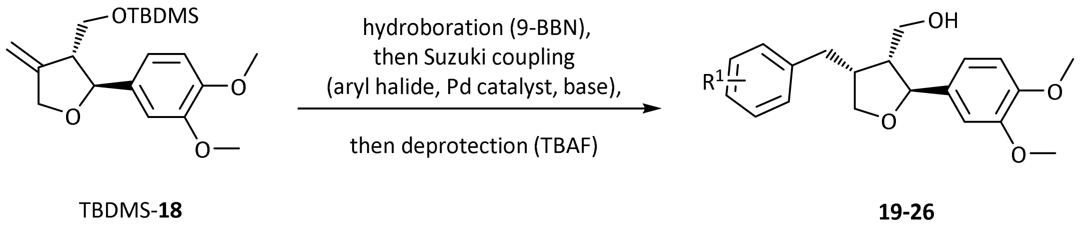

In this section, crude material TBDMS-**18** from the previous silyl protection [7] was used. Molar amounts of TBDMS-**18** are thus based on complete-conversion calculations in the protection step. However, masses of TBDMS-**18** correspond to the actual gross weight of staring material as used. Yields are calculated over all four steps (protection, hydroboration, coupling and deprotection.)

All compounds **19-26** in this section were prepared according to the General Procedure A above and are arranged such that compounds with the same R^1 are grouped together. However, certain variations exist with respect to the experimental details, and a single general procedure is therefore not readily stated in more detail. Thus, for the preparation of any particular compound, the experimental details are either given in full, or the reader is referred

to an analogous procedure already described for a compound in this section. Generally, reaction progress was monitored by TLC and the reaction was terminated when compete or when no further conversion was observed. Details for wok-up and purification are given for each case individually to afford compounds of structure **33-39**.

A. Synthesis of Intermediate Compounds

A.1 (±)-1-(3,4,5-Trimethoxyphenyl)prop-2-en-1-ol (rac 7)

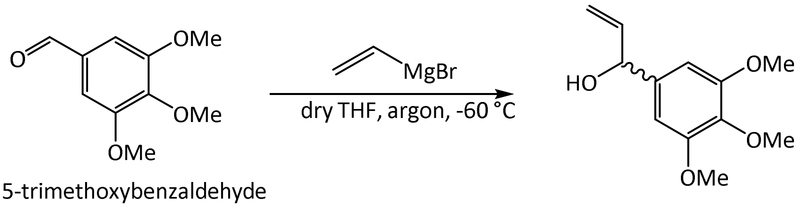

3,4,5-trimethoxybenzaldehyde
Chemical Formula: $C_{10}H_{12}O_4$
Molecular Weight: 196,20

rac 7
Chemical Formula: $C_{12}H_{16}O_4$
Molecular Weight: 224,25

A round bottomed flask was charged with 3,4,5-trimethoxybenzaldehyde (25 g, 127.4 mmol, 1 equiv.) under argon atmosphere. Dry THF (175 mL, 0.73 M) was added and the solution was cooled to -60 °C. Vinylmagnesium bromide (146.5 mL, 146.5 mmol, 1.15 equiv.) was added drop wise via a dropping funnel over 2 h while the temperature was kept nearly constant (± 5 °C). Reaction progress was monitored by TLC. When the reaction was finished, the mixture was then allowed to warm to -10 °C. Satd. aqu. NH_4Cl solution (30 mL) was added drop wise over 15 min and the temperature was maintained below +10 °C. Subsequently water (130 mL) was added to dissolve the magnesium salts and the product was extracted with Et_2O (1 x 150 mL, 2 x 75 mL). The combined organic layers were washed with satd. aqu. $NaHCO_3$ solution (45 mL) and brine (30 mL), dried over Na_2SO_4 and filtered through a plug of silica (5 g, preconditioned with Et_2O). The solvent was removed in *vacuo* and the product was dried in *vacuo* without further purification. The purity of the product was determined by H-NMR (> 95 %).

^{1}H-NMR (200 MHz, $CDCl_3$): δ 1.95 (s, 1H, OH), 3.82 (s, 3H, OCH_3), 3.86 (s, 6H, $2xOCH_3$), 5.13 (d, J= 5.87 Hz, 1H, H2), 5.16-5.43 (m, 2H, H4), 5.93-6.13 (m, 1H, H3), 6.59 (s, 2H, H2' & H6')

A.2 Enantiomeric separation of (R)- and (S)-1-(3,4,5-trimethoxyphenyl)prop-2-en-1-ol (7&8)[8]

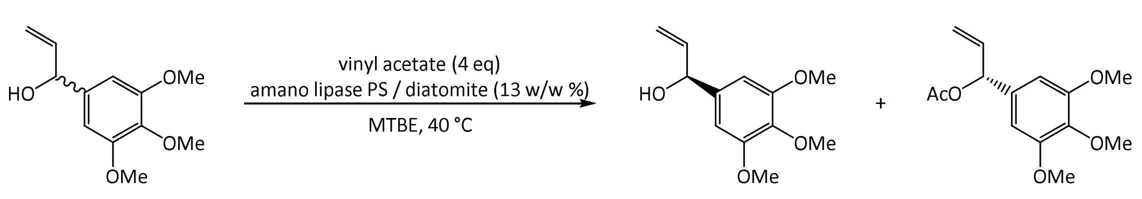

rac 7
Chemical Formula: $C_{12}H_{16}O_4$
Molecular Weight: 224,25

7
Chemical Formula: $C_{12}H_{16}O_4$
Molecular Weight: 224,25

8
Chemical Formula: $C_{14}H_{18}O_5$
Molecular Weight: 266,29

Rac 7 (28.3 g, 126.2 mmol, 1 equiv.) was dissolved in MTBE (690 mL, 0.18 M) and vinyl acetate (40.5 mL, 504.8 mmol, 4 equiv.). The solution was kept at 40.5 °C and Amano lipase PS on diatomite (3.68 g, 13 w/w %) was added. Reaction progress was monitored by chiral HPLC. After 76 h at this temperature the enantiomeric separation was complete, and the mixture was filtered through Celite 545. The solvent was removed in *vacuo* and the compounds were separated by column chromatography (MPLC, 2 x 90 g silica in sequence, 50 mL/min flow rate, 12 % EtOAc for 50 min, then 12-100 % in 60 min).

(S)-1-(3,4,5-Trimethoxyphenyl)prop-2-en-1-ol (7):

Yield: 11.17 g (39 %, >99.9 % ee)

Appearance: slightly yellow oil

TLC: Rf(PE/EtOAc = 0/1) = 0.28

$[\alpha]_D^{20}$ = +8.74 (MeOH; c 1.000)

GC-MS (EI, 70 eV, Method A): 224 (M^+, 81), 193 (38), 181 (25), 169 (100), 154 (24), 151 (24), 149 (25), 139 (19), 138 (52), 123 (18), 121 (31)

^{1}H-NMR (200 MHz, $CDCl_3$): δ 1.95 (bs, 1H, OH), 3.82 (s, 3H, OCH_3), 3.86 (s, 6H, $2xOCH_3$), 5.13 (d, J= 5.87 Hz, 1H, $C\underline{H}$-OH), 5.16-5.43 (m, 2H, $CH=C\underline{H_2}$), 5.93-6.13 (m, 1H, $CH-CH_2$), 6.59 (s, 2H, $C\underline{H}$-C-$C\underline{H}$)

^{13}C-NMR (50 MHz, $CDCl_3$): δ 56.01 (q 3' OCH_3 & 5' OCH_3), 60.76 (q, 4' OCH_3), 75.34 (d, $\underline{C}$H-OH), 103.08 (d, $\underline{C}$H-C-$\underline{C}$H), 115.19 (t, $CH=\underline{C}H_2$), 138.31 (s, C_q), 139.97 (d, $\underline{C}H=CH_2$), 153.24 (s, *m*-C_{aryl}). One C_q not visible.

(R)-1-(3,4,5-Trimethoxyphenyl)allyl acetate (8):

Yield: 15.17 g (45 %, 86 % ee)

Appearance: nearly colorless oil

TLC: Rf(PE/EtOAc = 1/2) = 0.27

$[\alpha]_D^{20}$ = +45.308 (MeOH; c 1.2531)

GC-MS (EI, 70 eV, Method A): 266 (M^+, 36), 224 (73), 207 (30), 206 (27), 191 (69), 177 (27), 176 (100), 163 (23), 161 (41), 149 (31), 148 (23), 133 (24), 121 (19), 106 (27), 105 (21), 103 (19)

^{1}H-NMR (200 MHz, $CDCl_3$): δ 2.11 (s, 3H, $COCH_3$), 3.82 (s, 3H, OCH_3), 3.85 (s, 6H, $2xOCH_3$), 5.19-5.36 (m, 2H, $CH=C\underline{H_2}$), 5.88-6.07 (m, 1H, $\underline{C}H=CH_2$), 6.18 (d, J=5.67 Hz, 1H, OCH), 6.56 (s, 2H, $C\underline{H}$-C-$C\underline{H}$)

^{13}C-NMR (50 MHz, $CDCl_3$): δ 21.3 (q, $CO\underline{C}H_3$), 56.1 (q, $2xOCH_3$), 60.8 (q, OCH_3), 76.1 (d, OCH), 104.3 (d, $\underline{C}$H-C-$\underline{C}$H), 116.8 (t, $CH=\underline{C}H_2$), 134.4 (s, *p*-C_{aryl}), 136.0 (d, $\underline{C}H=CH_2$), 137.8 (s, CH-$\underline{C}$(CH)-CH), 153.3 (s, *m*-C_{aryl}), 169.9 (s, C=O).

A.3 (1R)-Oxiran-2-yl(3,4,5-trimethoxyphenyl)methanol (9)

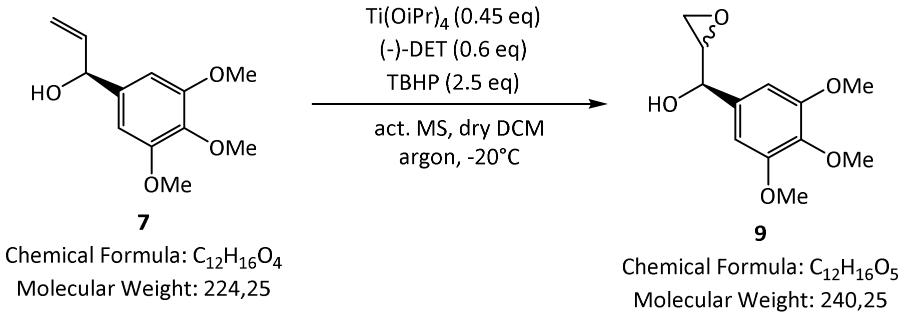

Dry DCM (150 mL), (-)-DET (2.789 g, 12.536 mmol, 0.6 equiv.) and **7** (5.085 g, 22.676 mmol, 1 equiv.) were (additionally) dried over activated MS overnight under argon atmosphere. (-)-DET was dissolved in dried DCM (1 mL) and cooled to -20 °C *via* a cryostat. Ti(OiPr)$_4$ (3.00 mL, 10.145 mmol, 0.45 equiv.) in dry DCM (70 mL, 0.14 M) was added and the reaction mixture was stirred for 15 min. Then TBHP (5.5 M in decane, 10.31 mL, 56.689 mmol, 2.5 equiv.) was added slowly. After 30 min the solution of **7** was added and the resulting mixture was stirred for 70 h at -20 °C. Reaction progress was monitored by TLC. When the reaction was finished, a solution of sodium sulfite (20 g in 100 mL water) was added as well as 1000 mL DCM and 500 mL water. The aqueous layer was extracted with DCM (4 x 500 mL) and the combined organic layers were dried over Na_2SO_4 and filtered. The solvent was removed in *vacuo* and the compound was purified *via* column chromatography (MPLC, product rotated on BULK Isolute Sorbent, 90 g silica, 50 mL/min flow rate, 8 % EtOAc for 15 min, then 8 -50 % EtOAc in 25 min, then 50-100 % EtOAc in 10 min, then 100 % for 10 min).

Yield: 4.431 g (81 %)

Appearance: orange oil

TLC: Rf(PE/EtOAc = 0/1) = 0.08

$[\alpha]_D^{20}$ = -13.748 (MeOH; c 1.3092)

^{1}H-NMR (200 MHz, $CDCl_3$): δ 2.36 (d, J=2.16 Hz, 1H, OH), 2.77 (dd, J=4.89 & 4.10 Hz, 1H, CH_2O), 2.93 (dd, J=5.09 & 2.74 Hz, 1H, CH_2O), 3.16-3.24 (m, 1H, CH_2-C<u>H</u>-O), 3.82 (s, 3H, OCH_3), 3.85 (s, 6H, 2xOCH_3), 4.77-4.83 (m, 1H, CH-<u>CH</u>-O), 6.60 (s, 2H, C<u>H</u>-C-C<u>H</u>)

^{13}C-NMR (50 MHz, $CDCl_3$): δ 43.7 (t, CH_2), 55.0 (d, <u>CH</u>-O-CH_2), 56.1 (q, 2xOCH_3), 60.8 (q, OCH_3), 71.1 (d, <u>CH</u>-OH), 103.2 (d, <u>C</u>H-C-<u>C</u>H), 135.2 (s, C_q), 153.4 (s, 2C, *m*-C_{aryl}). One C_q not visible.

A.4 2-((R)-(Prop-2-yn-1-yloxy)(3,4,5-trimethoxyphenyl)methyl)oxiran (10)

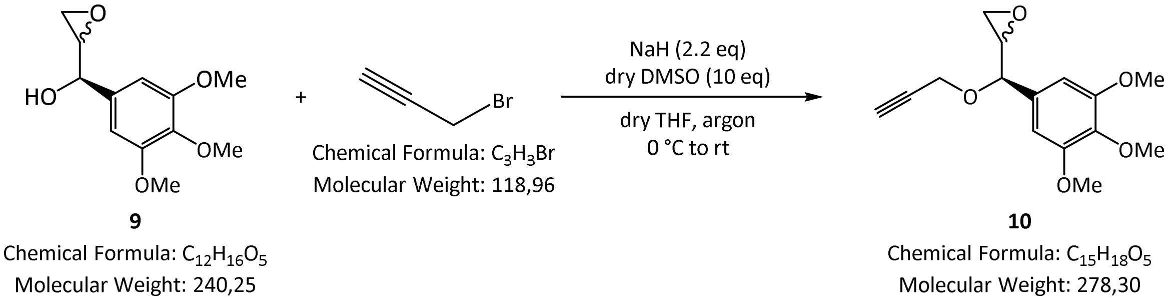

NaH (60 % dispersion in mineral oil, 2.2 equiv.) was dissolved in dry THF (1M with respect to NaH) under argon atmosphere and cooled with an ice bath. Dry DMSO (10 equiv.) was added. To this suspension substrate **9** (4.431 g, 18.443 mmol) added slowly as a solution in dry THF (0.4 M with respect to **9**). After stirring for 15 min a solution of 3-bromopropyne in THF was added slowly, usually followed by additional THF to dissolve the formed slurry. The ice bath was then removed, and the reaction mixture was stirred for 48 h. Progress of the reaction was monitored by TLC until complete conversion was indicated. The solution was cooled to 0 °C again and HCl (1M, 1 equiv.) was added drop wise. Most of the THF was then removed *in vacuo* and water was added. The aqueous layer was extracted with 4 x Et_2O, the combined organic layers were washed with brine, dried over Na_2SO_4, filtered, and the solvent was removed. The crude product was purified *via* column chromatography (MPLC, 90 g silica, 50 mL/min flow rate, 3 % EtOAc for 10 min, then 3-30 % EtOAc in 20 min, then 30-100 % EtOAc in 20 min, then 100 % EtOAc for 10 min).

Yield: 4.211 g, (82 %)

Appearance: slightly yellow oil

TLC: Rf (PE/EtOAc = 4/1) = 0.19

$[\alpha]_D^{20}$ = -118.8 (MeOH; c 1.0163)

GC-MS (EI, 70 eV, Method A): 9.97 min; Main fragments (relative intensity): 278 (M^+, 57), 248 (56), 235 (93), 209 (96), 196 (70), 195 (44), 193 (17), 192 (16), 181 (65), 179 (22), 178 (100), 176 (33), 168 (16), 166 (21), 163 (31), 161 (17), 156 (32), 151 (27), 135 (18), 121 (17)

^{1}H-NMR (200 MHz, $CDCl_3$): δ 2.46 (t, J=2.35 Hz, 1H, CH≡C-CH_2), 2.76 (dd, J= 5.19 & 2.54, 1H, CH-CH_2-O), 2.84 (dd, J= 5.19 & 3.91, 1H, CH-CH_2-O), 3.12-3.20 (m, 1H, CH-CH_2-O), 3.85 (s, 3H, OCH_3), 3.88 (s, 6H, 2xOCH_3), 4.01 (dd, J=15.66 & 2.35 Hz, 1H, C-CH_2-O), 4.24 (dd, J=15.66 & 2.34 Hz, 1H, C-CH_2-O), 4.43 (d, J=4.70 Hz, 1H, CH-CH-O), 6.60 (s, 2H, CH-C-CH).

^{13}C-NMR (50 MHz, $CDCl_3$): δ 45.5 (t, CH-CH_2-O), 54.0 (d, CH-CH_2-O), 56.1 (t, C-CH_2-O), 56.1 (q, 2xOCH_3), 60.8 (q, OCH_3), 74.9 (d, C≡CH), 79.2 (s, C≡CH), 79.7 (d, CH-CH-O), 104.2 (d, CH-C-CH), 132.7 (s, *p*-C_{aryl}), 138.0 (s, CH-C-CH), 153.4 (s, *m*-C_{aryl}).

A.5 ((2S,3R)-4-Methylene-2-(3,4,5-trimethoxyphenyl)tetrahydrofuran-3-yl)methanol (11)

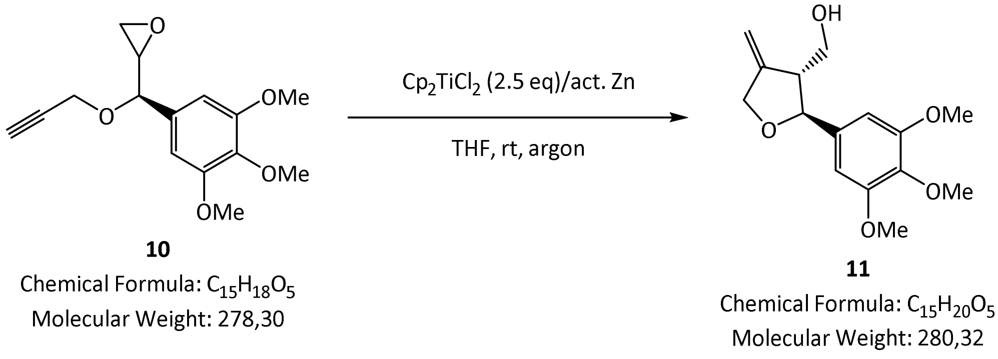

A flask was charged with act. Zn (2.306 g, 35.264 mmol, 7 equiv.) and Cp_2TiCl_2 (3.135 g, 12.594 mmol, 2.5 equiv.) under argon; deoxygenated THF (60 mL, distilled from Na/benzophenone) was added. After 1 h of stirring at rt, the Zn was allowed to settle for 5 min and the solution (without the Zn) was transferred to a solution of **10** (1.402 g, 5.038 mmol, 1 equiv.) in deoxygenated THF (40 mL) over a period of 25 min. Stirring was continued for 70 min at rt and reaction progress was monitored by TLC. When the reaction was completed, diluted sulfuric acid (10 % in water, 30 mL) was added and the major amount of THF was evaporated. Water was added to the crude product and the aqueous layer was extracted with Et_2O (4 x 200 mL). The combined organic layers were washed with satd. $NaHCO_3$ solution, brine, dried over Na_2SO_4, filtered and the solvent was removed in *vacuo*. The crude product was purified via column chromatography (MPLC, 90 g silica, 50 mL/min flow rate, 10-50 % EtOAc for 20 min, then 50-100 % in 15 min, then 100 % for 15 min).

Yield: 1.412 g, (61 %)

Appearance: yellow oil

TLC: Rf(PE/EtOAc = 2/1) = 0.07

$[\alpha]_D^{20}$ = +6.2 (MeOH; c 1.0791)

HRMS (ESI$^+$): exact mass calculated for $C_{15}H_{20}O_5$: 303.1203. Found: 303.1203. $[M+Na]^+$, Δ = 0.00 ppm

GC-MS (EI, 70 eV, Method A): 280 (17), 197 (12), 196 (11), 195 (11), 182 (37), 181 (100), 169 (57), 154 (31), 139 (13), 138 (45), 125 (13), 115 (12), 110 (10)

^{1}H-NMR (200 MHz, $CDCl_3$): δ 1.62 (bs, 1H, OH), 2.69-85 (m, 1H, H3), 3.82 (s, 3H, OCH_3), 3.85 (s, 8H, 2x OCH_3 & CH_2O), 4.35-4.69 (m, 2H, H5), 4.78 (d, J=7.44 Hz, 1H, H2), 5.02-5.16 (m, 2H, CH_2), 6.63 (s, 2H, H2' & H6').

^{13}C-NMR (50 MHz, $CDCl_3$): δ 54.0 (d, C4), 56.0 (q, 2xOCH_3), 60.7 (q, OCH_3), 61.9 (t, CH_2O), 71.4 (d, C3), 83.4 (d, C2), 103.5 (d, C2' & C6'), 105.0 (t, CH_2), 136.8 (s, C_q), 148.5 (d, C3), 153.5 (s, C3' & C5'). One C_q not visible

A.6 *tert*-Butyldimethyl(((2S,3R)-4-methylene-2-(3,4,5-trimethoxyphenyl)tetrahydrofuran-3-yl)methoxy)silane (12)

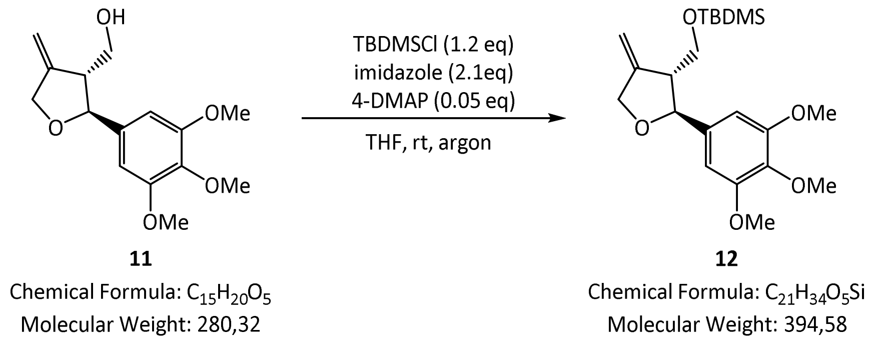

Substrate **11** (1.014 g, 3.617 mmol, 1 equiv.), imidazole (0.518 g, 7.596 mmol, 2.1 equiv.) and 4-DMAP (23.1 mg, 0.181 mmol, 0.05 equiv.) were dissolved in DMF (21 mL, 0.17 M) under argon. TBDMSCl (3M in THF, 1.45 mL, 4.341 mmol, 1.2eq) was added to the solution and the mixture was stirred for 16 h at rt. Reaction progress was monitored by TLC. When the reaction was finished, Et_2O (50 mL) and satd. NH_4Cl solution (20 mL) were added and the aqueous phase was extracted with Et_2O (4 x 30 mL). The combined organic layers were washed with satd. $NaHCO_3$ solution (10 mL) and brine (10 mL), dried over Na_2SO_4 and filtered. The solvent was evaporated, and the crude product used without purification in the next step.

A.7 *tert*-Butyl(((2S,3R,4R)-4-(3,4-dimethoxybenzyl)-2-(3,4,5-trimethoxyphenyl) tetrahydrofuran-3-yl)methoxy)dimethylsilane (I)

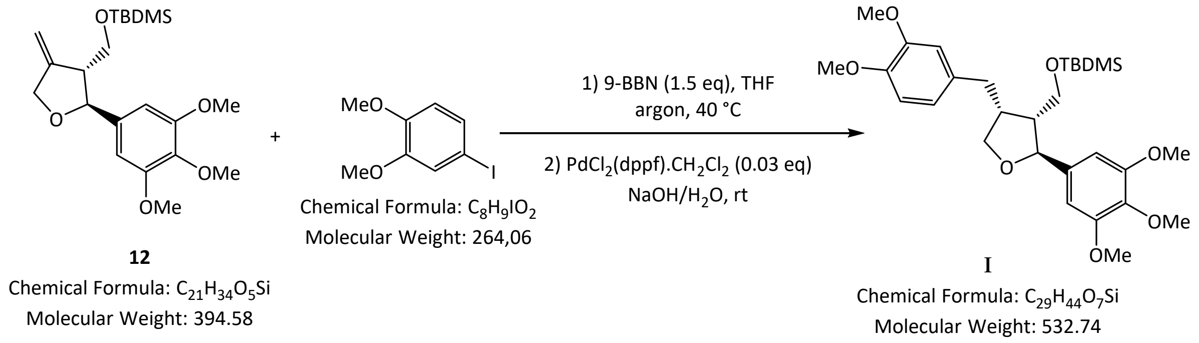

A flask was charged with **12** (3.617 mmol, 1 equiv.) under argon and 9-BBN (0.5 M in THF, 10.85 mL, 5.426 mmol, 1.5 equiv.) was added. The resulting mixture was stirred for 22.5 h at 40 °C. On the next day, it was allowed to warm to rt and aqueous NaOH solution (1 M, 10 mL) was added. Stirring was continued for another 15 min and 4-iodoveratrole (1.248 g, 4.792 mmol, 1.3 equiv.) and $PdCl_2(dppf).CH_2Cl_2$ (88.5 mg, 0.109 mmol, 0.03 equiv.) were added. The mixture became biphasic and was stirred for another 24 h at rt, then Et_2O (100 mL) and brine (25 mL) were added. Reaction progress was monitored by TLC. The layers were separated, and the aqueous phase was extracted with Et_2O (4 x 30 mL). The combined organic layers were dried over Na_2SO_4, filtered and the solvent was evaporated. The crude product was used without further purification in the next step.

A.8 ((2S,3R,4S)-4-(3,4-Dimethoxyphenyl)- 2-(3,4,5-trimethoxyphenyl)tetrahydro-3-yl) methanol (13)

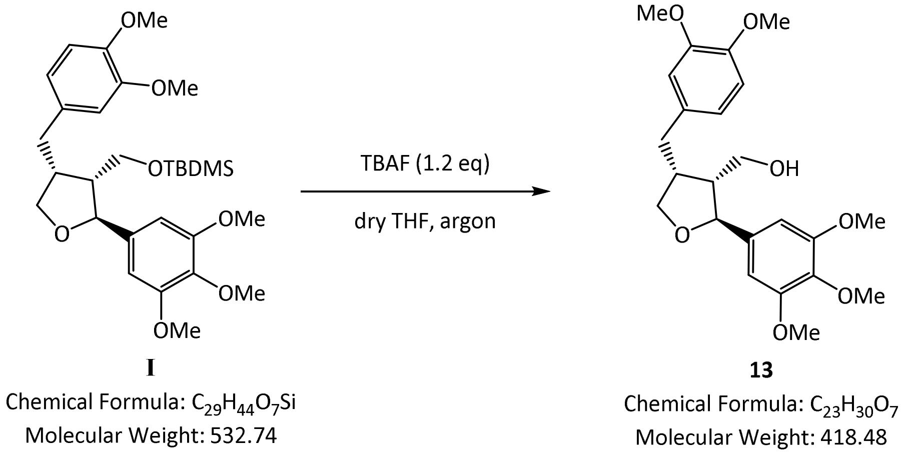

A flask was charged with **I** (3.617 mmol, 1 equiv.) under argon and TBAF (1 M in THF, 4.34 mL, 4.341 mmol) was added. The reaction was stirred for 20 h at rt. Reaction progress was monitored by TLC. When the reaction was finished, Et_2O (100 mL) and brine (25 mL) were added and the aqueous phase was extracted with Et_2O (4 x 30 mL) and EtOAc (2 x 35 mL). The combined organic layers were dried over Na_2SO_4, filtered and the solvent was evaporated. The compound was purified *via* column chromatography (MPLC, 90 g silica, 40 mL/min flow rate, 30 EtOAc for 5 min, then 30-100 % EtOAc in 45 min).

Yield: 0.631 g (42 % over 3 steps)

Appearance: yellow oil

TLC: Rf(PE/EtOAc = 1/1) = 0.27

Specific rotation: $[\alpha]_D^{20}$ = +7.1 (MeOH; c 2.143)

HRMS (ESI$^+$): exact mass calculated for $C_{23}H_3O_7$: 441.1884. Found: 441.1903. $[M+Na]^+$, Δ = 4.31 ppm

^{1}H-NMR (200 MHz, $CDCl_3$): δ 1.68 (bs, 1H, OH), 2.32-2.82 (m, 3H, H3 & H4 & CH_2), 2.92 (dd, J=12.82 & 4.60 Hz, 1H, CH_2), 3.83 (s, 3H, OCH_3), 3.85 (s, 15H, 4xOCH_3, CH_2O, H5), 4.07 (dd, J=8.41 & 6.45 Hz, 1H, H5), 4.84 (d, J=6.06 Hz, 1H, H2), 6.56 (s, 2H, H2' & H6'), 6.67-6-83 (m, 3H, H2" & H5" & H6").

^{13}C-NMR (50 MHz, $CDCl_3$): δ 33.1 (t, CH_2), 42.2 (d, C4), 52.4 (d, C3), 55.8 (q, 2xOCH_3), 56.1 (q, 2xOCH_3), 60.8 (q, OCH_3), 60.9 (t, CH_2OH), 73.0 (t, C5), 82.9 (d, C2), 102.5 (d, C2' & C6'), 111.2 (d, CH), 111.8 (d, CH), 120.4 (d, C6"), 132.9 (s, C1"), 137.05 (s, C_q), 138.7 (s, C_q), 147.4 (s, C4"), 148.9 (s, C3"), 153.2 (s, C3' & C5').

A.9 (Z)-((2S,3R,4S)-4-(3,4-Dimethoxyphenyl)- 2-(3,4,5-trimethoxyphenyl)tetrahydro-furan -tetrahydrofuran-3-yl)methyl 2-methylbut-2-enoate (1) [9]

Method 1:

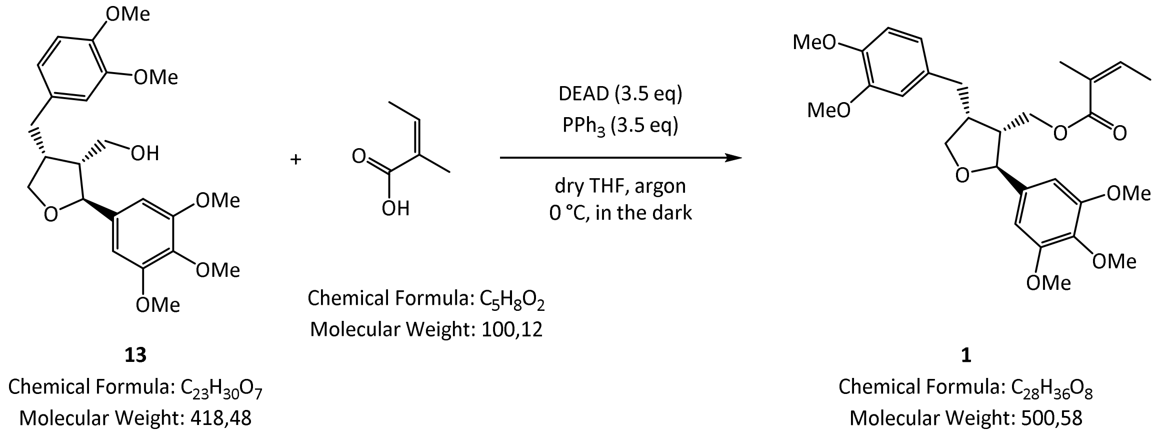

The substrate **13** (152.6 mg, 0.365 mmol), angelic acid (1.5 equiv.) and PPh_3 (3.5 equiv.) were charged under argon, cooled with an ice bath and dissolved in THF (0.13 M). DEAD (3.5 equiv.) was added slowly and the reaction was stirred for 18.5 h in the dark while being allowed to warm to rt. Reaction progress was monitored by TLC. When the reaction was finished, brine was added, the layers separated, and the aqueous phase was extracted with 3 x Et_2O. The combined organic layers were dried over Na_2SO_4, filtered and the solvent was removed via evaporation. The crude product was purified *via* column chromatography (MPLC, 40 g silica, 50 mL/min flow rate, 10-22 % EtOAc in 10 min, then 22 % EtOAc for 10 min, then 22-65 % EtOAc in 40 min.

Method 2:

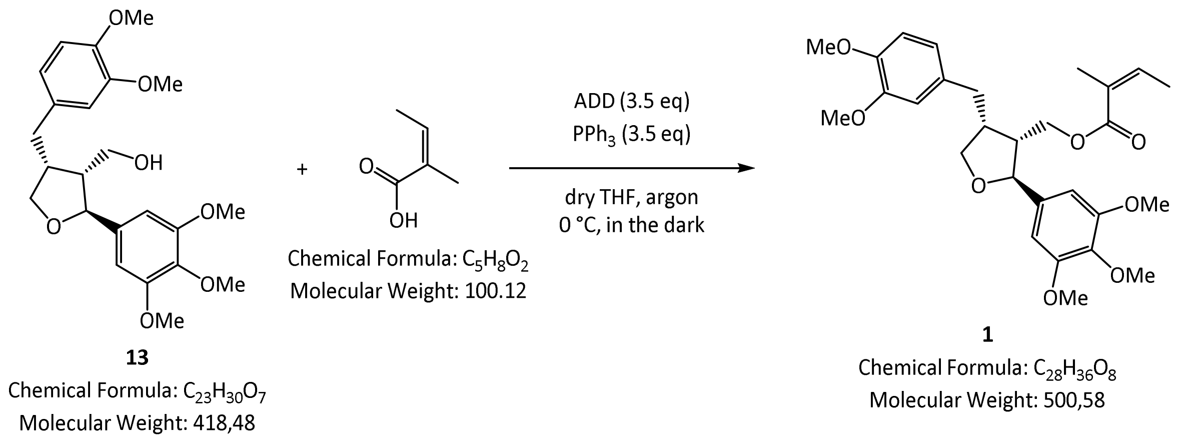

Prepared according to the above procedure. Modification: Instead of using DEAD, ADD was used.

Yield: Method 1: 30.2 mg (34 %), Method 2: 134.2 mg (74 %)

Appearance: slightly yellow viscous oil

TLC: Rf(PE/EtOAc = 2/1) = 0.25

$[\alpha]_D^{20}$ = +19.7 (MeOH; c 1.5415). According to literature: $[\alpha]_D^{20}$ = +20.86 (MeOH; 0.302)

HRMS (ESI⁺): exact mass calculated for $C_{28}H_{36}O_8$: 523.2302. Found: 523.2311. $[M+Na]^+$, Δ = 1.72 ppm

GC-MS (EI, 70 eV, Method B): 500 (M^+, 12), 249 (24), 203 (13), 196 (11), 195 (61), 190 (11), 189 (15), 181 (23), 178 (11), 177 (14), 152 (15), 151 (100), 107 (14)

^{1}H-NMR (200 MHz, CDCl₃): δ 1.83-1.90 (m, 3H, α-CH_3), 1.95-2.04 (m, 3H, β-CH_3), 2.47-2.81 (m, 3H, H3 & H4 & CH_2), 2.88 (dd, 1H, CH_2), 3.77 (dd, J= 8.70 & 6.26 Hz, 1H, H5), 3.81 (s, 3H, OCH_3), 3.84 (s, 6H, 2xOCH_3), 3.85 (s, 6H, 2xOCH_3), 4.07 (dd, J=8.70 & 6.07 Hz, 1H, H5), 4.29 (dd, J=11.35 & 7.14, 1H, CH_2O), 4.43 (dd, J=11.35 & 6.56 Hz, 1H, CH_2O), 4.83 (d, J=5.87, 1H, H2), 6.02-6.17 (m, 1H, β-CH), 6.54 (s, 2H, H2' & H6'), 6.64-6.83 (m, 3H, H2'' & H5'' & H6'').

^{13}C-NMR (50 MHz, CDCl₃): δ 15.8 (q, β-CH_3), 20.5 (q, α-CH_3), 33.12 (t, CH_2), 42.56 (d, C4), 49.2 (d, C3), 55.8 (q, OCH_3), 55.9 (q, OCH_3), 56.1 (q, C3' OCH_3 & C5' OCH_3), 60.8 (q, C4' OCH_3), 62.2 (t, CH_2O), 72.8 (t, C5), 83.1 (d, C2), 102.5 (d, C2' & C6'), 111.3 (d, C2''), 111.8 (d, C5''), 120.4 (d, C6''), 127.2 (s, α-C), 132.7 (s, C1''), 138.2 (s, C_q), 139.0 (d, β-CH), 147.5 (s, C4''), 148.9 (s, C3''), 153.3 (s, C3' & C5'), 167.6 (s, C=O). One C_q not visible.

B. Synthesis of leoligin analogs

B.1 (E)-((2S,3R,4R)-4 -(3,4-Dimethoxybenzyl)-2-(3,4-dimethoxyphenyl)tetrahydrofuran -3-yl)methyl 2-methylbut-2-enoate (35)

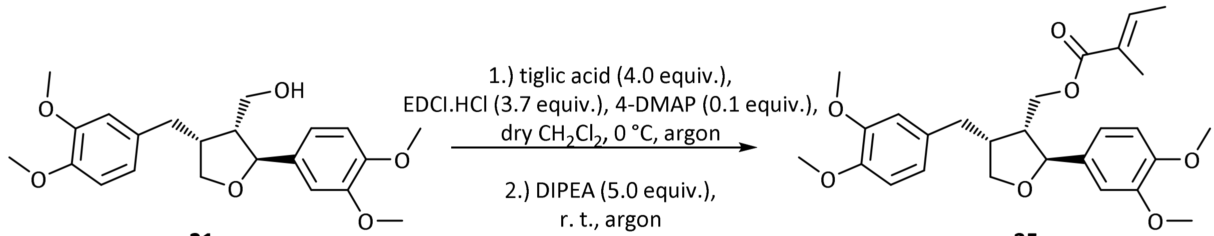

21 **35**

Chemical Formula: $C_{22}H_{28}O_6$
Molecular Weight: 388.45 gmol⁻¹

Chemical Formula: $C_{27}H_{34}O_7$
Molecular Weight: 470.55 g mol⁻¹

A reaction vessel was charged with a stirring bar, tiglic acid (36.0 mg, 0.360 mmol, 4.0 equiv.) and 4-DMAP (1.1 mg, 9.0 µmol, 0.1 equiv.), and then evacuated and back-filled with argon using standard Schlenk technique. Dry CH_2CL_2 (1.0 mL) was then added via syringe and the solution was cooled to 0 °C in an ice bath. The vessel was briefly opened, EDCl.HCl (63.8 mg, 0.33 mmol, 3.7 equiv.) added in one go and the mixture was stirred for 3 h at 0 °C. Meanwhile, a second vessel was charged with a stirring bar and starting material **12** (35.0 mg, 0.090 mmol, 1.00 equiv.), evacuated and back-filled with argon (3 x), and DIPEA (78 µL, 0.45 mmol, 5.0 equiv.) was added via syringe. After 3 h, the solution containing the activated carboxylic acid was transferred to the second vial via syringe and stirred for 16 h at room temperature. The reaction solution was used directly for flash chromatography (9 g silica, flow rate 20 mL / min, EtOAc/ LP, 10:90 to 50:50 in 30 min).

Yield: 40.3 mg (95 %)

Appearance: colorless oil

TLC: Rf (EtOAc/ LP = 1/1) = 0.57

$[\alpha]_D^{20}$ = +17.5 (MeOH; c 12.48).

LC-HRMS (ESI): exact mass calculated for $[M+Na]^+$: 523.2302. Found: 493.2204. Δ = 1.42 ppm

(log *P*) calc: 5.38 ± 0.48

GC-MS (EI, 70 eV, Method D): 26.18 min; 470.2 (M^+, 2), 219.1 (30), 189.1 (16), 177.1 (16), 166.1 (15), 165.1 (90), 151.1 (100), 107.1 (16).

^{1}H NMR (200 MHz, CDCl$_3$): δ 1.73 – 1.84 (m, 6H, 3 x H4''', 3 x H5'''), 2.48 – 2.97 (m, 4H, H3, H4, C4-CH$_2$), 3.77 (dd, ^2J = 8.6 Hz, ^3J = 6.2 Hz, 1H, H5), 3.86 (s, 3H, Ar-OCH$_3$), 3.87 (s, 6H, Ar-OCH$_3$), 3.88 (s, 3H, Ar-OCH$_3$), 4.09 (dd, ^2J = 8.6 Hz, ^3J = 6.2 Hz, 1H, H5), 4.26 (dd, ^2J = 11.3 Hz, ^3J = 7.0 Hz, 1H, C3-CH), 4.43 (dd, ^2J = 11.3 Hz, ^3J = 6.6 Hz, 1H, C3-CH), 4.83 (d, ^3J = 6.3 Hz, 1H, H2), 6.67 – 6.88 (m, 7H, 6 x Ar-H, H3''').

^{13}C NMR (50 MHz, CDCl$_3$): δ 12.1 (q, C5'''), 14.5 (q, C4'''), 33.4 (t, C4-C), 42.8 (d, C4), 49.3 (d, C3), 55.98 (q, Ar-OCH$_3$), 56.01 (q, Ar-OCH$_3$), 56.03 (q, Ar-OCH$_3$), 56.1 (q, Ar-OCH$_3$), 62.8 (t, C3-C), 72.9 (t, C5), 83.3 (d, C2), 109.1 (d, C2'), 111.1 (d, C5'), 111.4 (d, C5''*), 112.0 (d, C2''*), 118.3 (d, C6'), 120.6 (d, C6''), 128.4 (s, C2'''), 132.8 (s, C1''), 135.1 (s, C1'), 137.9 (d, C3'''), 147.6 (s, C4''), 148.6 (s, C4'), 149.1 (s, C3''), 149.2 (s, C3'), 168.0 (s, C1''').

B.2 ((2*S*,3*R*,4*R*)- 4-(3,4-Dimethoxybenzyl)-2-(3,4-dimethoxyphenyl)tetrahydrofuran-3-yl) methyl 3-methylbut-2-enoate (36)

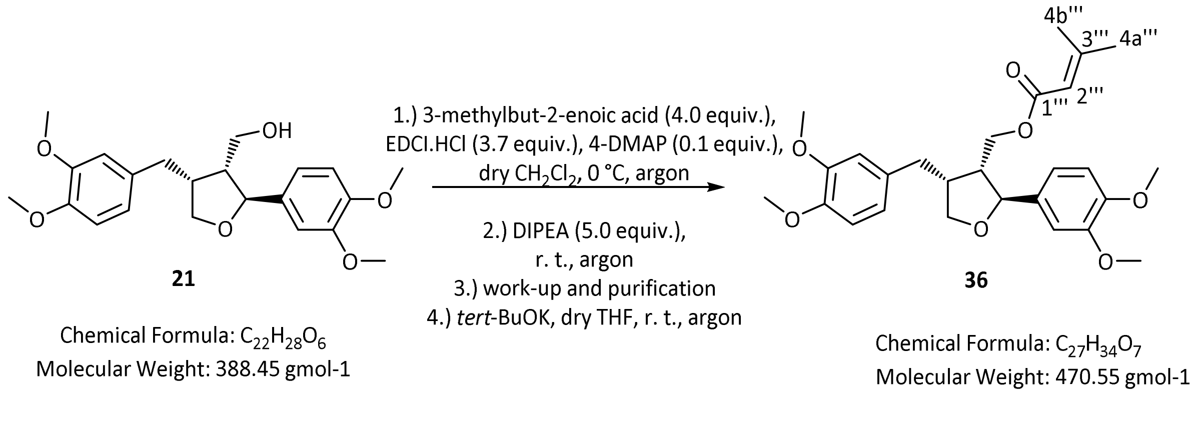

A reaction vessel was charged with a stirring bar, 3-methylbut-2-enoic acid (36.0 mg, 0.360 mmol, 4.0 equiv.) and 4-DMAP (1.1 mg, 9.0 µmol, 0.1 equiv.), and then evacuated and back-filled with argon using standard Schlenk technique (1 x). Dry CH$_2$Cl$_2$ (1.0 mL) was then added via syringe and the solution was cooled to 0 °C in an ice bath. The vessel was briefly opened, EDCI.HCl (63.8 mg, 0.333 mmol, 3.7 equiv.) added in one go and the mixture was stirred for 3 h at 0 °C. Meanwhile, a second vessel was charged with a stirring bar and starting

material **12** (35.0 mg, 0.090 mmol, 1.00 equiv.), evacuated and back-filled with argon (3 x), and DIPEA (78 µL, 0.45 mmol, 5.0 equiv.) was added via syringe. After 3 h, the solution containing the activated carboxylic acid was transferred to the second vial via syringe and stirred for 16 h at room temperature. The reaction solution was used directly for flash column chromatography (9 g silica, flow rate 20 mL / min, EtOAc / LP, 10 : 90 to 22 : 78 in 9 min, then 22 : 78 isocratically for 6 min, then to 62 : 38 in 30 min) to give a mixture of the targeted compound **36**, as well as β-γ double bond isomerization compound **36'** (approximate ratio 3 : 1, by NMR, 34.4 mg).[1] Thus, a new reaction vessel was charged with a stirring bar and part of the so obtained material (24.7 mg, 0.052 mmol), evacuated and back-filled with argon. To this was then added *tert*-BuOK (2.9 mg, 0.026 mmol) in dry THF (1.0 mL) via syringe and the solution stirred at room temperature for 18 h. THF (1.0 mL) was added, followed by Et_2O (15 mL) and a solution of $KHSO_4$ (0.029 mmol, 3.9 mg) in brine (2 mL). Water (1.5 mL) was added to dissolve the salts, the layers were separated, the aqueous phase was re-extracted with Et_2O (2 x 10 mL), the combined organic phases were dried with Na_2SO_4, filtered and the solvents were evaporated. Finally, flash column chromatography (9 g silica, flow rate 20 mL / min, EtOAc / LP, 10 : 90 to 50 : 50 in 30 min).

Yield: 17.0 mg, 40 % (with respect to the amount of starting material **21**), 56 % (with respect also to the amount of α−β and β−γ mixture applied for de-isomerization), respectively

Appearance: nearly colorless oil

***R*f (silica)**: 0.50 (EtOAc / LP, 1 : 1)

$[\alpha]_D^{20}$: +29.2 (c 1.63, MeOH)

LC-HRMS (ESI): calculated for $M+Na^+$: 493.2197, found: 493.2201, Δ: 0.81 ppm

(log *P*)calc: 5.38 ± 0.48

GC-MS (EI, 70 Ev, Method D): 25.73 min; 470.2 (M^+, 2), 219.1 (29), 189.1 (17), 177.1 (16), 166.1 (15), 165.0 (89), 152.1 (15), 151.1 (100), 107.0 (18).

^{1}H NMR (200 MHz, $CDCl_3$): δ 1.90 (d, ^4J = 1.1 Hz, 3H, H4b'''), 2.17 (d, ^4J = 1.1 Hz, 3H, H4a'''), 2.47 – 2.84 (m, 3H, H3, H4, C4-CH), 2.89 (dd, ^2J = 12.6 Hz, ^3J = 4.3 Hz, 1H, C4-CH), 3.75 (dd, ^2J = 8.6 Hz, ^3J = 6.4 Hz, 1H, H5), 3.86 (s, 3H, Ar-OCH_3), 3.87 (s, 6H, Ar-OCH_3), 3.88 (s, 3H, Ar-OCH_3), 4.07 (d, ^2J = 8.5 Hz, ^3J = 6.2 Hz, 1H, H5), 4.21 (dd, ^2J = 11.3 Hz, ^3J = 6.9 Hz, 1H, C3-CH), 4.37 (dd, ^2J = 11.3 Hz, ^3J = 7.1 Hz, 1H, C3-CH), 4.81 (d, ^3J = 6.3 Hz, 1H, H2), 5.62 – 5.68 (m, 1H, H2'''), 6.67 – 6.91 (m, 6H, Ar-H).

^{13}C NMR (50 MHz, $CDCl_3$): δ 20.4 (q, C4b'''), 27.6 (q, C4a'''), 33.3 (t, C4-C), 42.7 (d, C4), 49.3 (d, C3), 55.97 (q, Ar-OCH_3), 55.99 (q, Ar-OCH_3), 56.02 (q, Ar-OCH_3), 56.1 (q, Ar-OCH_3), 61.8 (t, C3-C), 72.9 (t, C5), 83.1 (d, C2), 109.0 (d, C2'), 111.1 (d, C5'), 111.5 (d, C5''*), 112.1 (d, C2''*), 115.7 (d, C2'''), 118.2 (d, C6'), 120.6 (d, C6''), 132.9 (s, C1''), 135.2 (s, C1'), 147.6 (s, C4''), 148.5 (s, C4'), 149.1 (s, C3''), 149.1 (s, C3'), 157.7 (s, C3'''), 166.6 (s, C1''').

B.3 ((2*S*,3*R*,4*R*)- 4-(3,4-Dimethoxybenzyl)-2-(3,4-dimethoxyphenyl)tetrahydrofuran-3-yl)methyl benzoate (37)

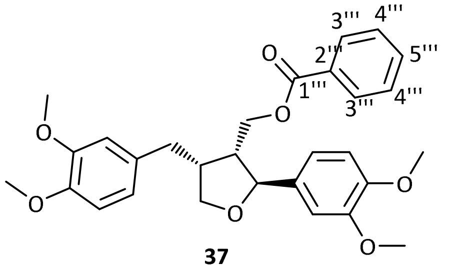

37

Chemical Formula: $C_{29}H_{32}O_7$
Molecular Weight: 492.56 $gmol^{-1}$

***Preparation**:* analogous to **36**, using starting material **21** (35.0 mg, 0.090 mmol, 1.00 equiv.) and benzoic acid (44.0 mg, 0.360 mmol, 4.0 equiv.).The reaction solution was used directly for flash column chromatography (9 g silica, flow rate 20 mL / min, EtOAc / LP, 10 : 90 to 22 : 78 in 9 min, then 22 : 78 isocratically for 6 min, then to 62 : 38 in 30 min).

Yield: 40.4 mg, (91 %)

Appearance: colorless oil

R_f (silica): 0.54 (EtOAc / LP, 1 : 1)

$[\alpha]_D^{20}$: +20.2 (c 2.04, MeOH)

LC-HRMS (ESI): calculated for $M+Na^+$: 515.2040, found: 515.2050, Δ: 1.94 ppm

(log *P*)$_{calc}$: 5.70 ± 0.44

GC-MS (EI, 70 eV, Method D): 46.13 min; 492.2 (M^+, 3), 219.1 (24), 207.0 (24), 189.1 (16), 177.1 (15), 165.1 (72), 151.1 (94), 107.1 (18), 106.1 (15), 105.0 (100).

^{1}H NMR (200 MHz, CDCl$_3$): δ 2.55 – 3.02 (m, 4H, H3, H4, C4-CH$_2$), 3.76 – 3.83 (m, 1H, H5), 3.84 (s, 3H, Ar-OCH$_3$), 3.85 (s, 6H, Ar-OCH$_3$), 3.86 (s, 3H, Ar-OCH$_3$), 4.13 (dd, ^2J = 8.6 Hz, ^3J = 6.1 Hz, 1H, H5), 4.46 (dd, ^2J = 11.2 Hz, ^3J = 7.1 Hz, 1H, C3-CH), 4.63 (dd, ^2J = 11.3 Hz, ^3J = 6.3 Hz, 1H, C3-CH), 4.91 (d, ^3J = 6.2 Hz, 1H, H2), 6.66 – 6.95 (m, 6H, 3 x Ar'-H, 3 x Ar''-H), 7.42 (t, ^3J = 7.4 Hz, 2H, H4'''), 7.50 – 7.62 (m, 1H, H5'''), 7.89 – 7.97 (m, 2H, H3''').

^{13}C NMR (50 MHz, CDCl$_3$): δ 33.5 (t, C4-C̲), 42.8 (d, C4), 49.3 (d, C3), 55.9 (q, Ar-OCH$_3$), 55.99 (q, Ar-OCH$_3$), 56.02 (q, Ar-OCH$_3$), 56.1 (q, Ar-OCH$_3$), 63.4 (t, C3-C̲), 73.0 (t, C5), 83.5 (d, C2), 109.1 (d, C2'), 111.2 (d, C5'), 111.4 (d, C5''*), 112.0 (d, C2''*), 118.3 (d, C6'), 120.6 (d, C6''), 128.5 (d, C4'''), 129.7 (d, C3'''), 130.0 (s, C2'''), 132.6 (s, C1''), 133.3 (d, C5'''), 135.0 (s, C1'), 147.7 (s, C4''), 148.6 (s, C4'), 149.1 (s, C3''), 149.2 (s, C3'), 166.5 (s, C1''').

B.4 ((2*S*,3*R*,4*R*)-4-(3,4-Dimethoxybenzyl)-2-(3,4-dimethoxyphenyl)tetrahydrofuran-3-yl)methyl 2-mehylbenzoate (38)

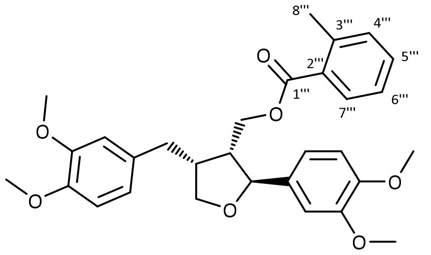

38

Chemical Formula: $C_{30}H_{34}O_7$
Molecular Weight:506.59 gmol-1

***Preparation**:* analogous to **36**, using starting material **21** (29.5 mg, 0.076 mmol, 1.0 equiv.), 2-methylbenzoic acid (41.4 mg, 0.304 mmol, 4.0 equiv), EDCI.HCl (53.9 mg, 0.281 mmol, 3.7 equiv.), 4-DMAP (0.9 mg, 7.6 µmol, 0.1 equiv.) and DIPEA (66 µL, 0.38 mmol, 5.0 equiv.). The reaction solution was used directly for flash column chromatography (9 g silica, flow rate 20 mL / min, EtOAc / LP, 7 : 93 to 50 : 50 in 30 min).

Yield: 28.9 mg, (75 %)

Appearance: colorless oil

R_f (silica): 0.44 (EtOAc / LP, 1 : 1)

$[\alpha]_D^{25}$: +19.1 (c 2.89, MeOH)

LC-HRMS (ESI): calculated for M+Na^+: 529.2197, found: 529.2234, Δ: 6.99 ppm

(log *P*)$_{calc}$: 6.16 ± 0.44

GC-MS (EI, 70 eV, Method E): 50.43 min; 506.3 (M^+, 4), 219.1 (31), 207.0 (35), 189.1 (18), 177.1 (16), 166.1 (15), 165.1 (79), 152.1 (15), 151.1 (100), 119.0 (88), 107.1 (15), 91.1 (58).

^{1}H NMR (200 MHz, $CDCl_3$): δ 2.53 – 2.90 (m, 3H, H3, H4, C4-CH), 2.60 (s, 3H, H8'''), 2.95 (dd, ^2J = 12.5 Hz, ^3J = 4.2 Hz, 1H, C4-CH), 3.72 – 3.82 (m, 1H, H5), 3.83 (s, 3H, Ar-OCH_3), 3.85 (s, 6H, Ar-OCH_3), 3.86 (s, 3H, Ar-OCH_3), 4.12 (dd, ^2J = 8.6 Hz, ^3J = 6.0 Hz, 1H, H5), 4.42 (dd, ^2J = 11.2 Hz, ^3J = 7.0 Hz, 1H, C3-CH), 4.60 (dd, ^2J = 11.3 Hz, ^3J = 6.6 Hz, 1H, C3-CH), 4.90 (d, ^3J = 6.3 Hz, 1H, H2), 6.66 – 6.94 (m, 6H, Ar-H), 7.15 – 7.27 (m, 2H, H4''', H6'''), 7.41 (td, ^3J = 7.5 Hz, ^4J = 1.3 Hz, 1H, H5'''), 7.76 (dd, ^3J = 7.6 Hz, ^4J = 1.3 Hz, 1H, H7''').

^{13}C NMR (50 MHz, $CDCl_3$): δ 21.9 (q, C8'''), 33.4 (t, C4-C), 42.8 (d, C4), 49.4 (d, C3), 55.91 (q, Ar-OCH_3), 55.94 (q, Ar-OCH_3), 55.98 (q, Ar-OCH_3), 56.02 (q, Ar-OCH_3), 63.1 (t, C3-C), 72.9 (t, C5), 83.3 (d, C2), 109.1 (d, C2'), 111.1 (d, C5'), 111.4 (d, C5''*), 112.0 (d, C2''*), 118.3 (d, C6'), 120.6 (d, C6''), 125.8 (d, C6'''), 129.2 (s, C2'''), 130.6 (d, C4'''), 131.9 (d, C7'''*), 132.3 (d, C5'''*), 132.6 (s, C1''), 135.0 (s, C1'), 140.6 (s, C3'''), 147.6 (s, C4''), 148.6 (s, C4'), 149.1 (s, C3''), 149.2 (s, C3'), 167.3 (s, C1''').

B.5 ((2*S*,3*R*,4*R*)-4-(3,4-Dimethoxybenzyl)-2-(3,4-dimethoxyphenyl)tetrahydrofuran-3-yl)methyl cyclopropanecarboxylate (39)

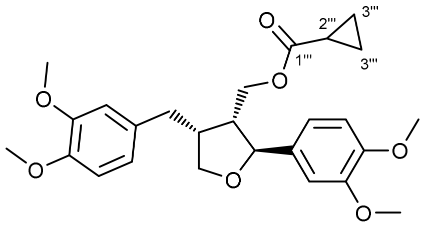

39

Chemical Formula: $C_{28}H_{32}O_7$
Molecular Weight: 456.53 gmol-1

Preparation: analogous to **36**, using starting material **21** (35.0 mg, 0.090 mmol, 1.00 equiv.), cyclopropanecarboxylic acid (17.8 mg, 0.207 mmol, 2.3 equiv.), EDCI.HCl (34.5 mg, 0.180 mmol, 2.0 equiv.), 4-DMAP (1.1 mg, 9.0 µmol, 0.1 equiv.) and DIPEA (39 µL, 0.23 mmol, 2.5 equiv.). The reaction solution was used directly for flash column chromatography (9 g silica, flow rate 20 mL / min, EtOAc / LP, 10 : 90 to 22 : 78 in 9 min, then 22 : 78 isocratically for 6 min, then to 62 : 38 in 30 min).

Yield: 37.4 mg, (91 %)

Appearance: colorless oil

***R*f (silica)**: 0.54 (EtOAc / LP, 1 : 1)

$[\alpha]_D^{20}$: +17.8 (c 1.89, MeOH)

LC-HRMS (ESI): calculated for M+Na$^+$: 479.2040, found: 479.2048, Δ: 1.67 ppm

(log *P*)calc: 4.05 ± 0.44

GC-MS (EI, 70 eV, Method C): 23.81 min; 456.2 (M$^+$, 5), 219.1 (27), 189.1 (16), 165.1 (67), 152.1 (15), 151.0 (100), 107.1 (16).

^{1}H NMR (200 MHz, CDCl$_3$): δ 0.80 – 1.02 (m, 4H, H3'''), 1.50 – 1.64 (m, 1H, H2'''), 2.46 – 2.94 (m, 4H, H3, H4, C4-CH$_2$), 3.75 (dd, ^2J = 8.6 Hz, ^3J = 6.3 Hz, 1H, H5), 3.86 (s, 3H, Ar-OCH$_3$), 3.87 (s, 6H, Ar-OCH$_3$), 3.89 (s, 3H, Ar-OCH$_3$), 4.08 (dd, ^2J = 8.6 Hz, ^3J = 6.3 Hz, 1H, H5), 4.19 (dd, ^2J = 11.2 Hz, ^3J = 7.1 Hz, 1H, C3-CH), 4.37 (dd, ^2J = 11.2 Hz, ^3J = 6.9 Hz, 1H, C3-CH), 4.81 (d, ^3J = 6.3 Hz, 1H, H2), 6.68 – 6.91 (m, 6H, Ar-H).

^{13}C NMR (50 MHz, CDCl$_3$): δ 8.6 (t, 2 x C3'''), 13.0 (d, C2'''), 33.3 (t, C4-C), 42.6 (d, C4), 49.2 (d, C3), 56.0 (q, 4 x Ar-OCH$_3$), 62.8 (t, C3-C), 72.9 (t, C5), 83.1 (d, C2), 109.0 (d, C2'), 111.1 (d, C5'), 111.4 (d, C5''*), 112.0 (d, C2''*), 118.2 (d, C6'), 120.5 (d, C6''), 132.7 (s, C1''), 135.1 (s, C1'), 147.6 (s, C4''), 148.6 (s, C4'), 149.0 (s, C3''), 149.1 (s, C3'), 174.8 (s, C1''').

B.6 ((2*S*,3*R*,4*R*)- 4-(3,4-Dimethoxybenzyl)-2-(3,4-dimethoxyphenyl)tetrahydrofuran-3-yl)methyl cyclohexanecarboxylate (40)

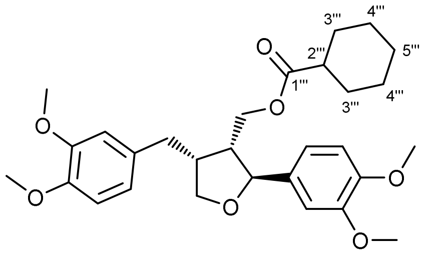

40

Chemical Formula: $C_{29}H_{38}O_7$

Molecular Weight: 498.61 gmol-1

Preparation: analogous to **36**, using starting material **21** (35.0 mg, 0.090 mmol, 1.00 equiv.), cyclohexanecarboxylic acid (26.5 mg, 0.207 mmol, 2.3 equiv.), EDCI.HCl (34.5 mg, 0.180 mmol, 2.0 equiv.), 4-DMAP (1.1 mg, 9.0 µmol, 0.1 equiv.) and DIPEA (39 µL, 0.23 mmol, 2.5 equiv.). The reaction solution was used directly for flash column chromatography (9 g silica, flow rate 20 mL / min, EtOAc / LP, 10 : 90 to 22 : 78 in 9 min, then 22 : 78 isocratically for 6 min, then to 62 : 38 in 30 min).

Yield: 33.3 mg, (74 %)

Appearance: colorless oil

***R*f (silica)**: 0.62 (EtOAc / LP, 1 : 1)

$[\alpha]_D^{20}$: +16.8 (c 1.62, MeOH)

LC-HRMS (ESI): calculated for $M+Na^+$: 521.2510, found: 521.2516, Δ: 1.15 ppm

(log *P*)calc: 5.75 ± 0.44

GC-MS (EI, 70 eV, Method D): 40.73 min; 498.2 (M^+, 3), 219.1 (26), 165.1 (53), 151.1 (100).

^{1}H NMR (200 MHz, $CDCl_3$): δ 1.12 – 1.96 (m, 10H, 4 x H3''', 4 x H4''', 2 x H5'''), 2.18 – 2.35 (m, 1H, H2'''), 2.46 – 2.83 (m, 3H, H3, H4, C4-CH), 2.86 (dd, ^2J = 12.4 Hz, ^3J = 4.3 Hz, 1H, C4-CH), 3.76 (dd, ^2J = 8.6 Hz, ^3J = 6.2 Hz, 1H, H5), 3.87 (s, 3H, Ar-OCH_3), 3.87 (s, 6H, Ar-OCH_3), 3.89 (s, 3H, Ar-OCH_3), 4.07 (dd, ^2J = 8.6 Hz, ^3J = 6.3 Hz, 1H, H5), 4.17 (dd, ^2J = 11.3 Hz, ^3J = 6.9 Hz, 1H, C3-CH), 4.37 (dd, ^2J = 11.3 Hz, ^3J = 6.9 Hz, 1H, C3-CH), 4.80 (d, ^3J = 6.4 Hz, 1H, H2), 6.66 – 6.91 (m, 6H, Ar-H).

^{13}C NMR (50 MHz, $CDCl_3$): δ 25.5 (t, 2 x C4'''), 25.8 (t, C5'''), 29.1 (t, 2 x C3'''), 29.1 (t, C3'''), 33.3 (t, C4-C), 42.7 (d, C4), 43.3 (d, C2'''), 49.3 (d, C3), 56.0 (q, 4 x Ar-OCH_3), 62.4 (t, C3-C), 72.9 (t, C5), 83.0 (d, C2), 109.0 (d, C2'), 111.1 (d, C5'), 111.4 (d, C5''*), 112.0 (d, C2''*), 118.2 (d, C6'), 120.6 (d, C6''), 132.7 (s, C1''), 135.1 (s, C1'), 147.6 (s, C4''), 148.6 (s, C4'), 149.1 (s, C3''), 149.2 (s, C3'), 176.0 (s, C1''').

B.7 ((2*S*,3*R*,4*R*)- 2-(3,4-Dimethoxybenzyl)-4-(4-methoxybenzyl)tetrahydrofuran-3-yl) methanol (19)

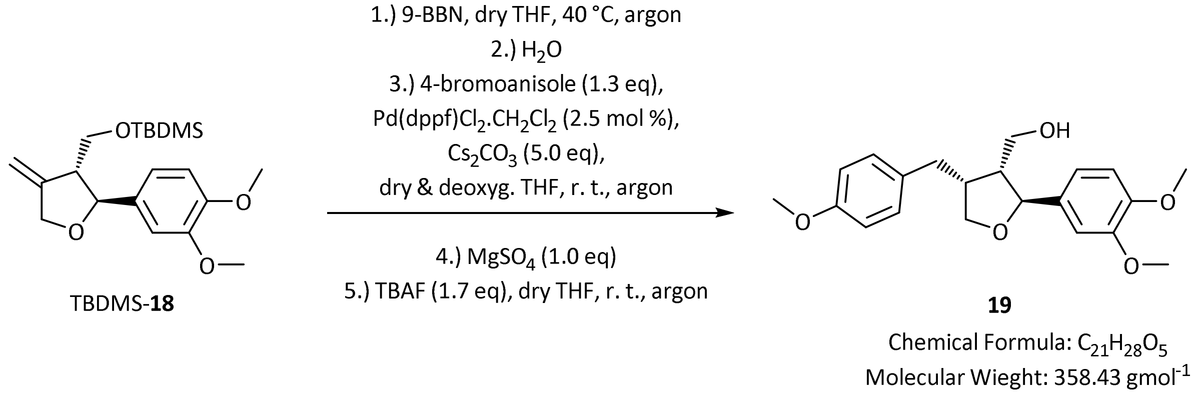

A reaction vessel was charged with a stirring bar and crude starting material TBDMS-**18** (715.9 mg, 1.964 mmol), and then evacuated and back-filled with argon using standard Schlenk technique. A solution of 9-BBN (0.5 M in THF, 5.89 mL, 2.95 mmol) was added via syringe, the reaction was stirred for 21 h at 40 °C and then allowed to cool to room temperature. Water (35 µL, 2.0 mmol) was subsequently added and stirring was continued for 2 h to decompose excess 9-BBN. This mixture was then purged by bubbling argon into the solution through a needle, and dry and deoxygenated THF was added to produce a total volume of 10.0 mL, i.e. a 0.196 M solution of borylated intermediate, thus allowing the use of aliquots for subsequent coupling. An aliquot (0.97 mL, 0.19 mmol, 1.0 equiv.) of this solution was transferred via syringe to a separate vessel which had been charged with a stirring bar, 4-bromoanisole (46.2 mg, 0.247 mmol, 1.3 equiv.), $Pd(dppf)Cl_2.CH_2Cl_2$ (3.9 mg, 4.8 µmol, 2.5 mol %) and Cs_2CO_3 (310 mg, 0.950 mmol, 5.0 equiv.) under argon and was stirred for 36 h at room temperature. Following this, $MgSO_4$ (23 mg, 0.19 mmol, 1.0 equiv.) was added and stirring was continued for 1.5 h to remove residual water. For deprotection, a solution of TBAF (1.0 M in THF, 0.32 mL, 0.32 mmol, 1.7 equiv.) was added via syringe and the mixture was finally stirred for 32 h at room temperature. The heterogeneous reaction content was filtered and rinsed with EtOAc (20 mL) and the solvents were evaporated. Flash column chromatography (18 g silica, flow rate 20 mL / min, EtOAc / LP, 30 : 70 to 80 : 20 in 30 min) afforded the title compound **19**.

Yield: 41.7 mg, (61 %)

Appearance light-brown oil

***R*f (silica)**: 0.66 (EtOAc)

$[\alpha]_D^{25}$: +13.0 (c 4.17, MeOH)

LC-HRMS (ESI): calculated for $M+Na^+$: 381.1678, found: 381.1689, Δ: 2.89 ppm

(log *P*)calc: 3.36 ± 0.55

^{1}H NMR (200 MHz, $CDCl_3$): δ 1.69 (t, ^3J = 4.8 Hz, 1H, OH), 2.39 (quint, ^3J = 6.7 Hz, 1H, H3), 2.55 (dd, ^2J = 12.6 Hz, ^3J = 10.1 Hz, 1H, C4-CH), 2.63 – 2.83 (m, 1H, H4), 2.90 (dd, ^2J = 12.7 Hz, ^3J = 4.7 Hz, 1H, C4-CH), 3.67 – 3.96 (m, 3H, C3-CH_2, H5), 3.78 (s, 3H, C4"-OCH_3), 3.86 (s, 3H, Ar'-OCH_3), 3.87 (s, 3H, Ar'-OCH_3), 4.05 (dd, ^2J = 8.5 Hz, ^3J = 6.4 Hz, 1H, H5), 4.82 (d, ^3J = 6.3 Hz, 1H, H2), 6.77 – 6.93 (m, 5H, 3 x Ar'-H, H3", H5"), 7.11 (d, ^3J = 8.6 Hz, 2H, H2", H6").

^{13}C NMR (50 MHz, $CDCl_3$): δ 32.8 (t, C4-C̲), 42.4 (d, C4), 52.5 (d, C3), 55.3 (q, C4''-OC̲H3), 55.97 (q, Ar'-OCH3), 56.02 (q, Ar'-OCH3), 61.0 (t, C3-C̲), 73.0 (t, C5), 82.9 (d, C2), 109.0 (d, C2'), 111.0 (d, C5'), 114.1 (d, C3'', C5''), 118.1 (d, C6'), 129.6 (d, C2'', C6''), 132.5 (s, C1''), 135.6 (s, C1'), 148.4 (s, C4'), 149.1 (s, C3'), 158.1 (s, C4'').

B.8 ((2*S*,3*R*,4*R*)- 2-(3,4-Dimethoxybenzyl)-4-(3-methoxybenzyl)tetrahydrofuran-3-yl)methanol (20)

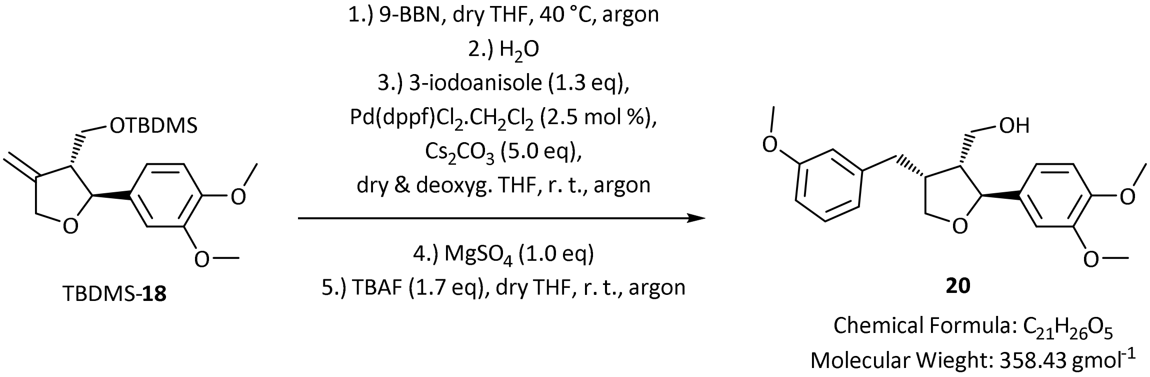

A reaction vessel was charged with a stirring bar and crude starting material TBDMS-**18** (843.2 mg, 2.313 mmol), and then evacuated and back-filled with argon using standard Schlenk technique. A solution of 9-BBN (0.5 M in THF, 6.94 mL, 3.47 mmol) was added *via* syringe, the reaction was stirred for 35 h at 40 °C and then allowed to cool to room temperature. Water (42 µL, 2.3 mmol) was subsequently added and stirring was continued for 2 h to decompose excess 9-BBN. This mixture was then purged by bubbling argon into the solution through a needle, and dry and deoxygenated THF was added to produce a total volume of 13.0 mL, i.e. a 0.178 M solution of borylated intermediate, thus allowing the use of aliquots for subsequent coupling.

An aliquot (1.07 mL, 0.19 mmol, 1.0 equiv.) of this solution was transferred *via* syringe to a separate vessel which had been charged with a stirring bar, 3-iodoanisole (57.8 mg, 0.247 mmol, 1.3 equiv.), Pd(dppf)Cl_2.CH_2Cl_2 (3.9 mg, 4.8 µmol, 2.5 mol %) and Cs_2CO_3 (310 mg, 0.950 mmol, 5.0 equiv.) under argon and was stirred for 50 h at room temperature. Following this, $MgSO_4$ (23 mg, 0.19 mmol, 1.0 equiv.) was added and stirring was continued for 1.5 h to remove residual water. For deprotection, a solution of TBAF (1.0 M in THF, 0.32 mL, 0.32 mmol, 1.7 equiv.) was added *via* syringe and the mixture was finally stirred for 24 h at room temperature. The heterogeneous reaction content was filtered and rinsed with CH_2Cl_2 (20 mL) and the solvents were evaporated. Flash column chromatography (18 g silica, flow rate 20 mL / min, EtOAc / LP, 15 : 85 to 85 : 15 in 40 min) afforded the title compound **20**.

Yield: 52.6 mg, (77 %)

Appearance: pale brown oil

***R*f (silica)**: 0.58 (EtOAc)

$[\alpha]_D^{25}$: +19.4 (c 3.69, MeOH)

LC-HRMS (ESI):calculated for $M+Na^+$: 381.1672, found: 381.1681, Δ: 2.36 ppm

(log *P*)calc: 3.36 ± 0.55

^{1}H NMR (200 MHz, $CDCl_3$): δ 1.87 (bs, 1H, OH), 2.40 (quint, ^3J = 6.8 Hz, 1H, H3), 2.58 (dd, ^2J = 12.6 Hz, ^3J = 10.3 Hz, 1H, C4-CH), 2.66 – 2.86 (m, 1H, H4), 2.94 (dd, ^2J = 12.7 Hz, ^3J = 4.7 Hz, 1H, C4-CH), 3.68 – 3.94 (m, 3H, C3-CH_2, H5), 3.78 (s, 3H, C3''-OCH_3), 3.85 (s, 3H, Ar'-OCH_3), 3.86 (s, 3H, Ar'-OCH_3), 4.06 (dd, ^2J = 8.5 Hz, ^3J = 6.4 Hz, 1H, H5), 4.82 (d, ^3J = 6.3 Hz, 1H, H2), 6.70 – 6.91 (m, 6H, 3 x Ar'-H, 3 x Ar''-H), 7.15 – 7.25 (m, 1H, H5'').

^{13}C NMR (50 MHz, $CDCl_3$): δ 33.6 (t, C4-C), 42.1 (d, C4), 52.5 (d, C3), 55.2 (q, C3''-OCH_3), 55.9 (q, Ar'-OCH_3), 56.0 (q, Ar'-OCH_3), 60.8 (t, C3-C), 73.0 (t, C5), 82.8 (d, C2), 108.9 (d, C2'), 111.0 (d, C5'*), 111.4 (d, C4''*), 114.6 (d, C2''), 118.1 (d, C6'), 121.1 (d, C6''), 129.6 (d, C5''), 135.5 (s, C1'), 142.2 (s, C1''), 148.4 (s, C4'), 149.1 (s, C3'), 159.8 (s, C3'').

B.9 ((2S,3R,4R)- 4--(3,4-Dimethoxyphenyl)-2- (3,4-dimethoxyphenyl)tetrahydrofuran-3-yl)methanol (21)

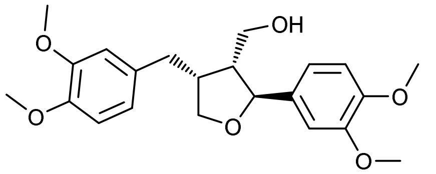

21

Chemical Formula :$C_{22}H_{28}O_6$

Molecular Weight: 388.45 g mol^{-1}

A reaction vessel was charged with a stirring bar and crude starting material TBDMS-**18** (2.51 g, 6.88 mmol, 1.0 equiv.), and then evacuated and back-filled with argon using standard Schlenk technique. A solution of 9-BBN (0.5 M in THF, 20.7 mL, 10.3 mmol, 1.5 equiv.) was added *via* syringe, the reaction was stirred for 16.5 h at 40 °C and then allowed to cool to room temperature. Following this, a degassed aqueous solution of NaOH (2M, 20 mL) was added cautiously and stirring was continued for another 15 min. 4-Iodoveratrole (2.36 g, 8.95 mmol, 1.30 equiv.) and Pd(dppf)Cl_2.CH_2Cl_2 (161 mg, 0.198 mmol, 2.9 mol %) were then added, and the resulting biphasic mixture was stirred vigorously at room temperature for 25 h. Et_2O (200 mL) and brine (50 mL) were then added, the layers were separated, the aqueous phase was extracted with Et_2O (4 x 50 mL), the combined organic phases were dried with Na_2SO_4 and filtered into a new reaction vessel. From there, the solvent was evaporated, a stirring bar was added to the residue and the vessel was evacuated and back-filled with

argon. For deprotection, a solution of TBAF (1.0 M in THF, 8.25 mL, 8.25 mmol, 1.2 equiv.) was added *via* syringe and the mixture was finally stirred for 18 h at room temperature. Et_2O (200 mL) and brine (50 mL) was added, the layers were separated and the aqueous phase was extracted with Et_2O (4 x 50 mL) and EtOAc (2 x 50 mL). The combined organic phases were dried with Na_2SO_4, filtered and the solvents were evaporated. Flash column chromatography of the entire crude material in two sequential runs (first run: 90 g silica with 9 g pre-column, flow rate 40 mL / min, EtOAc / LP, 30 : 70 for 3 min, then to 100 : 0 in 60 min; second run: 90 g silica, flow rate 40 mL / min, EtOAc / LP, 45 : 55 to 85 : 15 in 60 min) afforded the title compound **21.**

Yield: 1.04 g, 39 % (over 4 steps from unprotected alcohol **18**)

Appearance: slightly colored oil

***R*f (silica)**: 0.43 (EtOAc)

$[\alpha]_D^{20}$: +19.2 (c 1.45, MeOH); lit.[279] **$[\alpha]_D^{25}$**: +19.4 (c 0.6, $CHCl_3$)

LC-HRMS (ESI): calculated for $M+Na^+$: 411.1778, found: 411.1783, Δ: 1.22 ppm

$(\log P)_{calc}$:3.18 ± 0.56

^{1}H NMR (200 MHz, $CDCl_3$): δ 1.52 (bs, 1H, OH), 2.42 (quint, ^3J = 6.9 Hz, 1H, H3), 2.56 (dd, ^2J = 12.7 Hz, ^3J = 10.4 Hz, 1H, C4-CH), 2.66 – 2.85 (m, 1H, H4), 2.94 (dd, ^2J = 12.8 Hz, ^3J = 4.7 Hz, 1H, C4-CH), 3.73 – 3.98 (m, 2H, C3-CH_2), 3.76 (dd, ^2J = 8.5 Hz, ^3J = 5.9 Hz, 1H, H5), 3.87 (s, 9H, Ar-OCH_3), 3.88 (s, 3H, Ar-OCH_3), 4.07 (dd, ^2J = 8.5 Hz, ^3J = 6.4 Hz, 1H, H5), 4.81 (d, ^3J = 6.5 Hz, 1H, H2), 6.70 – 6.81 (m, 3H, Ar-H), 6.81 – 6.91 (m, 3H, Ar-H).

^{13}C NMR (50 MHz, $CDCl_3$): δ 33.4 (t, C4-C), 42.5 (d, C4), 52.7 (d, C3), 56.1 (q, 4 x Ar-OCH_3), 61.1 (t, C3-C), 73.1 (t, C5), 82.9 (d, C2), 109.1 (d, C2'), 111.1 (d, C5'), 111.4 (d, C5''*), 112.0 (d, C2''*), 118.2 (d, C6'), 120.6 (d, C6''), 133.1 (s, C1''), 135.5 (s, C1'), 147.6 (s, C4''), 148.5 (s, C4'), 149.1 (s, C3''), 149.2 (s, C3').

B.10 ((2S,3R,4R)- 4-4-Benzyl-2-(3,4-dimethoxyphenyl)tetrahydrofuran-3-yl)methanol (22)

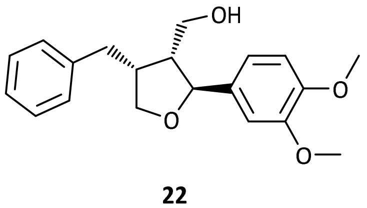

22

Chemical Formula: $C_{20}H_{24}O_4$

Molecular Weight: 328.40 $gmol^{-1}$

A reaction vessel was charged with a stirring bar and crude starting material TBDMS-**18** (715.9 mg, 1.964 mmol), and then evacuated and back-filled with argon using standard Schlenk technique. A solution of 9-BBN (0.5 M in THF, 5.89 mL, 2.95 mmol) was added *via* syringe, the reaction was stirred for 21 h at 40 °C and then allowed to cool to room temperature. Water (35 µL, 2.0 mmol) was subsequently added and stirring was continued

for 2 h to decompose excess 9-BBN. This mixture was then purged by bubbling argon into the solution through a needle, and dry and deoxygenated THF was added to produce a total volume of 10.0 mL, i.e. a 0.196 M solution of borylated intermediate, thus allowing the use of aliquots for subsequent coupling. An aliquot (0.97 mL, 0.19 mmol, 1.0 equiv.) of this solution was transferred via syringe to a separate vessel which had been charged with a stirring bar, bromobenzene (38.8 mg, 0.247 mmol, 1.3 equiv.) Pd(dppf)Cl_2.CH_2Cl_2 (3.9 mg, 4.8 µmol, 2.5 mol %) and Cs_2CO_3 (310 mg, 0.950 mmol, 5.0 equiv.) under argon and was stirred for 36 h at room temperature. Following this, $MgSO_4$ (23 mg, 0.19 mmol, 1.0 equiv.) was added and stirring was continued for 1.5 h to remove residual water. For deprotection, a solution of TBAF (1.0 M in THF, 0.32 mL, 0.32 mmol, 1.7 equiv.) was added *via* syringe and the mixture was finally stirred for 32 h at room temperature. The heterogeneous reaction content was filtered and rinsed with CH_2Cl_2 (20 mL) and the solvents were evaporated. Flash column chromatography (18 g silica, flow rate 20 mL / min, EtOAc in LP, 20 : 80 to 70 : 30 in 30 min) afforded the title compound **22**.

Yield: 39.3 mg, (63 %)

Appearance: light-brown oil

***R*f (silica)**: 0.45 (EtOAc / LP, 2 : 1)

$[\alpha]_D^{23}$: +18.3 (c 3.80, MeOH)

LC-HRMS (ESI): calculated for $M+Na^+$: 351.1567, found: 351.1577, Δ: 2.85 ppm

(log *P*)calc: 3.44 ± 0.54

^{1}H NMR (200 MHz, $CDCl_3$): δ 1.70 (bs, 1H, OH), 2.40 (quint, ^3J = 6.8 Hz, 1H, H3), 2.61 (dd, ^2J = 12.5 Hz, ^3J = 10.2 Hz, 1H, C4-CH), 2.67 – 2.87 (m, 1H, H4), 2.96 (dd, ^2J = 12.6 Hz, ^3J = 4.6 Hz, 1H, C4-CH), 3.69 – 3.98 (m, 3H, C3-CH_2, H5), 3.85 (s, 3H, Ar'-OCH_3), 3.87 (s, 3H, Ar'-OCH_3), 4.05 (dd, ^2J = 8.5 Hz, ^3J = 6.4 Hz, 1H, H5), 4.83 (d, ^3J = 6.3 Hz, 1H, H2), 6.77 – 6.94 (m, 3H, Ar'-H), 7.14 – 7.35 (m, 5H, Ar''-H).

^{13}C NMR (50 MHz, $CDCl_3$): δ 33.7 (t, C4-C), 42.2 (d, C4), 52.5 (d, C3), 55.98 (q, Ar'-OCH_3), 56.02 (q, Ar'-OCH_3), 61.0 (t, C3-C), 73.0 (t, C5), 82.9 (d, C2), 109.0 (d, C2'), 111.1 (d, C5'), 118.1 (d, C6'), 126.3 (d, C4''), 128.7 (d, C2''*, C6''*), 128.7 (d, C3''*, C5''*), 135.6 (s, C1'), 140.5 (s, C1''), 148.4 (s, C4'), 149.1 (s, C3').

B.11 (2S,3R,4R)-2-(3,4-Dimethoxyphenyl)-4-(4-(trifluoromethyl)benzyl)tetrahydro furan-3- yl)methanol (23)

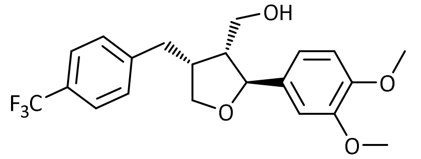

23

Chemical Formula: $C_{21}H_{23}F_3O_4$

Molecular Wieght: 396.40 $gmol^{-1}$

Preparation: analogous to **20**, using 1-bromo-4-(trifluoromethyl)benzene (55.6 mg, 0.247 mmol, 1.3 equiv.) as aryl halide coupling partner. The heterogeneous reaction content was filtered and rinsed with CH_2Cl_2 (20 mL) and the solvents were evaporated. Flash column chromatography (18 g silica, flow rate 20 mL / min, EtOAc / LP, 15 : 85 to 80 : 20 in 45 min) afforded the title compound **23**.

Yield: 57.2 mg, (76 %)

Appearance: nearly colorless oil

R_f (silica): 0.59 (EtOAc)

$[\alpha]_D^{25}$: +18.7 (c 2.68, MeOH)

LC-HRMS (ESI): calculated for $M+Na^+$: 419.1441, found: 419.1437, Δ: -0.95 ppm

(log *P*)$_{calc}$: 4.01 ± 0.58

^{1}H NMR (200 MHz, $CDCl_3$): δ 1.79 (bs, 1H, OH), 2.42 (quint, ^3J = 6.7 Hz, 1H, H3), 2.60 – 2.86 (m, 2H, H4, C4-CH), 3.04 (d, ^2J = 11.4 Hz, 1H, C4-CH), 3.71 (dd, ^2J = 8.6 Hz, ^3J = 5.9 Hz, 1H, H5), 3.76 – 3.97 (m, 2H, C3-CH_2), 3.86 (s, 3H, Ar'-OCH_3), 3.87 (s, 3H, Ar'-OCH_3), 4.03 (dd, ^2J = 8.6 Hz, ^3J = 6.2 Hz, 1H, H5), 4.82 (d, ^3J = 6.4 Hz, 1H, H2), 6.78 – 6.91 (m, 3H, Ar'-H), 7.31 (d, ^3J = 8.0 Hz, 2H, H2'', H6''), 7.55 (d, ^3J = 8.2 Hz, 2H, H3'', H5'').

^{13}C NMR (50 MHz, $CDCl_3$): δ 33.4 (t, C4-C̲), 42.1 (d, C4), 52.4 (d, C3), 55.99 (q, Ar'-OCH_3), 56.02 (q, Ar'-OCH_3), 60.8 (t, C3-C̲), 72.7 (t, C5), 82.8 (d, C2), 108.9 (d, C2'), 111.1 (d, C5'), 118.1 (d, C6'), 124.3 (q, C4''-C̲F_3, $^1J_{C\text{-}F}$ = 271.7 Hz), 125.6 (dq, C3'', C5'', $^3J_{C\text{-}F}$ = 3.8 Hz), 128.7 (q, C4'', $^2J_{C\text{-}F}$ = 32.3 Hz), 129.1 (d, C2'', C6''), 135.3 (s, C1'), 144.8 (q, C1'', $^5J_{C\text{-}F}$ = 1.3 Hz), 148.5 (s, C4'), 149.2 (s, C3').

B.12 ((2S,3R,4R)-2-(3,4-Dimethoxyphenyl)-4-(4-fluorobenzyl)tetrahydrofuran-3-yl) methanol (24)

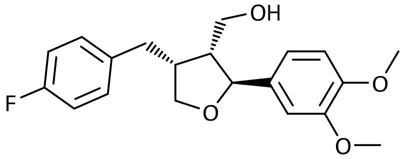

24

Chemical Formula: $C_{20}H_{23}FO_4$

Molecular Weight: 346.39 gmol-1

Preparation: analogous to **19**, using 1-bromo-4-fluorobenzene (43.2 mg, 0.247 mmol, 1.3 equiv.) as aryl halide coupling partner. The heterogeneous reaction content was filtered and rinsed with CH_2Cl_2 (20 mL) and the solvents were evaporated. Flash column chromatography (18 g silica, flow rate 20 mL / min, EtOAc / LP, 30 : 70 to 50 : 50 in 15 min, then to 85 : 15 in 5 min, then 85 : 15 isocratically) afforded the title compound **24**.

Yield: 40.2 mg, (61 %)

Appearance: light-brown oil

***R*f (silica)**: 0.59 (EtOAc)

[α]D^{25}: +18.5 (c 3.82, MeOH)

LC-HRMS (ESI): calculated for M+Na$^+$: 369.1473, found: 369.1475, Δ: 0.54 ppm

(log *P*)calc: 3.49 ± 0.60

^{1}H NMR (200 MHz, CDCl$_3$): δ 1.66 (bs, 1H, OH), 2.40 (quint, ^3J = 6.7 Hz, 1H, H3), 2.58 (dd, ^2J = 12.4 Hz, ^3J = 10.6 Hz, 1H, C4-CH), 2.62 – 2.82 (m, 1H, H4), 2.95 (dd, ^2J = 12.5 Hz, ^3J = 4.1 Hz, 1H, C4-CH), 3.71 (dd, ^2J = 8.5 Hz, ^3J = 6.1 Hz, 1H, H5), 3.75 – 3.96 (m, 2H, C3-CH$_2$), 3.86 (s, 3H, Ar'-OCH$_3$), 3.87 (s, 3H, Ar'-OCH$_3$), 4.04 (dd, ^2J = 8.6 Hz, ^3J = 6.3 Hz, 1H, H5), 4.82 (d, ^3J = 6.3 Hz, 1H, H2), 6.79 – 6.90 (m, 3H, Ar'-H), 6.91 – 7.04 (m, 2H, H3'', H5''), 7.08 – 7.20 (m, 2H, H2'', H6'').

^{13}C NMR (50 MHz, CDCl$_3$): δ 32.8 (t, C4-C), 42.4 (d, C4), 52.5 (d, C3), 56.00 (q, Ar'-OCH$_3$), 56.04 (q, Ar'-OCH$_3$), 60.9 (t, C3-C), 72.8 (t, C5), 82.9 (d, C2), 109.0 (d, C2'), 111.1 (d, C5'), 115.4 (dd, C3'', C5'', $^2J_{C-F}$ = 21.2 Hz), 118.1 (d, C6'), 130.1 (dd, C2'', C6'', $^3J_{C-F}$ = 7.8 Hz), 135.5 (s, C1'), 136.2 (d, C1'', $^4J_{C-F}$ = 3.2 Hz), 148.5 (s, C4'), 149.2 (s, C3'), 161.5 (d, C4'', $^1J_{C-F}$ = 244.2 Hz).

B.13 ((2S,3R,4R)-2-(3,4-Dimethoxyphenyl)-4-(4-methylbenzyl)tetrahydrofuran-3-yl) methanol (25)

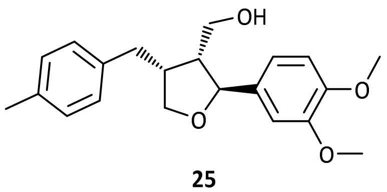

25
Chemical Formula: $C_{21}H_{26}O_4$
Molecular Weight: 342.43 gmol^{-1}

Preparation: analogous to **19**, using 4-bromotoluene (42.2 mg, 0.247 mmol, 1.3 equiv.) as aryl halide coupling partner. The heterogeneous reaction content was filtered and rinsed with CH_2Cl_2 (20 mL) and the solvents were evaporated. Flash column chromatography (18 g silica, flow rate 20 mL / min, EtOAc in LP, 20 : 80 to 70 : 30 in 30 min) afforded the title compound **25**.

Yield: 43.2 mg, (66 %)

Appearance: light-brown oil

***R*f (silica)**: 0.47 (EtOAc / LP, 2 : 1)

[α]D^{23}: +15.0 (c 4.34, MeOH)

LC-HRMS (ESI): calculated for M+Na$^+$: 365.1723, found: 365.1727, Δ: 1.10 ppm

(log *P*)calc: 3.90 ± 0.54

^{1}H NMR (200 MHz, CDCl$_3$): δ 1.72 (t, ^3J = 5.0 Hz, 1H, OH), 2.31 (s, 3H, C4''-CH$_3$), 2.39 (quint, ^3J = 6.7 Hz, 1H, H3), 2.57 (dd, ^2J = 12.6 Hz, ^3J = 10.0 Hz, 1H, C4-CH), 2.65 – 2.85 (m, 1H, H4), 2.91 (dd, ^2J = 12.6 Hz, ^3J = 4.8 Hz, 1H, C4-CH), 3.66 – 3.98 (m, 3H, C3-CH$_2$, H5), 3.85 (s, 3H, Ar'-OCH$_3$), 3.87 (s, 3H, Ar'-OCH$_3$), 4.05 (dd, ^2J = 8.5 Hz, ^3J = 6.4 Hz, 1H, H5), 4.82 (d, ^3J = 6.3 Hz, 1H, H2), 6.77 – 6.90 (m, 3H, Ar'-H), 7.02 – 7.13 (m, 4H, Ar''-H).

^{13}C NMR (50 MHz, CDCl$_3$): δ 21.1 (q, C4''-<u>C</u>H$_3$), 33.2 (t, C4-<u>C</u>), 42.3 (d, C4), 52.5 (d, C3), 55.9 (q, Ar'-OCH$_3$), 56.0 (q, Ar'-OCH$_3$), 60.9 (t, C3-<u>C</u>), 73.1 (t, C5), 82.9 (d, C2), 109.0 (d, C2'), 111.0 (d, C5'), 118.1 (d, C6'), 128.6 (d, C2'', C6''), 129.3 (d, C3'', C5''), 135.6 (s, C1'), 135.8 (s, C4''), 137.4 (s, C1''), 148.4 (s, C4'), 149.1 (s, C3').

B.14 ((2S,3R,4R)-4-(4-(tert-Butyl)benzyl)-2-(3,4-dimethoxyphenyl)tetrahydrofuran-3-yl) methanol (26)

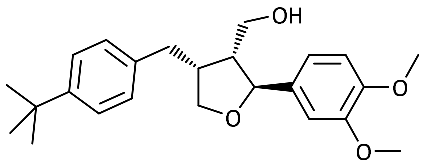

26
Chemical Formula: $C_{24}H_{32}O_4$
Molecular Weight: 384.51 gmol^{-1}

Preparation: analogous to **19**, using 1-bromo-4-(*tert*-butyl)benzene (52.6 mg, 0.247 mmol, 1.3 equiv.) as aryl halide coupling partner. The heterogeneous reaction content was filtered and rinsed with CH_2Cl_2 (20 mL) and the solvents were evaporated. Flash column chromatography (18 g silica, flow rate 20 mL / min, EtOAc in LP, 20 : 80 to 70 : 30 in 30 min) afforded the title compound **26**.

Yield: 49.1 mg, (67 %)

Appearance: light-brown oil

***R*f (silica)**: 0.76 (EtOAc)

[α]D^{25}: +12.0 (c 4.91, MeOH)

LC-HRMS (ESI): calculated for M+Na$^+$: 407.2193, found: 407.2183, Δ: -2.46 ppm

(log *P*)calc: 5.13 ± 0.55

^{1}H NMR (200 MHz, CDCl$_3$): δ 1.31 (s, 9H, C4''-C(CH$_3$)$_3$), 1.68 (bs, 1H, OH), 2.40 (quint, ^3J = 6.7 Hz, 1H, H3), 2.59 (dd, ^2J = 12.5 Hz, ^3J = 9.8 Hz, 1H, C4-CH), 2.67 – 2.86 (m, 1H, H4), 2.92 (dd, ^2J = 12.6 Hz, ^3J = 4.8 Hz, 1H, C4-CH), 3.69 – 3.98 (m, 3H, C3-CH$_2$, H5), 3.86 (s, 3H, Ar'-OCH$_3$), 3.87 (s, 3H, Ar'-OCH$_3$), 4.08 (dd, ^2J = 8.5 Hz, ^3J = 6.4 Hz, 1H, H5), 4.83 (d, ^3J = 6.2 Hz, 1H, H2), 6.78 – 6.94 (m, 3H, Ar'-H), 7.12 (d, ^3J = 8.2 Hz, 2H, H2'', H6''), 7.31 (d, ^3J = 8.2 Hz, 2H, H3'', H5'').

^{13}C NMR (50 MHz, CDCl$_3$): δ 31.5 (q, C4''-C($\underline{C}$H$_3$)$_3$), 33.1 (t, C4-$\underline{C}$), 34.5 (s, C4''-$\underline{C}$(CH$_3$)$_3$), 42.2 (d, C4), 52.5 (d, C3), 56.00 (q, Ar'-OCH$_3$), 56.03 (q, Ar'-OCH$_3$), 61.0 (t, C3-$\underline{C}$), 73.2 (t, C5), 82.9 (d, C2), 109.0 (d, C2'), 111.1 (d, C5'), 118.1 (d, C6'), 125.5 (d, C3'', C5''), 128.4 (d, C2'', C6''), 135.7 (s, C1'), 137.4 (s, C1''), 148.4 (s, C4'), 149.1 (s, C3'; s, C4''; signal overlap).

B.15 Z)-((2S,3R,4R)-2-(3,4-Dimethoxyphenyl)-4-(4-methoxybenzyl)tetrahydrofuran-3-yl)methyl 2-methylbut-2-enoate (27)

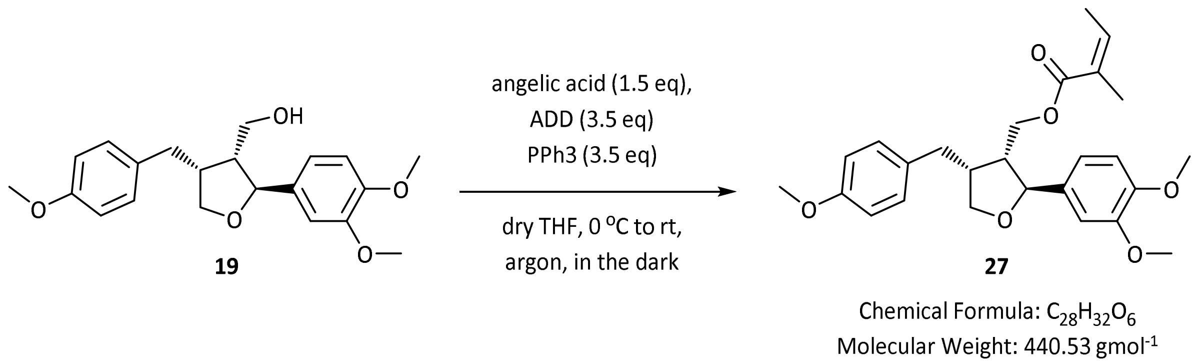

Preparation: a reaction vessel was charged with a stirring bar, starting material **19** (24.0 mg, 0.067 mmol, 1.0 equiv.), angelic acid (7.6 mg, 0.076 mmol, 1.5 equiv.) and PPh$_3$ (46.5 mg, 0.177 mmol, 3.5 equiv.), and then evacuated and back-filled with argon using standard Schlenk technique. Dry THF (0.75 mL) was then added *via* syringe and the solution cooled to 0 °C in an ice bath. To the stirred mixture was then added a solution of ADD (44.7 mg, 0.177 mmol, 3.5 equiv.) in dry THF (1.0 mL) *via* syringe over approximately 1 min, and the reaction stirred for 22 h while being kept away from light and allowed to warm slowly to room temperature. Et$_2$O (20 mL) was added to the reaction content, which was then filtered and rinsed with more Et$_2$O (10 mL). The solvents were evaporated and flash column chromatography was performed (9 g silica, flow rate 20 mL / min, EtOAc / LP, 10 : 90 isocrtically for 3 min, then 40 : 60 in 30 min) to afford the title compound **27**.

Yield: 24.0 mg, (81 %)

Appearance: colorless oil

***R*$_f$ (silica)**: 0.67 (EtOAc / LP, 1 : 1)

[α]$_D^{25}$: +25.1 (c 2.35, MeOH)

LC-HRMS (ESI): calculated for M+Na$^+$: 463.2091, found: 463.2058, Δ: -7.12 ppm

(log *P*)calc: 5.56 ± 0.46

GC-MS (EI, 70 eV, Method D): 18.75 min; 440.2 (M^+, 3), 219.1 (31), 166.1 (17), 165.1 (89), 160.1 (17), 159.1 (20), 151.1 (17), 147.1 (23), 121.0 (100).

^{1}H NMR (200 MHz, $CDCl_3$): δ 1.85 – 1.90 (m, 3H, H5'''), 2.00 (dq, ^3J = 7.2 Hz, ^5J = 1.5 Hz, 3H, H4'''), 2.49 – 2.82 (m, 3H, H3, H4, C4-CH), 2.89 (dd, ^2J = 12.5 Hz, ^3J = 4.3 Hz, 1H, C4-CH), 3.76 (dd, ^2J = 8.6 Hz, ^3J = 6.2 Hz, 1H, H5), 3.79 (s, 3H, C4''-OCH_3), 3.87 (s, 3H, Ar'-OCH_3), 3.88 (s, 3H, Ar'-OCH_3), 4.07 (dd, ^2J = 8.6 Hz, ^3J = 6.2 Hz, 1H, H5), 4.27 (dd, ^2J = 11.4 Hz, ^3J = 6.9 Hz, 1H, C3-CH), 4.40 (dd, ^2J = 11.3 Hz, ^3J = 6.9 Hz, 1H, C3-CH), 4.84 (d, ^3J = 6.2 Hz, 1H, H2), 6.10 (qq, ^3J = 7.2 Hz, ^4J = 1.4 Hz, 1H, H3'''), 6.79 – 6.89 (m, 5H, 3 x Ar'-H, H3'', H5''), 7.09 (d, ^3J = 8.6 Hz, 2H, H2'', H6'').

^{13}C NMR (50 MHz, $CDCl_3$): δ 16.0 (q, C4'''), 20.7 (q, C5'''), 32.8 (t, C4-C), 42.7 (d, C4), 49.3 (d, C3), 55.4 (q, C4''-OCH_3), 56.0 (q, Ar'-OCH_3), 56.1 (q, Ar'-OCH_3), 62.3 (t, C3-C), 72.8 (t, C5), 83.0 (d, C2), 109.0 (d, C2'), 111.1 (d, C5'), 114.1 (d, C3'', C5''), 118.1 (d, C6'), 127.5 (s, C2'''), 129.6 (d, C2'', C6''), 132.2 (s, C1''), 135.1 (s, C1'), 139.0 (d, C3'''), 148.6 (s, C4'), 149.2 (s, C3'), 158.2 (s, C4''), 167.9 (s, C1''').

B.16 (*Z*)-((2*S*,3*R*,4*R*)-2-(3,4-Dimethoxyphenyl)-4-(3-methoxybenzyl)tetrahydrofuran-3-yl)methyl 2-methylbut-2-enoate (28)

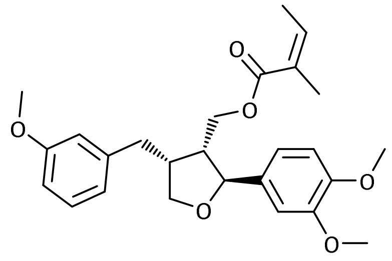

28
Chemical Formula: $C_{28}H_{32}O_6$
Molecular Weight: 440.53 $gmol^{-1}$

***Preparation**:* analogous to **27**, using starting material **20** (28.1 mg, 0.078 mmol, 1.00 equiv.). Et_2O (5 mL) was added to the reaction content, which was then filtered and rinsed with more Et_2O (15 mL). The solvents were evaporated and flash column chromatography (9 g silica, flow rate 20 mL / min, EtOAc / LP, 1 : 99 to 10 : 90 in 5 min, then to 40 : 60 in 30).

Yield: 31.2 mg, 90 %

Appearance: colorless oil

***R*f (silica)**: 0.68 (EtOAc / cyclohexane, 1 : 1)

[α]$_D^{20}$: +30.4 (c 1.21, MeOH)

LC-HRMS (ESI): calculated for M+Na^+: 463.2091, found: 463.2109, Δ: 3.89 ppm

(log *P*)calc: 5.56 ± 0.46

GC-MS (EI, 70 eV, Method D): 17.63 min; 440.1 (M^+, 6), 340.1 (18), 219.1 (36), 174.1 (28), 173.1 (18), 166.1 (24), 165.0 (100), 159.1 (28), 151.0 (37), 147.1 (23), 121.1 (58), 91.1 (23).

^{1}H NMR (200 MHz, $CDCl_3$): δ 1.85 – 1.91 (m, 3H, H5'''), 2.00 (dq, ^3J = 7.2 Hz, ^5J = 1.5 Hz, 3H, H4'''), 2.53 – 2.87 (m, 3H, H3, H4, C4-CH), 2.93 (dd, ^2J = 12.4 Hz, ^3J = 4.2 Hz, 1H, C4-CH), 3.77 (dd, ^2J = 8.7 Hz, ^3J = 6.3 Hz, 1H, H5), 3.80 (s, 3H, C3''-OCH_3), 3.87 (s, 3H, Ar'-OCH_3), 3.88 (s, 3H, Ar'-OCH_3), 4.08 (dd, ^2J = 8.6 Hz, ^3J = 6.3 Hz, 1H, H5), 4.27 (dd, ^2J = 11.4 Hz, ^3J = 6.9 Hz, 1H, C3-CH), 4.41 (dd, ^2J = 11.4 Hz, ^3J = 7.1 Hz, 1H, C3-CH), 4.84 (d, ^3J = 6.4 Hz, 1H, H2), 6.10 (qq, ^3J = 7.2 Hz, ^4J = 1.4 Hz, 1H, H3'''), 6.69 – 6.92 (m, 6H, 3 x Ar'-H, H2'', H4'', H6''), 7.23 (t, ^3J = 7.8 Hz, 1H, H5'').

^{13}C NMR (50 MHz, $CDCl_3$): δ 16.0 (q, C4'''), 20.7 (q, C5'''), 33.7 (t, C4-C), 42.4 (d, C4), 49.3 (d, C3), 55.3 (q, C3''-O$\underline{C}H_3$), 56.0 (q, Ar'-OCH_3), 56.1 (q, Ar'-OCH_3), 62.3 (t, C3-C), 72.8 (t, C5), 82.9 (d, C2), 108.9 (d, C2'), 111.1 (d, C5'*), 111.5 (d, C4''*), 114.6 (d, C2''), 118.2 (d, C6'), 121.1 (d, C6''), 127.5 (s, C2'''), 129.7 (d, C5''), 135.0 (s, C1'), 139.1 (d, C3'''), 141.8 (s, C1''), 148.6 (s, C4'), 149.2 (s, C3'), 159.9 (s, C3''), 167.8 (s, C1''').

B.17 (*Z*)-((2*S*,3*R*,4*R*)-4-(3,4-Dimethoxybenzyl)-2-(3,4- dimethoxyphenyl) tetrahydrofuran -3-yl)methyl 2-methylbut-2-enoate (leoligin, 29)

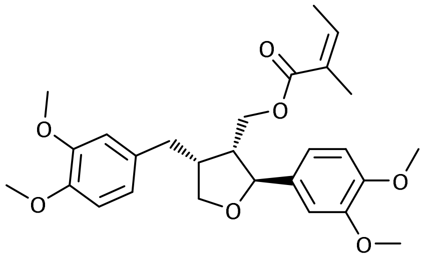

29
Chemical Formula: $C_{27}H_{34}O_7$
Molecular Weight: 470.55 $gmol^{-1}$

A reaction vessel was charged with a stirring bar, starting material **21** (989 mg, 2.55 mmol, 1.0 equiv.), angelic acid (383 mg, 3.83 mmol, 1.5 equiv.) and PPh_3 (2.34 g, 8.93 mmol, 3.5 equiv.), and then evacuated and back-filled with argon using standard Schlenk technique. Dry THF (20 mL) was then added and the solution cooled to 0 °C in an ice bath. To the stirred mixture was then added DEAD (1.40 mL, 8.93 mmol, 3.5 equiv.) dropwise *via* syringe, and the reaction stirred for 12 h while being kept away from light and allowed to warm slowly to room temperature. The solvent was evaporated, which was followed by the addition of $CHCl_3$ (15 mL), LP (300 mL) and water (200 mL). The layers were separated and the aqueous phase was re-extracted with LP (4 x 50 ml). The solvents were evaporated from the combined organic phases and then flash column chromatography was performed (180 g silica, flow rate 40 mL / min, EtOAc / LP, 25 : 75 to 50 : 50 in 60 min) to afford the title compound **29**. An analytical sample could be crystallized from a saturated solution of heptane and cooling it to -20 °C for several days.

Yield: 1.124 g, 94 %

Appearance: nearly colorless oil

Melting range:45.0 – 46.5 °C; lit.[153] **melting range**: n/a (natural compound obtained as a colorless amorphous substance)

***R*f (silica)**: 0.57 (EtOAc / LP, 1 : 1)

[α]$_D^{25}$: +23.4 (c 3.69, MeOH); lit.[153] **[α]$_D^{20}$**: +25 (c 0.002, CH_2Cl_2)

LC-HRMS (ESI): calculated for M+Na$^+$: 493.2197, found: 493.2201, Δ: 0.81 ppm

(log *P*)$_{calc}$:5.38 ± 0.48

GC-MS (EI, 70 eV, Method E): 23.65 min; 470.2 (M$^+$, 3), 219.1 (26), 189.1 (15), 177.1 (15), 165.1 (72), 151.0 (100), 107.1 (15).

^{1}H NMR (200 MHz, CDCl$_3$): δ 1.85 – 1.90 (m, 3H, H5'''), 2.00 (dq, ^3J = 7.2 Hz, ^5J = 1.5 Hz, 3H, H4'''), 2.49 – 2.85 (m, 3H, H3, H4, C4-CH), 2.90 (dd, ^2J = 12.4 Hz, ^3J = 4.2 Hz, 1H, C4-CH), 3.78 (dd, ^2J = 8.6 Hz, ^3J = 6.0 Hz, 1H, H5), 3.86 (s, 3H, Ar-OCH$_3$), 3.87 (s, 6H, Ar-OCH$_3$), 3.88 (s, 3H, Ar-OCH$_3$), 4.08 (dd, ^2J = 8.6 Hz, ^3J = 6.2 Hz, 1H, H5), 4.28 (dd, ^2J = 11.3 Hz, ^3J = 7.0 Hz, 1H, C3-CH), 4.42 (dd, ^2J = 11.3 Hz, ^3J = 7.0 Hz, 1H, C3-CH), 4.84 (d, ^3J = 6.3 Hz, 1H, H2), 6.10 (qq, ^3J = 7.2 Hz, ^4J = 1.3 Hz, 1H, H3'''), 6.67 – 6.75 (m, 2H, H2'', H6''), 6.77 – 6.90 (m, 4H, H2', H5', H6', H5'').

^{13}C NMR (100 MHz, CDCl$_3$): δ 16.0 (q, C4'''), 20.7 (q, C5'''), 33.3 (t, C4-C), 42.8 (d, C4), 49.3 (d, C3), 56.0 (q, 2 x Ar-OCH$_3$), 56.0 (q, Ar-OCH$_3$), 56.1 (q, Ar-OCH$_3$), 62.3 (t, C3-C), 72.8 (t, C5), 83.0 (d, C2), 109.0 (d, C2'), 111.2 (d, C5'), 111.4 (d, C5''*), 112.0 (d, C2''*), 118.2 (d, C6'), 120.6 (d, C6''), 127.5 (s, C2'''), 132.7 (s, C1''), 135.1 (s, C1'), 139.0 (d, C3'''), 147.6 (s, C4''), 148.6 (s, C4'), 149.1 (s, C3''), 149.2 (s, C3'), 167.8 (s, C1''').

B.18 (*Z*)-((2*S*,3*R*,4*R*)-4-Benzyl-2-(3,4-dimethoxyphenyl)tetrahydrofuran-3-yl)methyl 2-methylbut-2-enoate (30)

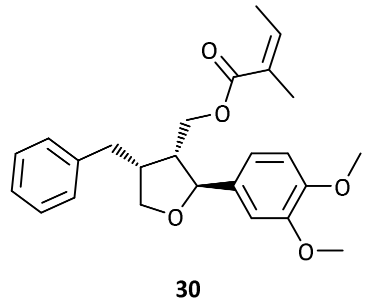

30

Chemical Formula: $C_{25}H_{30}O_5$
Molecular Weight: 410.50 gmol^{-1}

***Preparation**:* analogous to **27**, using starting material **22** (24.4 mg, 0.074 mmol, 1.00 equiv.) Et_2O (5 mL) was added to the reaction content, which was then filtered and rinsed with more Et_2O (15 mL). The solvents were evaporated and flash column chromatography (9 g silica, flow rate 20 mL/ min, EtOAc / LP, 1 : 99 to 35 : 65 in 30 min).

Yield: 26.4 mg, (86 %)

Appearance: colorless oil

***R*f (silica)**: 0.74 (EtOAc / LP, 1 : 1)

[α]D^25^: +31.6 (c 2.64, MeOH)

LC-HRMS (ESI): calculated for $M+Na^+$: 433.1985, found: 433.1968, Δ: -3.92 ppm

(log *P*)calc: 5.64 ± 0.44

GC-MS (EI, 70 eV, Method E): 13.41 min; 410.2 (M^+, 2), 219.1 (22), 166.1 (55), 165.0 (100), 151.0 (19), 117.1 (23), 91.0 (68).

^{1}H NMR (200 MHz, $CDCl_3$): δ 1.85 – 1.91 (m, 3H, H5'''), 2.00 (dq, ^3J = 7.3 Hz, ^5J = 1.5 Hz, 3H, H4'''), 2.70 – 2.53 (m, 2H, H3, C4-CH), 2.89 – 2.70 (m, 1H, H4), 2.95 (dd, ^2J = 12.5 Hz, ^3J = 4.1 Hz, 1H, C4-CH), 3.77 (dd, ^2J = 8.6 Hz, ^3J = 6.3 Hz, 1H, H5), 3.87 (s, 3H, Ar'-OCH_3), 3.88 (s, 3H, Ar'-OCH_3), 4.07 (dd, ^2J = 8.6 Hz, ^3J = 6.2 Hz, 1H, H5), 4.28 (dd, ^2J = 11.4 Hz, ^3J = 7.0 Hz, 1H, C3-CH), 4.41 (dd, ^2J = 11.4 Hz, ^3J = 7.0 Hz, 1H, C3-CH), 4.85 (d, ^3J = 6.3 Hz, 1H, H2), 6.10 (qq, ^3J = 7.2 Hz, ^4J = 1.4 Hz, 1H, H3'''), 6.92 – 6.79 (m, 3H, Ar'-H), 7.36 – 7.13 (m, 5H, Ar''-H).

^{13}C NMR (50 MHz, $CDCl_3$): δ 16.0 (q, C4'''), 20.7 (q, C5'''), 33.7 (t, C4-C), 42.5 (d, C4), 49.3 (d, C3), 56.0 (q, Ar'-OCH_3), 56.1 (q, Ar'-OCH_3), 62.3 (t, C3-C), 72.8 (t, C5), 83.0 (d, C2), 108.9 (d, C2'), 111.1 (d, C5'), 118.1 (d, C6'), 126.4 (d, C4''), 127.5 (s, C2'''), 128.7 (d, C2'', C6''; C3'', C5''; signal overlap), 135.1 (s, C1'), 139.1 (d, C3'''), 140.2 (s, C1''), 148.6 (s, C4'), 149.2 (s, C3'), 167.8 (s, C1''').

B.19(*Z*)-((2*S*,3*R*,4*R*)-2-(3,4-Dimethoxyphenyl)-4-(4-(trifluoromethyl)benzyl) tetrahydrofuran-3-yl)methyl 2-methylbut-2-enoate (31)

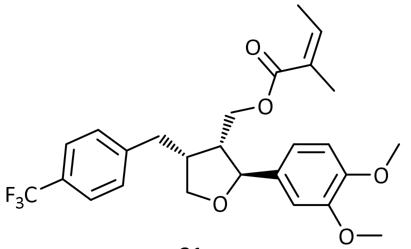

31

Chemical Formula: $C_{26}H_{29}F_3O_5$
Molecular Weight: 478.50 $gmol^{-1}$

***Preparation**:* analogous to **27**, using starting material **23** (32.7 mg, 0.082 mmol, 1.00 equiv.). Et_2O (5 mL) was added to the reaction content, which was then filtered and rinsed with more Et_2O (15 mL) The solvents were evaporated and flash column chromatography (9 g silica, flow rate 20 mL/ min, EtOAc / LP, 1 : 99 to 40 : 60 in 40 min).

Yield: 34.3 mg, (87 %)

Appearance: colorless oil

***R*f (silica)**: 0.74 (EtOAc / cyclohexane, 1 : 1)

[α]D²⁰: +28.5 (c 0.97, MeOH)

LC-HRMS (ESI): calculated for M+Na$^+$: 501.1859, found: 501.1882, Δ:4.59 ppm

(log *P*)calc: 6.21 ± 0.49

GC-MS (EI, 70 eV, Method D): 12.47 min; 478.1 (M$^+$, 2), 219.0 (28), 166.1 (39), 165.0 (100), 159.0 (25).

^{1}H NMR (200 MHz, CDCl$_3$): δ 1.85 – 1.90 (m, 3H, H5'''), 2.00 (dq, ^3J = 7.2 Hz, ^5J = 1.5 Hz, 3H, H4'''), 2.55 – 2.89 (m, 3H, H3, H4, C4-CH), 2.95 – 3.07 (m, 1H, C4-CH), 3.74 (dd, ^2J = 8.7 Hz, ^3J = 5.9 Hz, 1H, H5), 3.87 (s, 3H, Ar'-OCH$_3$), 3.88 (s, 3H, Ar'-OCH$_3$), 4.07 (dd, ^2J = 8.8 Hz, ^3J = 6.0 Hz, 1H, H5), 4.28 (dd, ^2J = 11.4 Hz, ^3J = 6.6 Hz, 1H, C3-CH), 4.40 (dd, ^2J = 11.4 Hz, ^3J = 7.1 Hz, 1H, C3-CH), 4.84 (d, ^3J = 6.4 Hz, 1H, H2), 6.12 (qq, ^3J = 7.2 Hz, ^4J = 1.3 Hz, 1H, H3'''), 6.79 – 6.92 (m, 3H, Ar'-H), 7.30 (d, ^3J = 8.1 Hz, 2H, H2'', H6''), 7.56 (d, ^3J = 8.1 Hz, 2H, H3'', H5'').

^{13}C NMR (50 MHz, CDCl$_3$): δ 16.0 (q, C4'''), 20.7 (q, C5'''), 33.6 (t, C4-C), 42.3 (d, C4), 49.3 (d, C3), 56.0 (q, Ar'-OCH$_3$), 56.1 (q, Ar'-OCH$_3$), 62.1 (t, C3-C), 72.6 (t, C5), 82.9 (d, C2), 108.9 (d, C2'), 111.2 (d, C5'), 118.2 (d, C6'), 124.3 (q, C4''-CF$_3$, $^1J_{C\text{-}F}$ = 272.0 Hz), 125.7 (dq, C3'', C5'', $^3J_{C\text{-}F}$ = 3.8 Hz), 127.4 (s, C2'''), 128.9 (q, C4'', $^2J_{C\text{-}F}$ = 32.3 Hz), 129.1 (d, C2'', C6''), 134.8 (s, C1'), 139.3 (d, C3'''), 144.4 (q, C1'', $^5J_{C\text{-}F}$ = 1.3 Hz), 148.7 (s, C4'), 149.2 (s, C3'), 167.8 (s, C1''').

B.20 (*Z*)-((2*S*,3*R*,4*R*)-2-(3,4-Dimethoxyphenyl)-4-(4-fluorobenzyl)tetrahydrofuran-3-yl)methyl 2-methylbut-2-enoate (32)

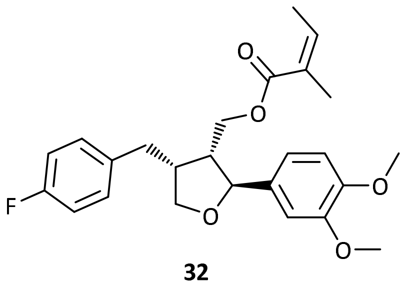

32

Chemical Formula: $C_{25}H_{29}O_5$
Molecular Weight: 428.49 gmol^{-1}

Preparation: analogous to **27**, using starting material **24** (29.8 mg, 0.086 mmol, 1.0 equiv.). Et$_2$O (5 mL) was added to the reaction content, which was then filtered and rinsed with more Et$_2$O (15 mL). The solvents were evaporated and flash column chromatography (9 g silica, flow rate 20 mL/ min, EtOAc / LP, 1 : 99 to 35 : 65 in 30 min).

Yield: 27.8 mg, (75 %)

Appearance: colorless oil

***R*f (silica)**: 0.73 (EtOAc / LP, 1 : 1)

[α]D25: +34.0 (c 1.04, MeOH)

LC-HRMS (ESI): calculated for M+Na$^+$: 451.1891, found: 451.1909, Δ: 3.99 ppm

(log *P*)calc: 0.69 ± 0.52

GC-MS (EI, 70 eV, Method E): 13.23 min; 428.2 (M$^+$, 3), 328.2 (15), 219.1 (24), 166.1 (48), 165.0 (100), 151.0 (17), 135.1 (22), 109.0 (68).

^{1}H NMR (200 MHz, $CDCl_3$): δ 1.83 – 1.91 (m, 3H, H5'''), 2.00 (d, ^3J = 7.8 Hz, 3H, H4'''), 2.51 – 2.85 (m, 3H, H3, H4, C4-CH), 2.92 (dd, ^2J = 12.5 Hz, ^3J = 3.9 Hz, 1H, C4-CH), 3.74 (dd, ^2J = 8.6 Hz, ^3J = 6.1 Hz, 1H, H5), 3.87 (s, 6H, Ar'-OCH_3), 4.06 (dd, ^2J = 8.6 Hz, ^3J = 6.1 Hz, 1H, H5), 4.26 (dd, ^2J = 11.3 Hz, ^3J = 6.8 Hz, 1H, C3-CH), 4.40 (dd, ^2J = 11.4 Hz, ^3J = 7.0 Hz, 1H, C3-CH), 4.84 (d, ^3J = 6.2 Hz, 1H, H2), 6.11 (q, ^3J = 7.0 Hz, 1H, H3'''), 6.79 – 6.90 (m, 3H, Ar'-H), 6.98 (dd, ^3J = 8.6 Hz, $^3J_{H\text{-}F}$ = 8.6 Hz, 2H, H3'', H5''), 7.13 (dd, ^3J = 8.4 Hz, $^4J_{H\text{-}F}$ = 5.6 Hz, 2H, H2'', H6'').

^{13}C NMR (50 MHz, $CDCl_3$): δ 16.0 (q, C4'''), 20.7 (q, C5'''), 32.9 (t, C4-C), 42.6 (d, C4), 49.3 (d, C3), 56.0 (q, Ar'-OCH_3), 56.1 (q, Ar'-OCH_3), 62.2 (t, C3-C), 72.7 (t, C5), 82.9 (d, C2), 108.9 (d, C2'), 111.2 (d, C5'), 115.5 (dd, C3'', C5'', $^2J_{C\text{-}F}$ = 21.2 Hz), 118.1 (d, C6'), 127.4 (s, C2'''), 130.1 (dd, C2'', C6'', $^3J_{C\text{-}F}$ = 7.8 Hz), 135.0 (s, C1'), 135.8 (d, C1'', $^4J_{C\text{-}F}$ = 3.3 Hz), 139.2 (d, C3'''), 148.6 (s, C4'), 149.2 (s, C3'), 161.6 (d, C4'', $^1J_{C\text{-}F}$ = 244.2 Hz), 167.8 (s, C1''').

B.21 (*Z*)-((2*S*,3*R*,4*R*)-2-(3,4-Dimethoxyphenyl)-4-(4-methylbenzyl)tetrahydrofuran-3-yl)methyl 2- methylbut-2-enoate (33)

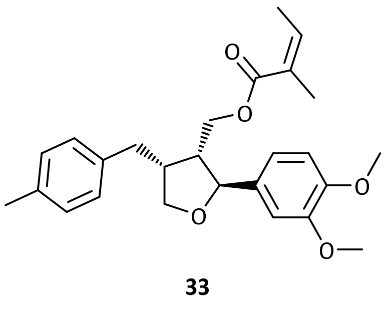

33

Chemical Formula: $C_{28}H_{32}O_5$
Molecular Weight: 425.53 gmol^{-1}

***Preparation**:* analogous to **27**, using starting material **25** (29.4 mg, 0.086 mmol, 1.00 equiv.). Et_2O (5 mL) was added to the reaction content, which was then filtered and rinsed with more Et_2O (10 mL). The solvents were evaporated and flash column chromatography (9 g silica, flow rate 20 mL/ min, EtOAc / LP, 1 : 99 to 35 : 65 in 30 min).

Yield: 30.8 mg, (84 %)

Appearance: colorless oil

***R*f (silica)**: 0.75 (EtOAc / LP, 1 : 1)

[α]D^{20}: +27.1 (c 1.92, MeOH)

LC-HRMS (ESI): calculated for M+Na$^+$: 447.2142, found: 447.2149, Δ: 1.57 ppm

(log *P*)calc: 6.10 ± 0.45

GC-MS (EI, 70 eV, Method D): 14.99 min; 424.3 (M$^+$, 3), 219.1 (26), 166.1 (50), 165.1 (100), 151.1 (19), 143.1 (18), 131.1 (27), 105.1 (76).

^{1}H NMR (200 MHz, CDCl$_3$): δ 1.84 – 1.90 (m, 3H, H5'''), 2.00 (dq, ^3J = 7.2 Hz, ^5J = 1.5 Hz, 3H, H4'''), 2.32 (s, 3H, C4''-CH$_3$), 2.51 – 2.84 (m, 3H, H3, H4, C4-CH), 2.91 (dd, ^2J = 12.4 Hz, ^3J = 4.2 Hz, 1H, C4-CH), 3.76 (dd, ^2J = 8.6 Hz, ^3J = 6.2 Hz, 1H, H5), 3.87 (s, 3H, Ar'-OCH$_3$), 3.87 (s, 3H, Ar'-OCH$_3$), 4.07 (dd, ^2J = 8.6 Hz, ^3J = 6.3 Hz, 1H, H5), 4.27 (dd, ^2J = 11.3 Hz, ^3J = 7.0 Hz, 1H, C3-CH), 4.41 (dd, ^2J = 11.3 Hz, ^3J = 7.0 Hz, 1H, C3-CH), 4.84 (d, ^3J = 6.2 Hz, 1H, H2), 6.10 (qq, ^3J = 7.2 Hz, ^4J = 1.4 Hz, 1H, H3'''), 6.78 – 6.91 (m, 3H, Ar'-H), 7.03 – 7.14 (m, 4H, Ar''-H).

^{13}C NMR (50 MHz, CDCl$_3$): δ 16.0 (q, C4'''), 20.7 (q, C5'''), 21.1 (q, C4''-<u>C</u>H$_3$), 33.2 (t, C4-<u>C</u>), 42.6 (d, C4), 49.3 (d, C3), 56.0 (q, Ar'-OCH$_3$), 56.1 (q, Ar'-OCH$_3$), 62.3 (t, C3-<u>C</u>), 72.9 (t, C5), 83.0 (d, C2), 108.9 (d, C2'), 111.1 (d, C5'), 118.1 (d, C6'), 127.5 (s, C2'''), 128.6 (d, C2'', C6''), 129.4 (d, C3'', C5''), 135.1 (s, C1'), 135.9 (s, C4''), 137.1 (s, C1''), 139.0 (d, C3'''), 148.5 (s, C4'), 149.2 (s, C3'), 167.8 (s, C1''').

B.22 (*Z*)-((2*S*,3*R*,4*R*)-4-(4-(*tert*-Butyl)benzyl)-2-(3,4-dimethoxyphenyl)tetrahydro furan-3-yl)methyl 2-methylbut-2-enoate (34)

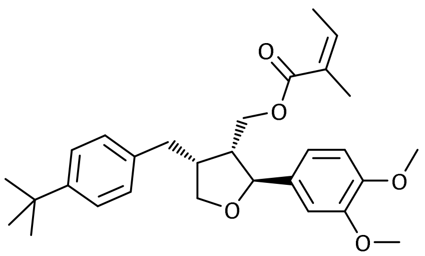

34

Chemical Formula: $C_{29}H_{38}O_5$
Molecular Weight: 466.61 gmol^{-1}

***Preparation**:* analogous to **27**, using starting material **26** (31.1 mg, 0.081 mmol, 1.00 equiv.). Et$_2$O (5 mL) was added to the reaction content, which was then filtered and rinsed with more Et$_2$O (15 mL). The solvents were evaporated and flash column chromatography (9 g silica, flow rate 20 mL/ min, EtOAc / LP, 7: 93 isocratically for 3 min, then to 35: 65 in 30 min).

Yield: 25.4 mg, (67 %)

Appearance: colorless oil

***R*f (silica)**: 0.78 (EtOAc / LP, 1:1)

[α]$_D$^{20}: +21.4 (c 1.52, MeOH)

LC-HRMS (APCI): calculated for M+Na$^+$: 489.2611, found: 489.2676, Δ: 13.29 ppm

(log *P*)$_{calc}$: 7.33 ± 0.46

GC-MS (EI, 70 eV, Method E): 19.94 min; 466.3 (M$^+$, 2), 219.1 (37), 185.1 (20), 180.1 (16), 166.1 (37), 165.1 (100), 151.1 (20), 147.1 (19), 132.1 (16), 131.1 (15), 117.1 (25).

^{1}H NMR (200 MHz, CDCl$_3$): δ 1.31 (s, 9H, C4''-C(CH$_3$)$_3$), 1.85 – 1.90 (m, 3H, H5'''), 2.00 (dq, ^3J = 7.2 Hz, ^5J = 1.5 Hz, 3H, H4'''), 2.53 – 2.86 (m, 3H, H3, H4, C4-CH), 2.91 (dd, ^2J = 12.4 Hz, ^3J = 4.2 Hz, 1H, C4-CH), 3.80 (dd, ^2J = 8.5 Hz, ^3J = 6.3 Hz, 1H, H5), 3.87 (s, 3H, Ar'-OCH$_3$), 3.88 (s, 3H, Ar'-OCH$_3$), 4.09 (dd, ^2J = 8.6 Hz, ^3J = 6.3 Hz, 1H, H5), 4.28 (dd, ^2J = 11.3 Hz, ^3J = 7.0 Hz, 1H, C3-CH), 4.42 (dd, ^2J = 11.4 Hz, ^3J = 7.0 Hz, 1H, C3-CH), 4.84 (d, ^3J = 6.3 Hz, 1H, H2), 6.10 (qq, ^3J = 7.2 Hz, ^4J = 1.4 Hz, 1H, H3'''), 6.79 – 6.92 (m, 3H, Ar'-H), 7.10 (d, ^3J = 8.3 Hz, 2H, H2'', H6''), 7.32 (d, ^3J = 8.3 Hz, 2H, H3'', H5'').

^{13}C NMR (50 MHz, CDCl$_3$): δ 16.0 (q, C4'''), 20.7 (q, C5'''), 31.5 (q, C4''-C($\underline{C}$H$_3$)$_3$), 33.1 (t, C4-$\underline{C}$), 34.5 (s, C4''-$\underline{C}$(CH$_3$)$_3$), 42.5 (d, C4), 49.3 (d, C3), 56.0 (q, Ar'-OCH$_3$), 56.1 (q, Ar'-OCH$_3$), 62.4 (t, C3-$\underline{C}$), 73.0 (t, C5), 83.0 (d, C2), 109.0 (d, C2'), 111.1 (d, C5'), 118.2 (d, C6'), 125.6 (d, C3'', C5''), 127.5 (s, C2'''), 128.4 (d, C2'', C6''), 135.2 (s, C1'), 137.1 (s, C1''), 139.0 (d, C3'''), 148.6 (s, C4'), 149.18 (s, C3'*), 149.21 (s, C4''*), 167.9 (s, C1''').

References

1. Schwetlick, K., *Organikum*. 22 ed. 2004, Weinheim: Wiley-VCH.
2. Simas, A.B.C., et al., *An expeditious and consistent procedure for tetrahydrofuran (THF) drying and deoxygenation by the still apparatus.* Quim. Nova, 2009. **32**(9): p. 2473-2475.
3. Mandal, P.K., G. Maiti, and S.C. Roy, *Stereoselective Synthesis of Polysubstituted Tetrahydrofurans by Radical Cyclization of Epoxides Using a Transition-Metal Radical Source. Application to the Total Synthesis of (±)-Methylenolactocin and (±)-Protolichesterinic Acid.* J. Org. Chem., 1998. **63**(9): p. 2829-2834.
4. Gao, Y., et al., *Catalytic asymmetric epoxidation and kinetic resolution: modified procedures including in situ derivatization.* J. Am. Chem. Soc., 1987. **109**(19): p. 5765-5780.
5. Gottlieb, H.E., V. Kotlyar, and A. Nudelman, *NMR chemical shifts of common laboratory solvents as trace impurities.* J. Org. Chem., 1997. **62**(21): p. 7512-7515.
6. Fulmer, G.R., et al., *NMR Chemical Shifts of Trace Impurities: Common Laboratory Solvents, Organics, and Gases in Deuterated Solvents Relevant to the Organometallic Chemist.* Organometallics, 2010. **29**(9): p. 2176-2179.
7. Linder, T., et al., *Leoligin-inspired synthetic lignans with selectivity for cell-type and bioactivity relevant for cardiovascular disease.* Chem Sci, 2019. **10**(22): p. 5815-5820.
8. Chakraborty, P., et al., *Titanocene(III) chloride mediated formal synthesis of magnofargesin and 7â€²-epimagnofargesin.* Tetrahedron Lett. **53**(48): p. 6584-6587.
9. Schwaiger, S., et al., *New constituents of Leontopodium alpinum and their in vitro leukotriene biosynthesis inhibitory activity.* Planta Med., 2004. **70**(10): p. 978-985.
